# A Multi-Omics Analysis Revealed the Diversity of the MYB Transcription Factor Family’s Evolution and Drought Resistance Pathways

**DOI:** 10.3390/life14010141

**Published:** 2024-01-18

**Authors:** Fan Zhang, Jie Ma, Ying Liu, Jing Fang, Shuli Wei, Rui Xie, Pingan Han, Xiaoqing Zhao, Suling Bo, Zhanyuan Lu

**Affiliations:** 1 Inner Mongolia Academy of Agricultural & Animal Husbandry Sciences, Hohhot 010031, China; zhangfan98hl@163.com (F.Z.); majie19952021@163.com (J.M.); liuying836354822@126.com (Y.L.); fangjing721@126.com (J.F.); wslweishuli@163.com (S.W.); xierui12358@163.com (R.X.); hanpingan327@163.com (P.H.); 2 Key Laboratory of Black Soil Protection and Utilization, Ministry of Agriculture and Rural Areas, Hohhot 010031, China; 3 Inner Mongolia Key Laboratory of Degradation Farmland Ecological Remediation and Pollution Control, Hohhot 010031, China; 4 Inner Mongolia Conservation Tillage Engineering Technology Research Center, Hohhot 010031, China; 5 School of Life Science, Inner Mongolia University, Hohhot 010030, China; 6 Key Laboratory of Herbage & Endemic Crop Biotechnology, Ministry of Education, Hohhot 010030, China; 7 College of Computer Information, Inner Mongolia Medical University, Hohhot 010110, China

**Keywords:** MYB transcription factor, drought, transcriptome, evolution, bioinformatics

## Abstract

The MYB transcription factor family can regulate biological processes such as ABA signal transduction to cope with drought stress, but its evolutionary mechanism and the diverse pathways of response to drought stress in different species are rarely reported. In this study, a total of 4791 MYB family members were identified in 908,757 amino acid sequences from 12 model plants or crops using bioinformatics methods. It was observed that the number of MYB family members had a linear relationship with the chromosome ploidy of species. A phylogenetic analysis showed that the MYB family members evolved in subfamily clusters. In response to drought stress, the pathways of MYB transcription factor families exhibited species-specific diversity, with closely related species demonstrating a higher resemblance. This study provides abundant references for drought resistance research and the breeding of wheat, soybean, and other plants.

## 1. Introduction

Drought is one of the most important abiotic stresses affecting crop growth and yield. Globally, the annual agricultural losses caused by drought rank first among all agricultural disasters [1,2]. Screening and identifying major drought-resistant genes, deciphering the adaptive mechanism of plants to drought, and subsequently cultivating new varieties of crops with high drought resistance are essential ways to address the drought crisis [3,4]. The genes that help plants resist the harm of drought can be divided into two types: one is the functional genes, whose encoded proteins directly play a protective role in plant cells, such as aquaporin (AQP) [5,6] and late embryogenesis abundant protein (LEA) [7,8]; another is regulatory genes, encoding products that can transmit signals and regulate the expression of related functional genes in the process of plant drought resistance, such as protein kinases and transcription factors [9,10].

The MYB (V-myb avian myeloblastosis viral oncogene homolog) transcription factors family is one of the largest transcription factor families in plants [11]. The MYB transcription factor consists of a transcription activation region, a negative regulation region, and a DNA binding region, where the amino acid composition is highly conservative and comprises a characteristic domain (namely the MYB domain) [12]. The MYB domain contains around one to four incomplete repeat sequences, each of which consists of 50~53 conservative amino acid residues, forming a helix–turn–helix structure [13]. Typically, there are three regularly spaced tryptophan residues (Try, W) in each MYB repeat that participate in a hydrophobic cluster which may be involved in DNA-specific recognition [14,15]. According to the location and number of repetitive sequences in the MYB domain, MYB transcription factors can be divided into four subfamilies, 1R-MYB (MYB-related), 2R-MYB (R2R3-MYB), 3R-MYB, and 4R-MYB [16].

When plants encounter drought stress, MYB transcription factors can rapidly regulate the expression of related functional genes and initiate a series of physiological and biochemical reactions through its signal transduction network, finally reducing or eliminating the damage of drought to plants [17,18]. For example, *GhMYB3*, a member of the R2R3-MYB subfamily of upland cotton, can regulate stomatal closure and active oxygen accumulation activities in a negatively regulated manner, thereby playing a role in the response of the plant to drought stress [19]. The overexpression of MYB family members can improve the tolerance of transgenic plants to drought stress, especially for crops to improve their yield and promote agricultural productivity, with great potential. For example, the overexpression of *CcMYB107*—a member of the *Cajanus cajan* R2R3-MYB subfamily—enhances the antioxidant enzyme activity of the plant and increases the accumulation of proline and lignin, thereby enhancing the drought resistance of *Cajanus cajan* [20]. The MYB transcription factor *AgMYB5,* extracted from celery, enhances the antioxidant enzyme activity of transgenic *Arabidopsis thaliana* by promoting the biosynthesis of β-carotene, and promotes the biosynthesis of endogenous ABA, leading to ABA-induced stomatal closure and improving drought resistance [21].

However, the current research on the MYB transcription factor family mainly focuses on single species, and there are few reports on the diversity of its inter-species regulatory pathways. Therefore, to explore the inter-specific evolution mechanism of MYB transcription factors and the diversity of pathways regulating plant drought resistance, this study took 12 model plants or crops, including *Arabidopsis thaliana*, wheat, potato, soybean, and flax as samples. The evolutionary diversity of the MYB transcription factor family was analyzed based on genomic data, and the expression pattern differences of MYB families under drought stress were analyzed based on transcriptomic data. Furthermore, the diversity of interspecific pathways responding to drought was explored and the correlation between evolution and its regulation pathways was discussed.

## 2. Materials and Methods

### 2.1. Materials

#### 2.1.1. Genomic Data

In this study, three gramineous plants, including wheat (*Triticum awstivum*), rice (*Oryza sativa*), and millet (*Setaria italica*); three leguminous plants, including soybean (*Glycine max*), cultivated peanut (*Arachis hypogaea*), and barrel medic (*Medicago truncatula*); three Solanaceae plants, including tomato (*Solanum lycopersicum*), potato (*Solanum tuberosum*), and tobacco (*Nicotiana tabacum*); the model plants *Arabidopsis thaliana* and outgroup grape (*Vitis vinifera*); and the drought-tolerant plant flax (*Linum usitatissimum*) were screened [22]. The genomic data of a total of 12 species were used in this study (Table 1).

#### 2.1.2. Transcriptomic Data

Five model plants or crops were selected from the SRA database: the model plant, *Arabidopsis thaliana*; gramineous plants, wheat (*Triticum aestivum*) and rice (*Oryza sativa*); a leguminous plant, soybean (*Glycine max*); and a solanaceous plant, potato (*Solanum tuberosum*).

The selection criteria of the transcriptome were as follows: (1) the sampling organ was the root; (2) the sequencing platform was Illumina; (3) the data type was RNA-seq; and (4) the treatment measures should include at least a drought group and a control group, with at least three replicates in each group. If there were multiple drought groups, the severe drought group was chosen in this study (Table 2).

### 2.2. Data Processing Methods

#### 2.2.1. Construction of MYB Transcription Factor Family Database

##### Identification of MYB Family Members

Members of the MYB family were identified based on a conservative domain—the MYB domain (PF00249). The HMMER-3.0 software [23] was used to search the amino acid sequences in the 12 species to identify members of the MYB gene family, and the characteristics of the conservative domain were determined according to the following criteria: (1) a conditional E-value value < 1 × 10^−5^; and (2) the conservative sequence contains at least one conservative tryptophan [24,25]. According to the GFF file of each species, the genes obtained after the HMMER search and the deletion of repetitive sequences were counted as members of the MYB family. The IDs of all the MYB genes were simplified by removing special symbols such as “_”, which were not recognized by the analysis software.

##### Subfamily Division of MYB Members 

According to the number of MYB domains, the family was divided into four subfamilies: 1R-MYB (MYB-related), 2R-MYB (R2R3-MYB), 3R-MYB, and 4R-MYB. Among them, the two MYB domains of the 2R-MYB subfamily members could not be more than 10 amino acids apart, and if they were, then the members would be classified into the R-R-type subfamily of 1R-MYB [24,26].

##### Phylogenetic Analysis of Interspecies MYB Family

The phylogenetic algorithm of the OrthoFinder-2.5.4 software [27,28] was used to confirm the gene homology, and then STAG algorithm was used to construct a species tree. Taking the members of the 3R-MYB and 4R-MYB subfamilies with reduced numbers and conservative evolution as representatives, the genetic distance between these genes was calculated using a Neighbor-joining (NJ) algorithm, and a typical phylogenetic tree of members with typical MYB domains was constructed. The software used was MEGA-11 [29] and the bootstrap repeat value was 1000. The obtained phylogenetic tree was uploaded to the Evolview website [30] (http://www.evolgenius.info/evolview) (accessed from 1 January 2021 to 31 May 2023) for annotation and landscaping.

#### 2.2.2. Analysis of Expression Modules of Interspecies MYB Family

##### Data Quality Control and Sequence Assembly

The FastQC-0.00.9 software was used to detect the quality of all fastq sequencing files of the 5 projects (Table 2), and Trimmomatic-0.39 [31] was used to trim the adapter, preserving the integrity of the original sequence to the greatest extent possible in the process. The fastq sequencing files of each species with completed quality control were compared to their respective reference genomes by using the Kallisto-0.48.0 software [32].

##### DEGs (Differential Expression Genes) of MYB Family Screening

The tximportData-1.24.0 package was used to import the sample information, and the tximport package was used to import the TSA file of comparison results with Kallisto-0.48.0. The DESeq2-1.36.0 package was used to analyze the relative expression values of the samples and screen the DEGs between the drought group and the control group within the MYB family. The screening criteria were a *p*-value < 0.05 and a |log2FoldChange| > 1.

##### DEG Function Analysis

We uploaded the screened amino acid sequence of the DEGs of each project to the genome function annotation website Eggnog-Mapper (http://eggnog-mapper.embl.de/) (accessed from 1 January 2023 to 13 February 2023), including the GO annotation and KEGG annotation. The TBtools-2.019 software [33] was used to conduct classified statistics on the analysis results. The GO categorical histogram tool in Majorbio Cloud Platform (https://cloud.majorbio.com/page/tools/) (accessed from 10 February 2023 to 21 April 2023) was used to visualize the GO annotation results, selecting the top 20 abundant GO terms and some special GO terms for presentation.

## 3. Results

### 3.1. MYB Transcription Factor Family Database

#### 3.1.1. MYB Family Members’ Distribution

In this study, a total of 4791 MYB family members were identified in 908,757 amino acid sequences from 12 species, and the number of MYB family members exhibited significant interspecies variation, ranging from 258 to 610 (Table 3, Supplementary Tables S1–S12). Among them, the number of rice (*Oryza sativa*, 258) MYB members was the least, which was less than that of the model species, *Arabidopsis thaliana* (272), and the evolutionary conservative species, grape (*Vitis vinifera*, 269) [34]. The number of MYB family members in soybean (*Glycine max*, 610), wheat (*Triticum aestivum*, 608), tobacco (*Nicotiana tabacum*, 536), and cultivated peanut (*Arachis hypogaea*, 541) was more than two times that in rice (*Oryza sativa*, 258), *Arabidopsis thaliana* (272) and grape (*Vitis vinifera*, 269). Since soybeans, wheat, tobacco, and cultivated peanuts are tetraploid or hexaploid, while plants such as rice, *Arabidopsis thaliana*, and grape are diploid, it was speculated that the increase in the number of members was caused by chromosome doubling, and the number of MYB members showed a linear relationship with the chromosome ploidy of the species.

The number of members of MYB subfamilies also differed significantly among species (Table 3). With the increase in the number of domains, the number of subfamily members was gradually reduced; for example, there were 135~357 members in the 1R-MYB subfamily, 108~294 members in the 2R-MYB subfamily, 4~11 members in the 3R-MYB subfamily, and 0~3 members in the 4R-MYB subfamily. Rice and flax each contained one MYB member, with five MYB conserved domains.

#### 3.1.2. Diversity of Evolutionary Trends among MYB Families

**Figure 1:**
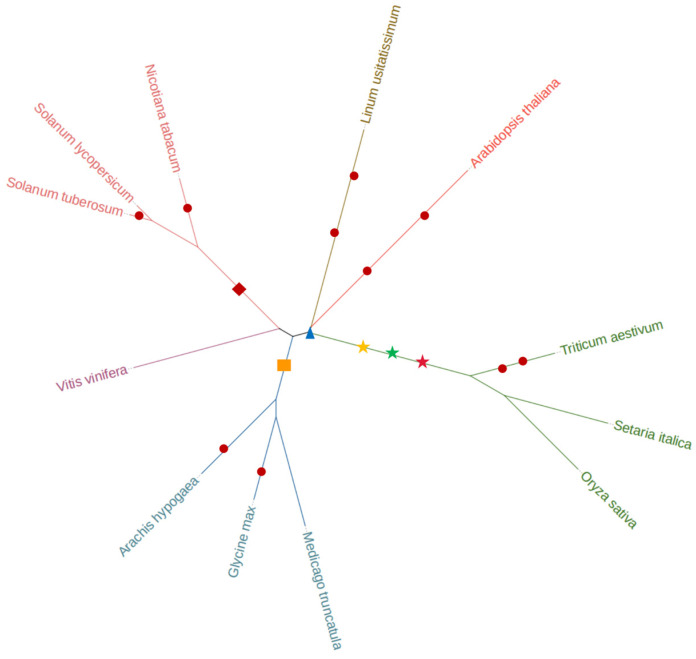
Species tree based on MYB family genes. Note: 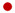 stands for genome-wide doubling event, 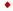 stands for genome-wide tripling event, 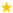 stands for genome-wide multiploidy event (σ) experienced by monocotyledon, 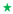 stands for genome-wide multiploidy event (τ) experienced by monocotyledon, 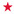 stands for genome-wide multiploidy event (γ) experienced by monocotyledon, 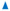 stands for genome-wide multiploidy event experienced by dicotyledon, 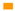 stands for genome-wide multiploidy event experienced by legume.

A species tree (Figure 1) based on the amino acid sequences of all MYB family members from 12 species was constructed to explore species kinship. The results showed that the MYB families of the same family were more closely related. In addition, soybean (*Glycine max*) in the Leguminosae family had a closer genetic relationship with barrel medic (*Medicago truncatula*); potato (*Solanum tuberosum*) in the Solanaceae family had a closer genetic relationship with tomato (*Solanum lycopersicum*); rice (*Oryza sativa*) and millet (*Setaria italica*) in the Gramineae family had a closer genetic relationship; and the species that had the closest genetic relationship with flax (*Linum usitatissimum*) was *Arabidopsis thaliana*. The genetic distance between the MYB family of grape (*Vitis vinifera*) and the other 11 species was far, while that of Gramineae was also distantly separated from other species in the evolutionary tree.

**Figure 2:**
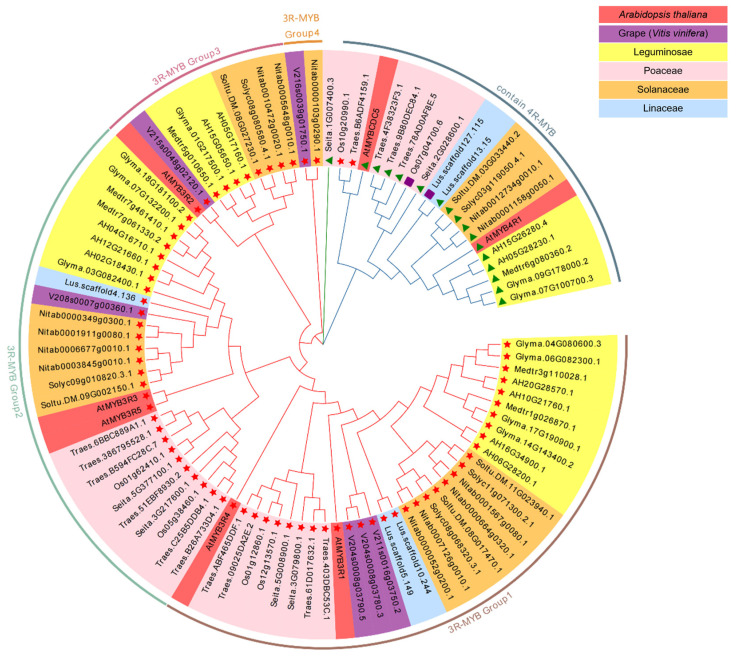
Genetic phylogenetic tree of multiple MYB-domain genes. Symbols on the phylogenetic tree represent subfamilies of MYB genes. 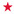 represents members of the 3R-MYB subfamily, 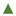 represents members of the 4R-MYB subfamily, and 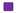 represents members of the 5R-MYB subfamily (containing five conserved domains of MYB).

The phylogenetic tree was constructed for members containing multiple MYB domains, including 3R-MYB and 4R-MYB, and two genes containing five MYB domains (*Os07g04700.6* and *Lus.scaffold127.115*) in all 12 species, to explore the genetic relationship of multiple MYB domain genes among species (Figure 2). 

The results showed that members of the MYB family evolved in subfamily clusters. Members of the 3R-MYB and 4R-MYB subfamilies were divided into two branches, suggesting that there is a significant genetic distance between 3R-MYB subfamily members and those of 4R-MYB, and that differentiation among MYB subfamilies precedes differentiation among these 12 species. Subsequently, members of the 3R-MYB subfamily were further divided into four groups in the evolutionary tree. The intraspecific homologous genes in the branches of each group were clustered preferentially; then, the homologous genes of the same family were clustered, and finally, the homologous genes were clustered with other families according to the interspecific relationship. The genes *Os07g04700.6* and *Lus.scaffold127.115*, containing five MYB domains, were both clustered with the 4R-MYB gene, indicating a close genetic relationship with the 4R-MYB gene. 

### 3.2. Interspecific MYB Family Expression Profile Diversity 

#### 3.2.1. Expression Profile of MYB Family in *Arabidopsis thaliana*

##### Expression of *Arabidopsis thaliana* MYB Family

**Figure 3:**
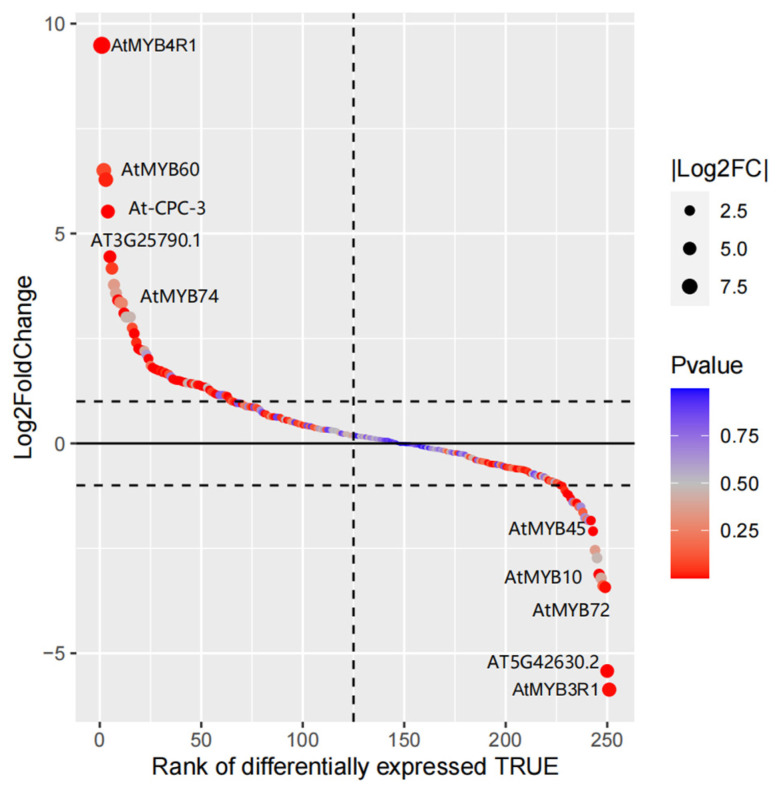
Gene differential expression sequencing of *Arabidopsis thaliana* MYB family members. Each circle represents one MYB family gene; the size of the circle represents the log2FoldChange value of the gene; and the color of the circle represents the *p*-value of the gene.

A total of 49 DEGs were found in the *Arabidopsis thaliana* MYB family under drought stress, among which 37 DEGs were up-regulated, including 18 members in the 2R-MYB subfamily and 18 members in the 1R-MYB subfamily. *AtMYB4R1*, which contains four MYB conservative domains, was the most significant member of the *Arabidopsis thaliana* MYB family for up-regulation under drought stress. In addition, the up-regulation of *AtMYB60* and *AtMYB74* in the 2R-MYB subfamily and *At-CPC-3* and *AT3G25790.1* in the 1R-MYB subfamily were also significant (Figure 3, Supplementary Table S13).

Twelve DEGs were significantly down-regulated under drought stress, including seven members in the 2R-MYB subfamily and four members in the 1R-MYB subfamily. *AtMYB3R1*, which contains three conservative domains of MYB, was the most significant member of the *Arabidopsis thaliana* MYB family for down-regulation under drought stress. In addition, the down-regulations of *AtMYB72*, *AtMYB10*, and *AtMYB45* in the 2R-MYB subfamily and *AT5G42630.2* in the 1R-MYB subfamily were also significant (Figure 3, Supplementary Table S13).

##### Functional Annotation of DEGs in *Arabidopsis thaliana* MYB Family

**Figure 4:**
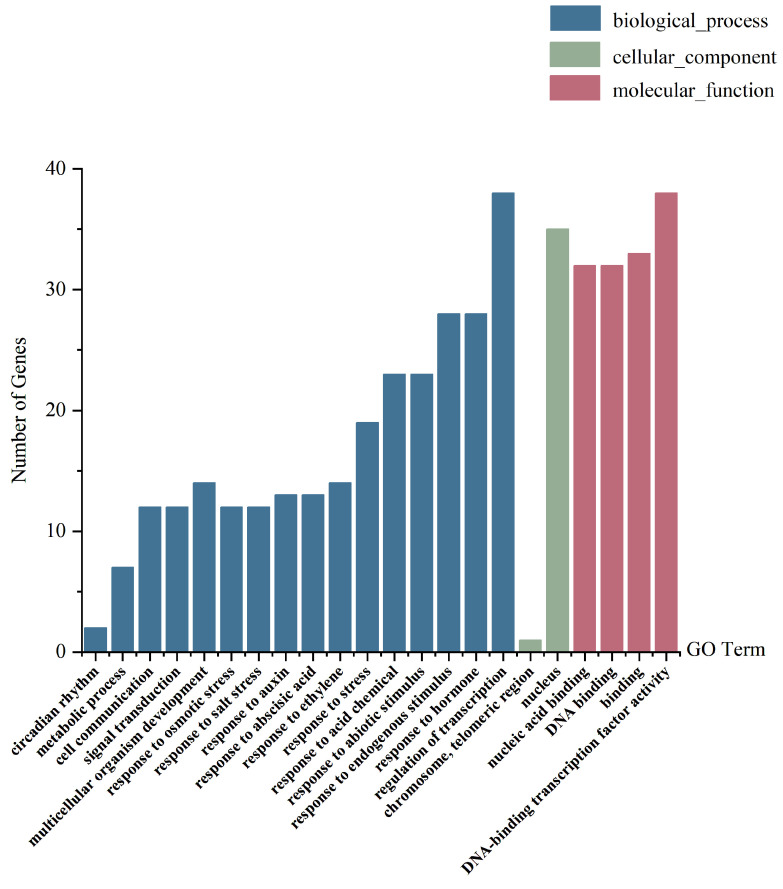
GO classification of the DEGs of the *Arabidopsis thaliana* MYB family.

The DEGs of the *Arabidopsis thaliana* MYB family were annotated in GO and KEGG, and classified according to the results of the GO annotations (Figure 4). The results showed that there were 39 DEGs annotated for their molecular function and biological process. The annotated molecular functions were essentially related to DNA-binding transcription factor activity. For biological processes, the most frequently annotated term of GO was the regulation of transcription, which was in line with the basic function of the “transcriptional regulation” of transcription factors. The transcription regulation function of DEGs of the *Arabidopsis thaliana* MYB family may be triggered by external or internal stimuli, such as osmotic stress.

In addition, some DEGs were annotated for biological processes such as signal transduction, cell communication, metabolic processes, and circadian rhythm. Twelve DEGs were annotated for the signal transduction process, in which three members of the 2R-MYB subfamily, *AtMYB10*, *AtMYB63,* and *AtMYB72*, could negatively regulate the ethylene-mediated signaling pathway. Twelve DEGs were annotated for the process of multicellular organism development, in which *AtMYB88*, a member of the 2R-MYB subfamily, could positively regulate gametophyte development. Twelve DEGs were annotated for the process of cell communication, in which three members of the 1R-MYB subfamily, *AT1G13300.1*, *AT1G25550.1,* and *AT3G25790.1*, could positively regulate the cell response to phosphate starvation—the process in which the cell state is changed due to a lack of phosphate. Seven DEGs were annotated for the metabolic process, in which four members of the 2R-MYB subfamily, *AtMYB6*, *AtMYB10*, *AtMYB63*, and *AtMYB72*, could regulate phenylpropanoid biosynthesis. Two homologous DEGS from the 1R-MYB subfamily (*AT-CCA1-16*, *AT5G17300.1*) were annotated for the circadian rhythm process (Figure 4, Supplementary Table S13).

The annotated KEGG pathways of the *Arabidopsis thaliana* MYB family included plant hormone signal transduction (ko04075) and the PI3K-Akt signaling pathway (ko04151). *AT1G67710.1* was annotated for the plant hormone signal transduction pathway, and *AtMYB3R1*, which showed significant differential expression under drought stress (Figure 3), was annotated for the PI3K-Akt signaling pathway.

##### Main Drought Resistance Pathways of *Arabidopsis thaliana* MYB Family

The DEG functional annotation results showed that members of the MYB family in *Arabidopsis thaliana* mainly regulated the expression of genes such as *AtMYB4R1*, *AtMYB3R1*, *AtMYB6*, *AtMYB10*, *AtMYB63*, and *AtMYB72*, so as to complete cell communication in response to phosphate starvation, as well as hormone signal transduction such as ethylene, phenylpropanoid biosynthesis, the circadian rhythm, and other processes to resist drought stress (Figure 13).

#### 3.2.2. Expression Profiles of MYB Families in Gramineae Species

##### Expression Profile of MYB Family in Wheat (*Triticum aestivum*)

I. Expression of Wheat (*Triticum aestivum*) MYB Family

**Figure 5:**
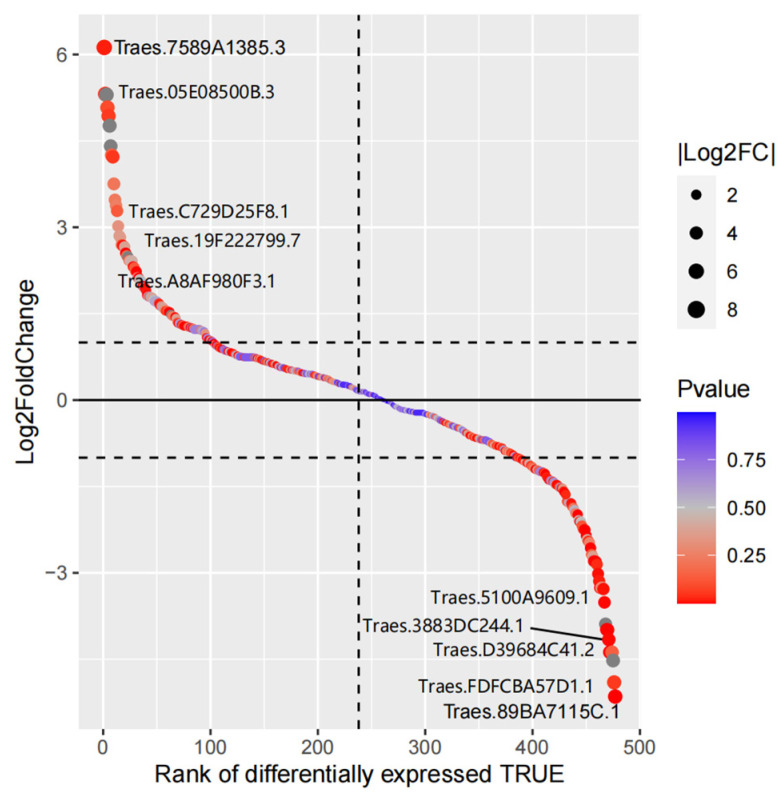
Gene differential expression sequencing of wheat MYB family members.

A total of 76 DEGs were found in the wheat MYB family under drought stress, with 30 DEGs up-regulated and 46 DEGs down-regulated. Among them, the 1R-MYB subfamily member *Traes.7589A1385.3* was the most significantly up-regulated gene under drought stress, whereas the 2R-MYB subfamily member *Traes.89BA7115C.1* was the most significantly down-regulated gene under drought stress (Figure 5, Supplementary Table S14).

II. Functional Annotation of DEGs in Wheat (*Triticum aestivum*) MYB Family

**Figure 6:**
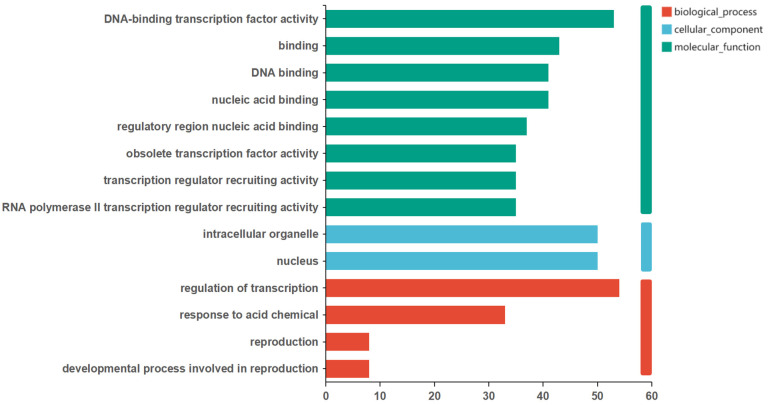
GO classification of wheat MYB family DEGs.

The results of the GO annotation for the DEGs in the wheat MYB family showed that some DEGs had the function of transcriptional co-regulator activity, because 35 DEGs were annotated under RNA polymerase II transcription regulator recruiting activity. In the latest interpretation of GO terms, the recruitment activities of these transcriptional regulators were all classified as being transcriptional co-regulator activity [35,36]. In addition, some DEGs were annotated under biological processes related to reproduction, for example, the *Traes.429583AEC.1* gene was annotated under the stamen development process (Figure 6).

The annotated KEGG pathways in wheat include plant hormone signal transduction (ko04075) and the circadian rhythm (ko04712). The 1R-MYB subfamily members *Traes.19F222799.7* and *Traes.310E46F15.7* were annotated under the Circadian rhythm pathway, both of which were homologous genes to the *Arabidopsis thaliana* circadian rhythm factors, CCA1 and LHY genes [37,38,39].

III. Main Drought Resistance Pathways of Wheat (*Triticum aestivum*) MYB Family

The results of functional annotation for DEGs indicated that members of the wheat MYB family, such as *Traes.19F222799.7* and *Traes.310E46F15.7*, could increase the activity of transcription co-regulatory factors and accelerate the development of stamen organs, circadian rhythm, hormone signal transduction, and other processes under drought stress (Figure 13).

##### Expression Profile of MYB Family in Rice (*Oryza sativa*)

I. Expression of Rice (*Oryza sativa*) MYB Family

**Figure 7:**
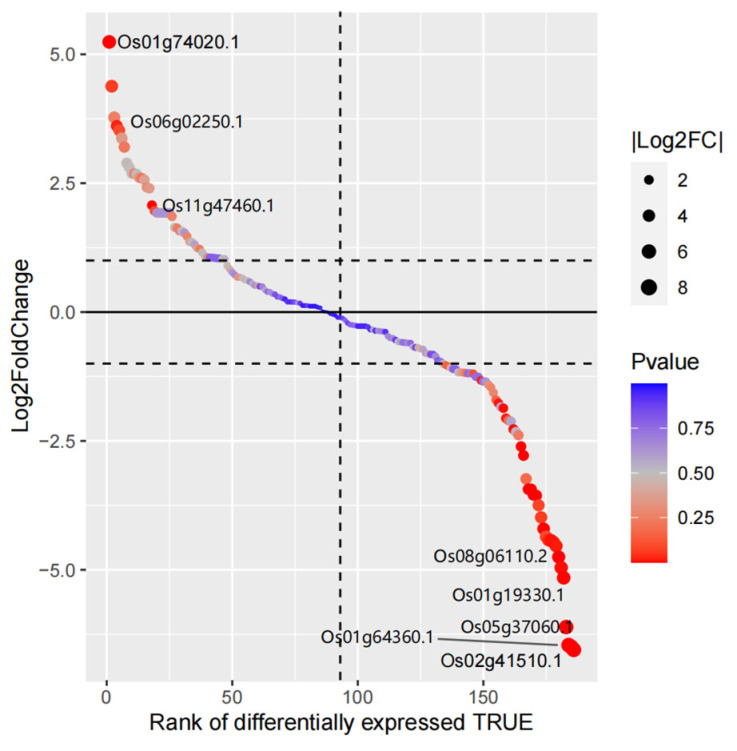
Gene differential expression sequencing of rice MYB family members.

There were 26 DEGs in the rice MYB families under drought stress, and only three of them were up-regulated. Of the DEGs with down-regulated expression, 15 were 2R-MYB subfamilies and eight were 1R-MYB subfamilies. Os02g41510.1 belongs to the 2R-MYB subfamily and is the most significantly down-regulated gene in the rice MYB family under drought stress (Figure 7, Supplementary Table S15).

II. Functional Annotation of DEGs in Rice (*Oryza sativa*) MYB Family

**Figure 8:**
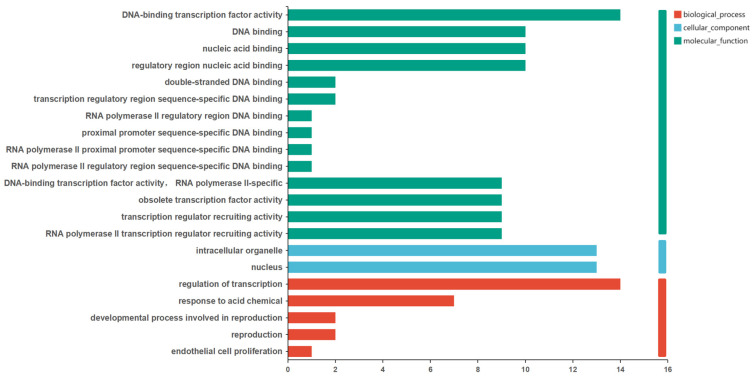
GO classification of the DEGs in rice MYB family.

The results of the GO annotation indicated that nine DEGs were annotated under RNA polymerase II-mediated DNA-binding transcription factor activity. In the 2R-MYB subfamily of rice, there were two up-regulated DEGs (*Os06g02250.1*, *Os11g47460.1*) annotated under RNA polymerase II transcription regulator recruiting activity. *Os01g16810.1* was annotated under the processes of endothelial cell proliferation and the reproduction processes of floral organ development (Figure 8, Supplementary Table S15). As there were also members of the wheat MYB family involved in reproductive processes for flower organ development (*Traes.429583AEC.1*), it was suggested that the MYB family of Gramineae may have a similar response to drought stress.

The annotated KEGG pathways in rice include plant hormone signal transduction (ko04075) and circadian rhythm (ko04712), in which *Os03g12350.1* was annotated for the plant hormone signal transduction pathway and *Os08g06110.2* was annotated for the circadian rhythm pathway.

III. Main Drought Resistance Pathways of Rice (*Oryza sativa*) MYB Family

The results of functional annotation for the DEGs indicated that when rice is subjected to drought stress, the MYB family mainly enhances the activity of transcription co-regulatory factors and regulates biological processes, such as the reproductive development of flower organs, plant hormone signal transduction, and circadian rhythm, as well as the coupled regulation of the RNA polymerase II-mediated transcriptional regulation process, by changing the expression levels of genes such as *Os08g06110.2* (Figure 13). 

#### 3.2.3. Expression Profile of MYB Family in Soybean (*Glycine max*)

##### Expression of Soybean (*Glycine max*) MYB Family

**Figure 9:**
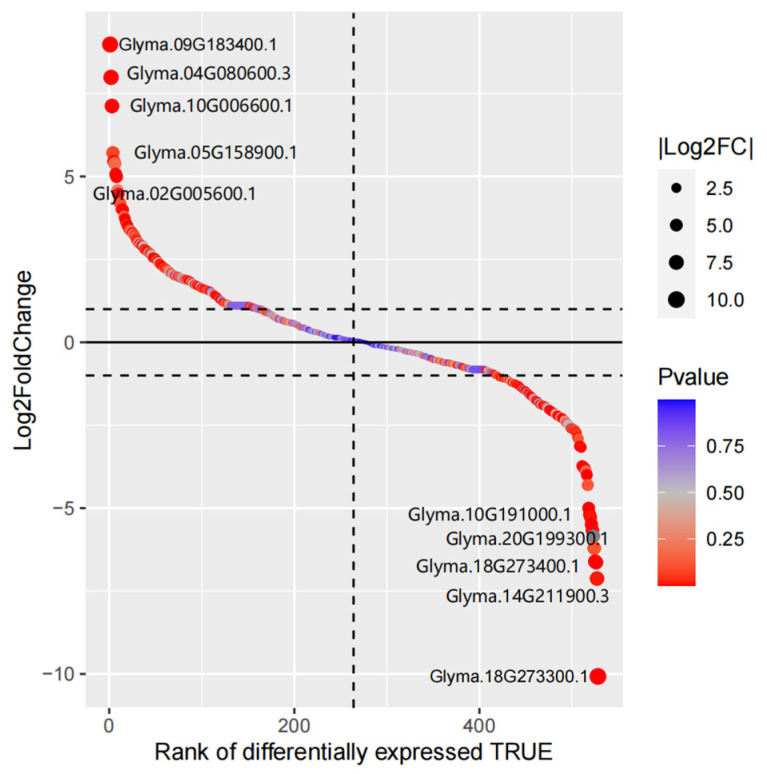
Gene differential expression sequencing of soybean MYB family members.

Under drought stress, the soybean MYB family had a total of 126 DEGs, in which 64 were up-regulated, with *Glyma.09G183400.1* being the most significant up-regulation, and 62 were down-regulated, with *Glyma.18G273300.1* being the most significant down-regulation (Figure 9, Supplementary Table S16). 

##### Functional Annotation of DEGs in Soybean (*Glycine max*) MYB Family

**Figure 10:**
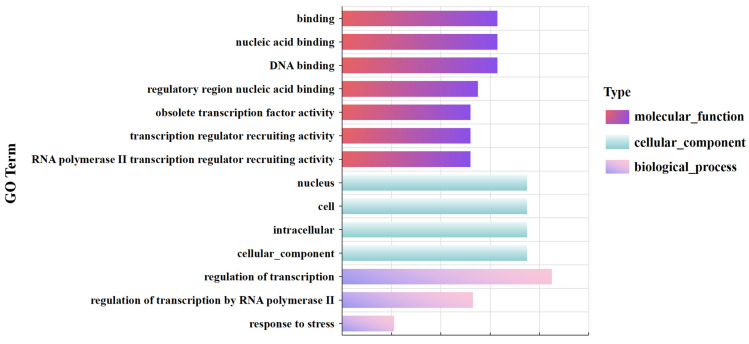
GO classification of the DEGs of the soybean MYB family.

The results of the GO annotation showed that among the molecular functions, there were 52 DEGs annotated for RNA polymerase II transcription regulator recruiting activity. Fifty-three soybean DEGs were annotated for RNA polymerase II-mediated transcriptional regulation. In addition, 21 DEGs were annotated to the process of responses to stress, and 75 DEGs were located in the nucleus (Figure 10).

The annotated KEGG pathways in soybean include plant hormone signal transduction (ko04075), the PI3K-Akt signaling pathway (ko04151), cell senescence (ko04218), and circadian rhythm (ko04712). *Glymea.04G080600.3*, a member of the 3R-MYB subfamily, was involved in the cellular senescence pathway, and *Glyma.03G261800.4*, a homologous gene of the *Arabidopsis thaliana* CCA1 and LHY genes in soybean, was annotated to the circadian rhythm pathway.

##### Main Drought Resistance Pathways of Soybean (*Glycine max*) MYB Family

The functional annotation results of the DEGs indicated that members of the soybean MYB family completed the activation of transcriptional co-regulatory activity by regulating the expression of genes such as *Glyma.04G080600.3*, and thus participated in biological processes and pathways such as RNA polymerase II-mediated transcriptional regulation, cell senescence, plant hormone signal transduction, the PI3K-Akt signaling pathway, circadian rhythm, and others to resist the harm of drought (Figure 13).

#### 3.2.4. Expression Profile of MYB Family in Potato (*Solanum tuberosum*)

##### Expression of Potato (*Solanum tuberosum*) MYB Family

**Figure 11:**
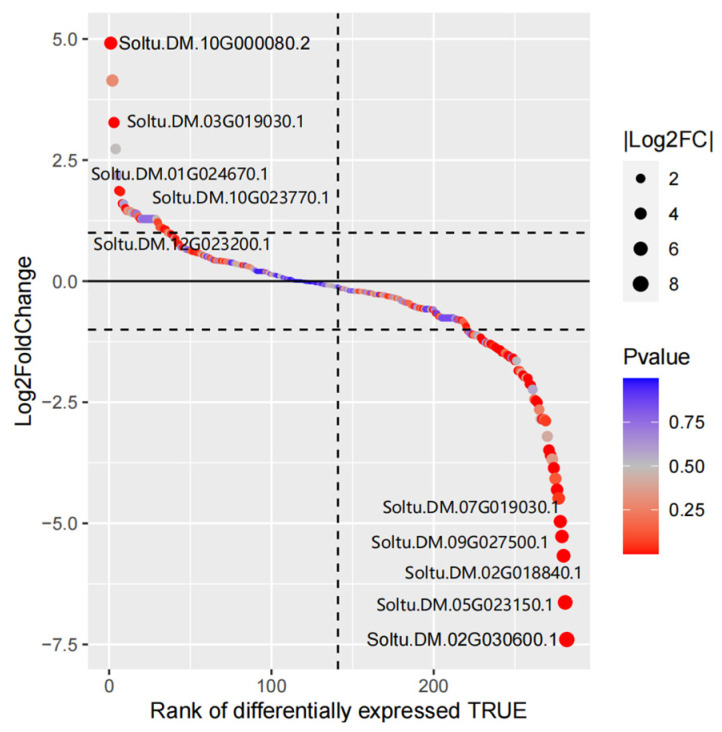
Gene differential expression sequencing of potato MYB family members.

There were 44 DEGs in the potato MYB family under drought stress, and nine up-regulated DEGs were identified. *Soltu.DM.10G000080.2* was the most significantly up-regulated under drought stress. There were 35 DEGs with down-regulated expression under drought stress, among which *Soltu.DM.02G030600.1* was the most down-regulated member of the potato MYB family (Figure 11, Supplementary Table S17).

##### Functional Annotation of DEGs in Potato (*Solanum tuberosum*) MYB Family

**Figure 12:**
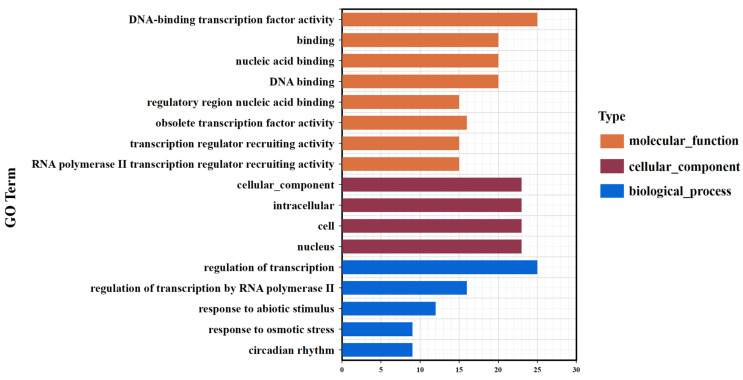
GO classification of the DEGs of the potato MYB family.

The results of the GO annotation showed that 16 DEGs were annotated to the regulation of transcription by RNA polymerase II, and five DEGs were annotated to the circadian rhythm process (Figure 12). Moreover, *Soltu.DM.05G010540.2*, a member of the 1R-MYB subfamily, was annotated to the plant hormone signal transduction pathway (ko04075) of the KEGG.

##### Main Drought Resistance Pathways of Potato (*Solanum tuberosum*) MYB Family

**Figure 13:**
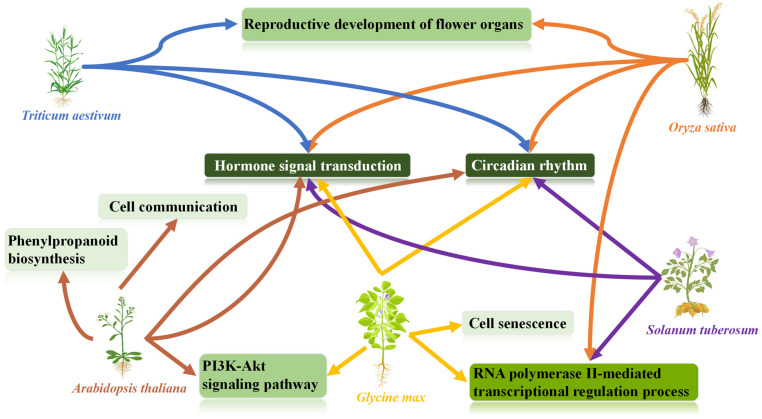
Pathways regulated by MYB transcription factor family members under drought stress. This figure is based on the GO and KEGG annotation results of DEGs belonging to the MYB family across five species under drought stress. Each green square represents a biological process to which DEGs have been annotated, and the darker the color, the more species of the MYB family have DEGs annotated to that biological process. This figure illustrates the biological processes associated with these annotated DEGs.

The functional annotation results of the DEGs indicated that members of the potato (*Solanum tuberosum*) MYB family completed the activation of transcriptional co-regulatory factors and RNA polymerase II-mediated transcriptional regulation, circadian rhythm, and plant hormone signal transduction, as well as other stress responses to resist the drought hazard by regulating the expression of genes such as *Soltu.DM.05G010540.2* (Figure 13).

## 4. Discussion

### 4.1. Evolutionary Diversity of MYB Family

The MYB family is a large family of transcription factors that can participate in abiotic stress responses in plants [40,41]. In this study, a total of 4791 MYB genes were identified in the whole genome of 12 species (Table 3). It was found that the MYB family was diverse in number, distribution, and evolutionary history in different species. This is similar to the results of a large number of previous studies; for example, the number of MYB family members in seven species of the Dioscorea genus is different, and the number of *Ipomoea trifida* (430) MYB genes is nearly double that of *Ipomoea purpurea* (226) [42].

The number of rice (*Oryza sativa*) MYB family members (258) was lower than that of the model species *Arabidopsis thaliana* (272) and grape (*Vitis vinifera*, 269), suggesting that member loss occurred in the rice MYB family during evolution (Table 3). By analyzing the *OsIRL* gene family in the rice genome, a previous study also concluded that the large number of gene losses in the rice genome affected the number of members in the gene family [43], which is consistent with the results of this study. The soybean (*Glycine max*) MYB family has the largest number of members (610, Table 3); this was speculated to be related to a single polyploidization event in the soybean genome [44]. In addition, it was confirmed that fragment replication also contributed to the amplification of the MYB gene family, which might be the reason for the amplification of the MYB family in soybean [45,46]. The members of the MYB family of wheat (*Triticum aestivum*, 608), tobacco (*Nicotiana tabacum*, 536), and cultivated peanut (*Arachis hypogaea*, 541) were also more abundant (Table 3), suggesting that the integrated hybridization of the ancestral genomes may result in multiple sets of chromosome in their genomes [47,48], triggering the multiplication of the MYB family.

In this study, by establishing a phylogenetic tree using MYB family genes, we found that the genetic distance of Gramineae from other species in the evolutionary tree was far, which well-distinguished the three species of Gramineae as monocotyledons from other dicotyledonous plants (Figure 1). About 150 million years ago, monocotyledons and dicotyledons were differentiated [49]; then, monocotyledonous plants together experienced two whole-genome duplication events (σ [50] and τ [51]). Gramineous plants have since collectively experienced one whole-genome duplication event [52,53]. And 130 million years ago, after an ancient whole-genome tripling event (γ [51,54]; eudicot-common triplication, ECT), the main groups of dicotyledonous plants began to differentiate and appear, after which many species experienced multiple whole-genome duplication events. For example, flax (*Linum usitatissimum*) underwent two whole-genome duplication events [55]. 

In the phylogenetic tree constructed with 136 *Cymbidium ensifolium* MYB genes and 131 *Arabidopsis thaliana* MYB genes, individual clusters of genes from different subfamilies were formed [56]. In this study, there was a significant genetic distance between 3R-MYB and 4R-MYB, and the differentiation among subfamilies was prior to species differentiation, which was consistent with the phenomenon found by previous researchers (Figure 2). A phylogenetic analysis of the 1R-MYB (MYB-related) subfamily in 16 species revealed that the MYB-related subfamily also evolved in subgroups in the evolutionary tree, and most of the MYB-related subfamilies appeared earlier than the differentiation time of monocotyledons and dicotyledons [26], which is similar to the results in this study.

In this study, we found that the members of Group3 exhibit a higher degree of conservatism compared to other members in the 3R-MYB subfamily (Figure 2). The number of members in wheat [47], peanut [48], and other species within 3R-MYB Group1 and 3R-MYB Group2 expanded with the multiplication of species chromosomes, while there was little change in the number of members for each species within 3R-MYB Group3. This suggests that 3R-MYB Group3 is more conservative than other members of the 3R-MYB family (3R-MYB Group4 is not considered here because there are too many species missing). We speculate that each group of MYB members under each subfamily may have their own special function. Interestingly, Assis studied the Drosophila genome and found that new functions almost entirely come from younger copies, while older copies have functions more similar to their ancestral genes [57]. In future studies, we will further explore the link between the degree of gene evolution and its functions, such as drought resistance.

### 4.2. Diversity of Expression Profiles of MYB Family among Species under Drought Stress

The root system serves as the primary absorptive organ of plants, facilitating the uptake of water from the soil [58,59]. The majority of a plant’s essential nutrients are absorbed from the soil through its roots, with only a small amount being taken in through above-ground tissues like leaves and stems [60,61]. In this study, the public transcriptome data of root from drought stress tests on five species found that the pathway of the MYB transcription factor family responding to drought stress shows species-specific diversity. Some members of the MYB family exhibit significant expression changes under drought stress, indicating their response to drought. However, not all of the DEGs are core genes involved in plant drought resistance. In future studies, we plan to investigate MYB transcription factors that play a central role in drought resistance in each species.

Quantitative RT-PCR results of the *MYB21* gene in *Arabidopsis thaliana* showed that drought stress increased the expression of *MYB21*, but phenotypically decreased the number and size of flowers and limited the development of stamens and pistils [62]. In maize, if water deficits decrease the water potential, it also leads to the inhibition of invertase activity and sugar transport, thereby impacting the development of ovaries and pollen [63]. In this study, members of the MYB family of gramineous wheat and rice were involved in the reproductive process related to flower organ development under drought stress, which was consistent with the results of previous studies.

In this study, we found that in four species, wheat, rice, soybean, and potato, DEGs were annotated to the recruitment function of transcriptional regulators, which indicates that these MYB transcription factors can co-regulate transcription processes with other cofactors. Experiments such as a yeast two-hybrid assay showed that NAC transcription factors and MYB transcription factors could synergistically regulate and inhibit the expression of *CrNCED5* and the biosynthesis of ABA [64]. A study found that the central factor of light signal transduction, MdHY5, could increase the accumulation of anthocyanin in apple by inhibiting *MdMYB16/308* with *MdMYBDL1*, revealing the co-regulation mechanism of HY5 and MYB transcription factors in anthocyanin accumulation [65]. Whether MYB families of different species have s different selection of co-regulatory partners under drought stress is a question worthy of future research.

In this study, it was found that DEGs were involved in the transcriptional regulation mediated by RNA polymerase II in three species: rice, soybean, and potato, under drought stress. A study found that under drought stress, RNA polymerase II would be recruited into the drought-induced genes and rapidly disappeared after rehydration [66]. In addition, the *ZmCHB101* gene, which affects maize gene expression profiles under osmotic stress, has been proven to interact with RNA in vivo and affect RNA-mediated transcription regulation [67].

There are also common features to the MYB family’s response to drought stress. This study found that DEGs were annotated to function in response to endogenous or exogenous stimuli (e.g., osmotic stress, hormones, acidic chemicals) in the MYB family of every investigated species. In this study, the expression of the *AtMYB60* gene in *Arabidopsis thaliana* was significantly up-regulated under drought stress (Figure 3). Several RT-PCR experiments found that the *AtMYB60* gene in *Arabidopsis thaliana* was up-regulated under ABA stimulation, strong osmotic stress, and a desiccation environment [68]. In this study, *GmMYB84 (Glyma.05G234600)* was up-regulated under drought stress (Supplementary Table S16). Moreover, Wan constructed a qRT-PCR assay to analyze the expression of *GmMYB84* gene, and the results showed that both drought treatment and ABA treatment significantly increased the expression of the gene within 24 h [69]. The analysis of MYB-related subfamily members in *Brassica napus* revealed a significant presence of *BnMYB*-related genes containing cis-acting elements that displayed responsiveness to various hormones, including abscisic acid and gibberellin [70]. Twenty-one ethylene-responsive element binding-factor-associated amphiphilic repression (EAR) motifs were identified in the potato MYB family [71]. The presence of these cis-acting elements and hormone-treated expression lineages further confirmed the finding that the MYB family can assist plants to mitigate drought effects by regulating changes in hormone levels.

In this study, DEGs in the MYB family of five species under drought stress was annotated into the circadian rhythm pathway. Studies on *Arabidopsis thaliana,* soybean, millet, etc., have shown that there is a relationship between a plant’s response to abiotic stresses (such as heat, cold, and drought) and its circadian rhythm or biological clock [72]. For example, the research on *Arabidopsis thaliana* shows that TOC1 (timing of CAB expression 1) can serve as a molecular switch to connect the biological clock with the plant response to drought [73]. This phenomenon has also been observed in other studies of soybeans: that drought stress could affect the gene expression of biological clock components (LCL1-, GmELF4-, and PRR-like) [74]. In this study, the *At-CCA1-16* gene of *Arabidopsis thaliana* (Supplementary Table S13), the *Traes.19F222799.7* gene of wheat (Supplementary Table S14), the *Os08g06110*.2 gene of rice (Supplementary Table S15), the *Glyma.03g2618004* gene of soybean (Supplementary Table S16), and the *Soltu.DM.03G019030.1* gene of potato (Supplementary Table S17) belong to a group of homologous genes with CCA1 and LHY, which are known as rhythm factors [37]. In future breeding work, researchers can take these common MYB genes as the core, with the species-specific genes as the auxiliary, to further improve the breeding efficiency of drought-tolerant varieties.

## 5. Conclusions

In this study, the number of MYB members in diploid species ranged from 258 (*Oryza sativa*) to 434 (*Linum usitatissimum*), while the number of MYB members in polyploid species ranged from 536 (*Nicotiana tabacum*) to 610 (*Glycine max*), revealing that the number of MYB family members had a linear relationship with the chromosome ploidy of species. A phylogenetic analysis showed that the evolutionary trend of the MYB family was that the differentiation among subfamilies preceded the differentiation of species, and the evolutionary trend of MYB subfamilies was diverse in different species. The pathways of MYB transcription factor families in response to drought stress showed species-specific diversity, and closely related species showed higher similarity. These results provide a theoretical basis and reference value for decoding the mechanism of cooperative drought resistance models among MYB transcription factor members and improving the efficiency of crop drought-resistance breeding.

## Figures and Tables

**Table 1:** Genome database information of selected species.

Species	Latin Name	Database
Grape	*Vitis vinifera* L.	Phytozome v13 (https://phytozome-next.jgi.doe.gov/)
Wheat	*Triticum aestivum* L.	
Rice	*Oryza sativa* L.	
Millet	*Setaria italica* L.	
Tomato	*Solanum lycopersicum* L.	
Potato	*Solanum tuberosum* L.	
Soybean	*Glycine max* L. Merr	
Barrel medic	*Medicago truncatula* Gaertn.	
Tobacco	*Nicotiana tabacum* L.	Sol Genomics Network (https://solgenomics.net/)
Cultivated peanut	*Arachis hypogaea* L.	Peanut Genome Resource (http://peanutgr.fafu.edu.cn/index.php)
Flax	*Linum usitatissimum* L.	Figshare (https://figshare.com/articles/dataset/Annotation_files_for_Longya-10_genome/13614311)
*Arabidopsis thaliana*	*Arabidopsis thaliana* L. Heynh	TAIR (https://www.arabidopsis.org/)

Note: genomic data of *Vitis vinifera*, *Triticum aestivum*, *Oryza sativa*, *Setaria italica*, *Solanum lycopersicum*, *Solanum tuberosum*, *Glycine max*, and *Medicago truncatula* were downloaded from the Phytozome v13 database. The Databases mentioned in the table were accessed from 1 January 2022 to 31 May 2023.

**Table 2:** Basic transcriptome information of the selected species.

Species	SRA Project Number	Drought Group Data Number	Control Group Data Number
*Arabidopsis thaliana*	PRJNA764209	SRR15931251	SRR15931262
		SRR15931253	SRR15931264
		SRR15931255	SRR15931266
*Triticum aestivum*	PRJEB44859	ERR6110517	ERR6110415
		ERR6110518	ERR6110416
		ERR6110471	ERR6110489
		ERR6110472	ERR6110490
		ERR6110473	ERR6110511
		ERR6110474	ERR6110512
*Oryza sativa*	PRJNA562309	SRR10814912	SRR10045073
		SRR10814913	SRR10045074
		SRR10814914	SRR10045075
*Glycine max*	PRJNA306380	SRR3033500	SRR3033503
		SRR3033501	SRR3033504
		SRR3033502	SRR3033505
*Solanum tuberosum*	PRJNA874012	SRR21209937	SRR21209941
		SRR21209920	SRR21209933
		SRR21209902	SRR21209915

Note: There were 3 replicates in the drought group and the control group, respectively, and each data number represented 1 replicate in the group.

**Table 3:** Number of MYB family members in each species.

Species	Total MYB	1R-MYB	2R-MYB	3R-MYB	4R-MYB
*Arabidopsis thaliana*	272	138	128	5	1
*Vitis vinifera*	269	135	128	6	0
*Triticum aestivum*	608	357	237	11	3
*Oryza sativa*	258	144	108	5	1 *
*Setaria italica*	285	157	122	4	2
*Solanum lycopersicum*	293	161	127	4	1
*Solanum tuberosum*	330	187	138	4	1
*Nicotiana tabacum*	536	326	197	11	2
*Glycine max*	610	306	294	8	2
*Arachis hypogaea*	541	292	238	9	2
*Medicago truncatula*	355	188	161	5	1
*Linum usitatissimum*	434	235	194	3	1 + 1 *

Note: * indicates that the gene has 5 conserved MYB domains.

## Data Availability

No new data were created.

## Supplementary Materials

The following supporting information can be downloaded at https://www.mdpi.com/article/10.3390/life14010141/s1: Tables S1–S12: Distribution of members of the MYB family; Tables S13–S17: DEGs of MYB transcription factor family under drought stress. Figures S1–S3: Conservative motifs of MYB family.
