# Supplementary material for: A Multi-Omics Analysis Revealed the Diversity of the MYB Transcription Factor Family’s Evolution and Drought Resistance Pathways
Source: Life (Basel). 2024 Jan 18;14(1):141. doi: 10.3390/life14010141 (PMC10820167; doi:10.3390/life14010141)
Supplement: Supplementary file 1 [file life-14-00141-s001.zip › life-2768657-supplementary.pdf]

Supplementary Table 1 Distribution of members of the *Arabidopsis thaliana* MYB family

Subfamily	Gene ID
1R-MYB	AT5G67580.1, AT5G61620.1, AT5G59570.1, AT5G59430.4, AT5G58900.1, AT5G58340.1, AT5G58080.1, AT5G56840.1, AT5G53200.1, AT5G52660.1, AT5G47390.1, AT5G45580.1, AT5G45420.1, AT5G44190.1, AT5G42630.2, AT5G37260.1, AT5G29000.4, AT5G23650.1, AT5G18240.2, AT5G17300.1, AT5G16560.1, AT5G08520.1, AT5G07210.1, AT5G06800.2, AT5G06110.1, AT5G05790.1, AT5G05090.1, AT5G04760.1, AT5G03780.1, AT5G02840.3, AT5G01380.1, AT5G01200.1, AT4G39250.1, AT4G39160.1, AT4G37180.1, AT4G36570.1, AT4G34430.3, AT4G31920.1, AT4G28610.1, AT4G18020.4, AT4G17695.1, AT4G16420.2, AT4G16110.1, AT4G13640.2, AT4G09450.1, AT4G04580.1, AT4G01280.1, AT4G01060.2, AT3G62670.1, AT3G60110.1, AT3G57980.1, AT3G53790.1, AT3G53440.1, AT3G52250.1, AT3G49850.1, AT3G46640.3, AT3G46590.3, AT3G25790.1, AT3G24880.1, AT3G24870.1, AT3G24120.1, AT3G21430.2, AT3G16857.1, AT3G16350.1, AT3G13040.1, AT3G12730.1, AT3G12560.1, AT3G11450.1, AT3G11280.1, AT3G10760.1, AT3G10590.1, AT3G10580.2, AT3G10113.1, AT3G09600.2, AT3G07740.2, AT3G04450.2, AT3G04030.2, AT2G47620.1, AT2G47210.1, AT2G46830.1, AT2G46410.1, AT2G44730.1, AT2G44430.1, AT2G42660.1, AT2G42150.1, AT2G40970.1, AT2G40260.1, AT2G38300.1, AT2G38090.1, AT2G37025.1, AT2G36960.2, AT2G33610.1, AT2G30432.1, AT2G30424.1, AT2G30420.1, AT2G25180.1, AT2G20570.1, AT2G20400.1, AT2G18328.1, AT2G13960.2, AT2G03500.1, AT2G02060.1, AT2G01760.1, AT2G01060.1, AT1G79430.2, AT1G75250.2, AT1G74840.2, AT1G72740.1, AT1G72650.2, AT1G71030.1, AT1G70000.1, AT1G69580.1, AT1G68670.1, AT1G67710.1, AT1G58220.1, AT1G49950.1, AT1G49560.1, AT1G49190.1, AT1G49010.1, AT1G32240.1, AT1G25550.1, AT1G21700.1, AT1G19510.1, AT1G19000.1, AT1G18960.1, AT1G18330.1, AT1G17520.1, AT1G17460.1, AT1G15720.1, AT1G14600.1, AT1G13300.1, AT1G09710.1, AT1G07540.1, AT1G06910.1, AT1G01520.1, AT1G01380.1, AT1G01150.1, AT1G01060.4
2R-MYB	AT3G06490.1, AT4G09460.1, AT1G66370.1, AT1G56160.1, AT4G37780.1, AT4G38620.1, AT1G74080.1, AT3G08500.1, AT5G10280.1, AT2G25230.1, AT2G23290.1, AT1G08810.1, AT1G06180.1, AT4G01680.3, AT1G57560.1, AT5G62320.1, AT1G79180.1, AT3G12820.1, AT3G01530.1, AT5G07700.1, AT5G54230.1, AT4G34990.1, AT5G56110.1, AT4G37260.1, AT5G23000.1, AT1G17950.1, AT4G12350.1, AT3G47600.1, AT5G12870.1, AT5G59780.3, AT4G33450.1, AT4G17785.1, AT5G62470.1, AT5G11050.1, AT1G35515.1, AT5G40330.1, AT3G28910.1, AT5G39700.1, AT5G17800.1, AT2G32460.2, AT1G48000.1, AT5G49330.1, AT4G28110.1, AT5G57620.1, AT3G12720.1, AT1G18710.1, AT3G24310.1, AT5G14340.1, AT3G02940.1, AT3G46130.1, AT5G06100.1, AT1G73410.1, AT2G02820.2, AT4G13480.1, AT1G22640.1, AT5G26660.1, AT3G50060.1, AT2G16720.1, AT3G27810.1, AT3G27785.1, AT2G39880.1, AT1G66380.1, AT3G49690.1, AT4G18770.1, AT5G61420.2, AT1G66230.1, AT5G14750.1, AT5G52600.1, AT3G61250.1, AT5G40430.1,

	AT1G09770.1, AT5G55020.1, AT4G25560.1, AT1G68320.1, AT5G49620.1, AT3G60460.1, AT3G30210.1, AT2G47460.1, AT3G29020.1, AT1G26780.1, AT3G53200.1, AT1G56650.1, AT1G25340.1, AT2G36890.1, AT5G65790.1, AT1G18570.1, AT3G27920.1, AT1G63910.1, AT5G65230.1, AT1G16490.1, AT4G22680.1, AT3G55730.1, AT5G60890.1, AT5G41020.1, AT1G34670.1, AT4G26930.1, AT3G13540.1, AT3G11440.1, AT5G52260.1, AT4G05100.1, AT1G69560.1, AT5G15310.2, AT3G23250.1, AT5G35550.1, AT5G07690.1, AT3G48920.1, AT1G74430.1, AT3G28470.1, AT3G13890.2, AT3G01140.1, AT2G31180.1, AT1G74650.1, AT5G40360.1, AT2G26950.1, AT2G47190.1, AT1G09540.1, AT5G40350.1, AT2G37630.1, AT2G26960.1, AT1G14350.1, AT5G16600.1, AT3G09230.1, AT3G62610.1, AT4G21440.1, AT5G58850.1, AT5G16770.1, AT5G67300.1, AT1G66390.1
3R-MYB	AT3G09370.1, AT4G00540.2, AT4G32730.1, AT5G02320.1, AT5G11510.2
4R-MYB	AT3G18100.2

Supplementary Table 2 Distribution of members of the Grape（*Vitis vinifera*）MYB family

Subfamily	Gene ID
1R-MYB	V216s0039g01762.1, V214s0006g00450.1, V214s0036g00460.1, V208s0007g01920.1,<br>V206s0004g06100.1, V217s0000g04130.1, V216s0039g01605.1, V210s0071g00660.1,<br>V204s0008g01820.1, V216s0039g01752.1, V202s0025g04220.1, V213s0019g03370.1,<br>V212s0134g00480.1, V206s0080g00790.1, V216s0039g01754.1, V219s0015g01170.1,<br>V218s0001g02210.1, V202s0033g00460.1, V213s0019g04010.3, V216s0039g01758.1,<br>V204s0079g00410.1, V207s0031g02280.1, V206s0004g00570.1, V215s0048g02410.1,<br>V219s0015g01165.1, V208s0040g03220.6, V203s0063g02410.1, V207s0031g02275.1,<br>V217s0000g07510.3, V218s0122g00670.2, V215s0046g02260.2, V202s0033g00370.1,<br>V204s0008g00900.2, V211s0016g02410.1, V218s0001g01170.1, V200s0194g00130.3,<br>V218s0001g02160.1, V216s0039g01765.1, V212s0057g00920.1, V201s0026g01050.1,<br>V216s0050g01180.2, V210s0116g00500.1, V201s0011g00790.1, V210s0116g01760.1,<br>V200s0299g00060.1, V209s0002g03540.1, V203s0038g04460.4, V209s0002g03610.1,<br>V211s0016g01890.1, V213s0156g00340.1, V218s0152g00016.1, V203s0091g01030.1,<br>V208s0007g07580.1, V208s0105g00370.1, V217s0000g00990.4, V213s0106g00370.1,<br>V211s0016g05650.1, V207s0005g04120.1, V206s0004g02520.6, V212s0059g02360.2,<br>V209s0054g01620.1, V207s0151g00480.1, V200s0475g00040.10, V208s0040g00900.1,<br>V208s0040g01640.1, V209s0002g01480.21, V214s0128g00860.1, V200s1311g00020.1,<br>V214s0036g01380.1, V202s0012g00630.1, V212s0028g00980.2, V212s0028g03100.2,<br>V205s0077g01480.1, V204s0023g01910.1, V202s0012g00570.1, V202s0033g00300.1,<br>V203s0017g02280.6, V206s0004g05120.1, V218s0001g07620.1, V201s0026g02550.1,<br>V208s0007g06310.3, V218s0001g12610.1, V216s0100g00420.1, V205s0029g00060.2,<br>V203s0063g02680.1, V215s0046g01130.1, V210s0003g01380.3, V202s0033g00440.1,<br>V212s0028g02080.3, V207s0005g01010.1, V207s0197g00060.1, V215s0046g01790.1,<br>V200s0475g00030.4, V209s0070g00405.1, V201s0011g05830.1, V203s0063g01060.1,<br>V201s0137g00050.1, V216s0050g02530.1, V211s0206g00060.2, V201s0010g02230.2,<br>V208s0058g00240.1, V204s0008g05900.4, V207s0005g02730.3, V219s0085g00890.2,<br>V201s0011g03110.1, V217s0000g10100.2, V213s0067g01500.1, V206s0004g07000.1,<br>V208s0040g00100.1, V208s0007g08160.10, V213s0156g00370.6, V208s0007g06180.1,<br>V200s1624g00010.1, V212s0057g01490.2, V208s0040g01460.1, V203s0063g02670.1,<br>V217s0000g10110.1, V211s0016g04600.3, V217s0000g06810.1, V218s0001g11390.2,<br>V207s0005g01910.4, V216s0100g00430.1, V216s0039g01900.3, V206s0004g08050.2,<br>V202s0012g01940.1, V204s0043g00340.7, V211s0016g04130.1, V206s0004g04940.3,<br>V201s0011g04220.1, V202s0087g00140.1, V218s0001g12530.2, V214s0108g01580.1,<br>V213s0019g01670.1, V207s0005g06680.1, V203s0063g02620.1
2R-MYB	V214s0006g01280.1, V207s0141g00100.1, V216s0013g01560.1, V216s0039g01920.1,<br>V204s0023g03710.1, V207s0005g01210.1, V205s0077g01360.1, V211s0078g00480.1,<br>V214s0219g00050.1, V215s0046g00170.1, V218s0117g00210.1, V211s0016g01320.1,<br>V214s0108g00830.1, V216s0039g01725.1, V214s0108g01010.1, V208s0007g01540.1,<br>V207s0005g01950.1, V217s0000g08480.1, V201s0026g01910.1, V218s0001g11170.1,<br>V209s0002g01400.1, V214s0066g01010.1, V218s0001g09850.1, V218s0001g05670.1,<br>V211s0016g02780.1, V205s0049g02260.1, V207s0129g01050.1, V201s0026g02770.1,<br>V219s0015g01280.1, V217s0000g02650.1, V201s0011g01190.1, V202s0033g00400.1,

	V217s0000g02660.1, V211s0016g05660.1, V216s0039g01761.1, V217s0000g01890.2, V204s0008g01870.1, V217s0000g09080.1, V207s0130g00040.1, V208s0056g00800.2, V216s0050g00050.1, V200s0203g00170.1, V214s0006g01340.1, V209s0002g01380.1, V214s0006g01290.1, V205s0077g00500.1, V211s0016g01300.1, V202s0033g00390.1, V212s0134g00490.1, V219s0090g00590.1, V205s0049g01020.1, V217s0000g08550.1, V202s0025g00320.1, V207s0005g03340.1, V217s0000g06410.1, V216s0039g01756.1, V216s0022g01210.1, V216s0050g00070.1, V213s0067g01360.1, V206s0004g04140.1, V213s0064g00960.1, V201s0011g04760.1, V208s0007g07230.1, V212s0059g00700.1, V202s0033g00450.1, V204s0008g00620.1, V203s0180g00210.1, V205s0020g01100.1, V219s0014g03820.1, V214s0083g00120.1, V216s0039g01710.1, V214s0066g01220.1, V216s0039g01764.1, V209s0002g01410.1, V216s0039g01740.1, V211s0052g01490.1, V202s0033g00430.1, V218s0001g08470.1, V212s0134g00570.1, V204s0008g01830.1, V217s0000g05400.1, V200s1352g00010.1, V206s0061g00470.1, V201s0011g01200.1, V200s0341g00050.1, V201s0127g00730.1, V208s0056g01190.2, V203s0038g02310.1, V214s0060g00240.1, V217s0000g06190.1, V207s0005g02480.1, V214s0083g01060.1, V206s0004g06280.1, V219s0085g00940.1, V215s0046g03190.1, V218s0117g00200.1, V208s0007g05030.1, V204s0044g01380.1, V205s0049g01010.1, V200s0203g00070.1, V200s1241g00010.1, V215s0021g02040.1, V213s0019g03200.1, V208s0007g00410.1, V213s0084g00530.1, V211s0016g01310.1, V201s0011g03730.1, V216s0013g01570.1, V214s0006g01620.1, V206s0009g02480.2, V217s0000g03560.1, V208s0007g04830.1, V202s0012g01650.1, V213s0067g01630.1, V202s0033g00410.1, V217s0000g02730.1, V211s0016g05690.1, V204s0008g01810.1, V201s0026g02600.1, V206s0004g02110.1, V216s0039g01763.1, V201s0010g00410.1, V214s0036g00500.1, V218s0001g15260.1, V204s0008g01840.1, V219s0085g00050.1, V214s0066g01090.1, V214s0066g02180.1
3R-MYB	V215s0048g02120.1, V211s0016g03750.2, V204s0008g03780.3, V204s0008g03790.5, V208s0007g00360.1, V216s0039g01750.1

Supplementary Table 3 Distribution of members of the Wheat（*Triticum aestivum*）MYB family

Subfamily	Gene ID
1R-MYB	Traes.216652F6B.1, Traes.839C94CA3.1, Traes.D9A4C1AB0.3, Traes.F7B9CBA99.1,<br>Traes.1787A8428.1, Traes.75F8A05CF.2, Traes.30D2DA7DA.1, Traes.F3F4A8B2A.1,<br>Traes.D1807A4E0.1, Traes.EEE59C2E7.1, Traes.304E62CCE.1, Traes.E250F49DB1.1,<br>Traes.5F686B67C.1, Traes.92EA1F290.4, Traes.4D25427CB.1, Traes.1074E21D6.1,<br>Traes.2EDC5C26F.1, Traes.98EA87DFA1.1, Traes.1EBB348CB.1, Traes.68BC65ED4.1,<br>Traes.E8C400686.1, Traes.2F2381640.1, Traes.562426A67.1, Traes.931C25F08.1,<br>Traes.BA181D2AD.1, Traes.26A958CDD.1, Traes.665B92F4E.2, Traes.1D415A4CF.3,<br>Traes.34B6145E5.6, Traes.300AB5CC9.11, Traes.3B8CB5283.1, Traes.282C4747C.4,<br>Traes.93815D497.1, Traes.D098A5241.1, Traes.8A9D9ACCB.1, Traes.1144FC823.1,<br>Traes.002FAE6E8.1, Traes.3041037F1.1, Traes.660616C44.2, Traes.31EED5C94.1,<br>Traes.3AB4EBE4F.1, Traes.BDFDE5BF1.1, Traes.A63427EE8.1, Traes.EDA7A1267.1,<br>Traes.060C24872.1, Traes.0F82AC8F0.1, Traes.458B5662C.1, Traes.E1F692526.1,<br>Traes.673985B1D.1, Traes.736D60ADD.1, Traes.BDDD76A8B.1, Traes.5CB4803CD.2,<br>Traes.8AF1A10AB.1, Traes.50C15318E.1, Traes.998EC9F74.1, Traes.310E46F15.7,<br>Traes.19F222799.7, Traes.77F235694.1, Traes.68851ADC8.1, Traes.270084801.1,<br>Traes.68FFE0FFA.1, Traes.2054A21EE.4, Traes.276CC5E27.5, Traes.9CC3EA7C9.3,<br>Traes.4DF357170.1, Traes.F18440729.1, Traes.73A93153A.1, Traes.71FC575B0.1,<br>Traes.6626D4A69.1, Traes.993477788.1, Traes.206AE5B76.1, Traes.7E8EBDE1E.4,<br>Traes.6506152C8.2, Traes.79F6F585D.5, Traes.F7D2C31DB.1, Traes.F809FA27D.1,<br>Traes.24DBD38A8.2, Traes.28559D303.1, Traes.C184D0525.3, Traes.9E45828B0.3,<br>Traes.6B4FA59C6.2, Traes.362F82EAD.30, Traes.30F715B53.13, Traes.DF23FE973.1,<br>Traes.F484B1A35.15, Traes.7E9C131F4.8, Traes.405FE0DBA.1, Traes.C13CD3ECB.1,<br>Traes.B45238368.2, Traes.F6CE72D8D.1, Traes.845402F4B.1, Traes.8398893D8.1,<br>Traes.B7F04D24D.1, Traes.297FBF3A3.1, Traes.C6BEE3896.1, Traes.61692C8F3.2,<br>Traes.794DAF12D.1, Traes.965E2F790.2, Traes.F1DB969A8.2, Traes.BB971D610.1,<br>Traes.572EF99CA.2, Traes.E65C48107.1, Traes.55A3F252C.1, Traes.6780F56DD.4,<br>Traes.AB55D9F4E.2, Traes.AE7851A5F.1, Traes.5D8EE6802.17, Traes.6EEB8D2B2.1,<br>Traes.475B27CF6.1, Traes.6A126F5F5.1, Traes.7034372AC.1, Traes.CF481F6AC.5,<br>Traes.12B53AB09.5, Traes.05774F3AC.7, Traes.03C96BECE.1, Traes.43EFBAAD5.1,<br>Traes.16279F989.27, Traes.32D8155C6.3, Traes.D06222817.1, Traes.73B7FB5C1.3,<br>Traes.F227310FD.1, Traes.8EA143F67.1, Traes.62FDC3C38.1, Traes.34669CA7B.4,<br>Traes.FB63C505B.5, Traes.C729D25F8.1, Traes.239CDF865.1, Traes.28B163E7C.2,<br>Traes.58DDB90E3.1, Traes.8417C5F8B.1, Traes.9A1395EC7.1, Traes.5869E3C31.1,<br>Traes.1B7CF183F.3, Traes.73BC102D3.3, Traes.C0F4A948C.3, Traes.2CFFBD60E.3,<br>Traes.7589A1385.3, Traes.30CF35BB3.1, Traes.8756ECB5A.8, Traes.0E0A747A1.1,<br>Traes.72D2B7C35.1, Traes.EAF038D23.3, Traes.EB206B8BC.3, Traes.3EE830A43.8,<br>Traes.72808D4CB.1, Traes.5E492444E.1, Traes.6F77CFB03.10, Traes.DEA065330.1,<br>Traes.8ACA5E563.2, Traes.FEA7D3672.1, Traes.BF6FD97DA.1, Traes.119609AE8.1,<br>Traes.9636192C8.1, Traes.FF8F7A939.1, Traes.D00AB8CFA.9, Traes.8D627C185.1,<br>Traes.F6047634A.1, Traes.0C01933E1.1, Traes.10172C13A.1, Traes.B30884E0E.1,<br>Traes.6A75A61AC.1, Traes.DA7413B04.1, Traes.2C27C34AF.4, Traes.4476835E0.1,<br>Traes.015B8CB08.1, Traes.17C974117.1, Traes.93E51B797.1, Traes.435A95A74.9,

	Traes.72ECC7747.1, Traes.2B0A44F87.1, Traes.9E9E5FD89.2, Traes.7E9E3A389.3, Traes.C9D25868F.1, Traes.ED6E0EE34.1, Traes.EB47672ED.5, Traes.A2C97A02D.5, Traes.99EC36CD4.2, Traes.20D189350.3, Traes.87EF37490.5, Traes.A631E1F0C.5, Traes.00D311B94.2, Traes.692FEF87A.7, Traes.B71F3F028.3, Traes.3C6511D69.1, Traes.7220D33B3.4, Traes.45417CBA9.6, Traes.0C6735099.1, Traes.DC5786149.5, Traes.C0A2B4F041.1, Traes.552D41920.6, Traes.BBAC725C3.2, Traes.991931CF9.2, Traes.E2629CBC1.1, Traes.61E35C455.1, Traes.8224C29A9.1, Traes.A33076690.1, Traes.3B4E00416.7, Traes.6F783E776.6, Traes.327FBC2CA.2, Traes.4B5FE030C.1, Traes.2F376689A.5, Traes.1FB74A6E1.3, Traes.5B5A264A8.1, Traes.1C6A8C773.2, Traes.17EF1FF01.3, Traes.9D2735F60.2, Traes.C5FFFD54A.1, Traes.DBE4E3146.1, Traes.090D0E08E.1, Traes.01E24503F.1, Traes.8781D3C6C.1, Traes.43D709979.1, Traes.D45376F32.5, Traes.D82CA7827.2, Traes.44456AE22.1, Traes.3640B95AB.2, Traes.2527CA8BF.4, Traes.D9F27151E.1, Traes.8D3A3687D.1, Traes.09DD5A1DC.1, Traes.EFD6D48C0.1, Traes.1B12D7856.1, Traes.C32B01619.2, Traes.C32B016191.2, Traes.AE0BEF1BE.6, Traes.9C1318B2F.1, Traes.C73E7E6FB.1, Traes.A44464337.1, Traes.6486B493A.1, Traes.941E7DE4D.1, Traes.0862C6644.1, Traes.1318B59D3.3, Traes.A28533902.1, Traes.156E53683.1, Traes.B26E341BE.3, Traes.EED669F6A.1, Traes.EED669F6A1.1, Traes.AC25F01A6.1, Traes.22DE624A5.3, Traes.DECB8080D.1, Traes.891566958.1, Traes.05E08500B.3, Traes.50D6A231C.1, Traes.16EECD38D.9, Traes.0E9BE0271.1, Traes.31CD3BB2E.8, Traes.481D51B66.2, Traes.E94233A40.4, Traes.ED205638C.1, Traes.F20FD5D20.2, Traes.86AF9114B.1, Traes.25253EF6C.6, Traes.7F92C0CB9.1, Traes.476AB27D8.5, Traes.3FC31F9ED.1, Traes.F7F1834AA.1, Traes.138F3DBE5.1, Traes.36467B9A4.1, Traes.B50BEBC7E.4, Traes.F09C7CA19.1, Traes.A42E71A00.2, Traes.2B62A6D74.1, Traes.D9A29948B.1, Traes.EF4A57213.2, Traes.7AA5EFD05.6, Traes.264CE0C2E.1, Traes.8DEB9794C.1, Traes.9DC9A9192.5, Traes.790C095EE.1, Traes.D1C9CE275.1, Traes.A84ABE6EF.1, Traes.503B57D77.1, Traes.C35CABF69.1, Traes.0190EDEFD.1, Traes.64EAA3388.1, Traes.865644EC4.7, Traes.FA727026F.2, Traes.4D42B475B.2, Traes.08603B02C.2, Traes.82133897D.1, Traes.EED456A17.2, Traes.C9EA93E10.1, Traes.13C2391DC.2, Traes.D98C7CE92.1, Traes.5D47DC487.1, Traes.A20157EC5.1, Traes.92939B028.1, Traes.999E12D5D.1, Traes.B2F166A31.1, Traes.06889C77D.2, Traes.1EDDDC063.6, Traes.E82B2B6AC.1, Traes.39E5EF501.2, Traes.4EFA77404.1, Traes.B2256B77F.3, Traes.EC75E7941.8, Traes.17C8D2707.2, Traes.2125E90F3.6, Traes.10A8CAB27.1, Traes.C7C1FFE0C.1, Traes.EFE6EFEDF.1, Traes.CFCC2012F.1, Traes.2EDF05396.4, Traes.DB1546316.3, Traes.4B9F0CBBF.1, Traes.808AD9543.2, Traes.976FAF094.2, Traes.4E8553041.2, Traes.9C9D677D4.1, Traes.07B7BEEC31.1, Traes.5A4046554.5, Traes.7773A9BFB.7, Traes.1360F98E7.1, Traes.1AE458202.2, Traes.82E82F8F9.2, Traes.C8C8B8DA9.1, Traes.1141C575C.4, Traes.8E8190A0F.4, Traes.6E13A3C8C.2, Traes.3C04C3D2F.7, Traes.76515C538.1, Traes.FD9C65DC01.1, Traes.9E032AF27.1, Traes.8719DBF92.1, Traes.FC1A52B36.1, Traes.95CDF7628.1, Traes.98A6A7CC2.1, Traes.3C62DC707.1, Traes.660FDCED9.6, Traes.0535EFFBE.1, Traes.19B90732F.1, Traes.D238844BB.1, Traes.4E68C2154.5, Traes.0644187E5.1, Traes.A3420082C.1, Traes.70CCAB0EF.3, Traes.A92573EF7.7, Traes.546DFB91B.4, Traes.EEC3977E0.3, Traes.3BC4F011C.1, Traes.F0A1D1118.2, Traes.4490ED4C6.1, Traes.B5AED6ABA.7, Traes.2D464CB3E.1, Traes.FDBBC5E8F.2, Traes.653FEEC33.1, Traes.D50CC6A1D.1, Traes.D50CC6A1D1.1,

	Traes.C74DAC3CE.1, Traes.38B29E4C9.2, Traes.DA7C4AFF6.11, Traes.DEE69C6AC.1, Traes.32F10AE2C.1, Traes.BF90D232B.1, Traes.E74A51BAE.1, Traes.169CE67D8.1, Traes.BB4298457.2
2R-MYB	Traes.DFCF4A15A.1, Traes.B181F1FD2.1, Traes.F1B450FA4.4, Traes.50335C9A8.1, Traes.E635B0A281.1, Traes.3F5D36630.1, Traes.0D6CD031E.3, Traes.8E4A831B6.1, Traes.9BF07972C.2, Traes.7D519210E.1, Traes.90B64301A.5, Traes.02507996C.1, Traes.429583AEC.1, Traes.189002AB8.1, Traes.56F30392D.1, Traes.5E49D47AF.1, Traes.FE8B8ABC4.1, Traes.49959692B1.1, Traes.0ECE7A421.1, Traes.2CD01D459.1, Traes.2EE7C2628.10, Traes.A8AF980F3.1, Traes.0B2C5ADF6.3, Traes.ADB23D42F.1, Traes.962A9D448.1, Traes.557E7700B.4, Traes.7E2D1D34C.2, Traes.711044AD5.1, Traes.605C25CDF.2, Traes.29EF04226.1, Traes.C4F9BCFA2.1, Traes.CA02DC5B2.1, Traes.D41CB81EA.1, Traes.14C563681.1, Traes.A337F362A.1, Traes.46D39C394.5, Traes.92CBBE968.1, Traes.8F05AE571.3, Traes.C964675DE.1, Traes.25C93FD291.1, Traes.57F246ADF.1, Traes.5BD0D4BD1.1, Traes.D28CCE3B8.1, Traes.AF9357B4C.2, Traes.A79A68739.1, Traes.95A52A87F.1, Traes.8954A60B6.1, Traes.901181FDD.4, Traes.61B920833.1, Traes.5EE86C5BC.1, Traes.604FE7639.1, Traes.8BF29D46C.1, Traes.36CD3229A.3, Traes.CC5803BED.3, Traes.8008803A5.1, Traes.1FA87027B.1, Traes.934488D20.1, Traes.5A0300CE0.1, Traes.FD8C2F815.3, Traes.D05736670.7, Traes.CC403D182.2, Traes.CFEE687CF.1, Traes.1D036F8B5.1, Traes.152A7186A.1, Traes.FCDEFA244.1, Traes.2CB91C54A.1, Traes.57D51731D.3, Traes.3834B629A.1, Traes.20EC9096A.1, Traes.8F3BD4450.1, Traes.AA6863F0D.1, Traes.E5A9546C9.1, Traes.1A93B0C96.1, Traes.5100A9609.1, Traes.926486EE0.1, Traes.6253482BD.5, Traes.7C1FD879C.2, Traes.D1B5EA05F.1, Traes.545A5716E.1, Traes.7755E4A8A.1, Traes.04D939077.1, Traes.91299CB97.1, Traes.46F83C41D.1, Traes.FAF2AE9FB.1, Traes.486D0D865.1, Traes.BDECC07BD.21, Traes.AD4395F8F.1, Traes.E3BF20F0D.7, Traes.F0688BFDE.1, Traes.F83CB32DD.3, Traes.2A5B4C4C4.4, Traes.532725AD5.1, Traes.731C703DE.6, Traes.41B71F83C.1, Traes.30B272DDD.1, Traes.5BAD8947E.1, Traes.7183519F8.1, Traes.E5B3B79F7.1, Traes.3431FB444.1, Traes.FBC0ED0EE.2, Traes.1CD39D4D0.2, Traes.5B57EF9FC.3, Traes.B7FFC33B1.3, Traes.855A1170C.3, Traes.FDFCBA57D1.1, Traes.C13753B31.2, Traes.C664E8E1E.1, Traes.8ABCB84A7.2, Traes.C1D4586B1.2, Traes.90A30CBB4.1, Traes.3C4ECAB6D.7, Traes.B2C9F123A.1, Traes.469ED3135.1, Traes.30D8E6BE2.3, Traes.FF1508B19.1, Traes.E1E541767.2, Traes.CF98E922B.2, Traes.41CBE9959.2, Traes.5A2DB8F78.1, Traes.91CCCF6DB1.1, Traes.7A841CAD2.1, Traes.A0676BAEA.1, Traes.A0EC5D808.2, Traes.3883DC244.1, Traes.0AD4A7A87.1, Traes.2714E3604.1, Traes.47E2D90AC.1, Traes.F49830790.1, Traes.54E6F0446.1, Traes.DACD935B2.1, Traes.1DB727AD1.1, Traes.FA7059723.1, Traes.B41FDD6AA.4, Traes.2BA02CAA9.1, Traes.25726E5E7.1, Traes.1161E1D2E.4, Traes.CD74215DB.1, Traes.499F64524.1, Traes.2D4100475.1, Traes.918A8F357.1, Traes.0A21FB42C.1, Traes.F6E1A4F88.1, Traes.8BAAC240D.1, Traes.7C031019E.1, Traes.AAFB56987.2, Traes.D764DEA51.3, Traes.62CC650D3.1, Traes.4D9406708.1, Traes.BB730675A.1, Traes.D85C0EF26.6, Traes.AED68C680.6, Traes.4EFBA88E4.1, Traes.1868E2A6C.9, Traes.E4294BADC.1, Traes.35241DE8D.1, Traes.6BD0572DF.1, Traes.7BFAC49C2.5, Traes.560CCE975.1, Traes.1521D205A.1, Traes.A848F629F.2, Traes.E8D2BD66E.1, Traes.825A1B8BD.1, Traes.36AF74187.2, Traes.25C93FD29.1, Traes.3D67D3661.2, Traes.5562B97F7.2, Traes.52227091B.1, Traes.E2F8D46CE.4,

	Traes.343F2D219.1, Traes.759D8DDBB.2, Traes.59260A671.2, Traes.50CECDF43.1, Traes.361925B62.1, Traes.54D562BC2.1, Traes.7CEC6A8D7.1, Traes.5690C215A.5, Traes.25FD0FFB4.1, Traes.097BADCFE.5, Traes.D10CBA40D.1, Traes.230A3A2D0.18, Traes.1EF547639.1, Traes.E1D3D8DAA1.1, Traes.54D11D5E7.1, Traes.A03AB7608.2, Traes.9023F3504.4, Traes.82024A6C1.1, Traes.5F17B1022.2, Traes.28010F812.1, Traes.7E91DE546.3, Traes.49959692B.1, Traes.88BBF0441.1, Traes.03F3475CD.1, Traes.A014E9069.1, Traes.89BA7115C.1, Traes.B68E59AAB.1, Traes.3569AEDFB.1, Traes.9430AAD52.12, Traes.91CCCF6DB.1, Traes.B01AE830F.1, Traes.2D2135570.1, Traes.C843D1851.1, Traes.CE61B9510.1, Traes.79F1B50DF.1, Traes.7800B74E3.3, Traes.47E2D90AC1.1, Traes.8DF2537B8.1, Traes.67FD04DA0.1, Traes.FD6DBA41D.1, Traes.DE2C70E4C.2, Traes.B44ECE28C.1, Traes.DAA2104F5.2, Traes.25320ED72.1, Traes.B8E8A2F55.3, Traes.8B52D54FD.2, Traes.E27ECBC6E.2, Traes.5EEED86AD.6, Traes.D1C03C165.1, Traes.FCDEB9A8B.1, Traes.FDFCBA57D.1, Traes.1D7D3D0DE.1, Traes.4EE7DFCCD.1, Traes.0501BC320.5, Traes.4C9807337.1, Traes.967152326.1, Traes.912473A86.2, Traes.C7FD90C13.1, Traes.632EBAD09.1, Traes.65172E704.3, Traes.1EE692FDC.1, Traes.87924B626.5, Traes.61B30E3E4.1, Traes.0BCF482E8.1, Traes.2A12B5E70.1, Traes.40E835A94.3, Traes.40FA27AE7.1, Traes.34A0848F2.1, Traes.D39684C41.2
3R-MYB	Traes.B26A733D4.1, Traes.09025DA2E.2, Traes.51EBF8930.2, Traes.B6ADF4159.1, Traes.403DBC53C.1, Traes.B594FC28C.7, Traes.386795528.1, Traes.ABF465DDF.1, Traes.6BBC889A1.1, Traes.C25B5DDB4.1, Traes.61D017632.1
4R-MYB	Traes.78AD0AF8E.5, Traes.4F38323F3.1, Traes.9B80DEC84.1

Supplementary Table 4 Distribution of members of the Rice（*Oryza sativa*）MYB family

Subfamily	Gene ID				
1R-MYB	Os01g50110.1,	Os06g10350.1,	Os01g04930.1,	Os05g37730.1,	Os05g37060.1,
	Os01g63460.1,	Os07g30130.1,	Os06g14010.1,	Os01g64360.1,	Os04g58020.1,
	Os01g11200.1,	Os05g02420.2,	Os03g62100.1,	Os06g19980.1,	Os01g34060.1,
	Os06g51260.1,	Os03g19630.1,	Os01g07430.1,	Os04g49450.1,	Os01g06320.1,
	Os02g30700.1,	Os06g14710.1,	Os02g46030.1,	Os10g41260.1,	Os03g53960.2,
	Os02g10060.1,	Os05g03550.1,	Os03g51220.1,	Os03g25304.1,	Os08g06110.2,
	Os01g09640.1,	Os04g30890.1,	Os11g08080.2,	Os07g25150.1,	Os05g10690.1,
	Os08g05510.2,	Os05g37050.1,	Os08g04840.1,	Os08g06240.1,	Os02g45670.1,
	Os10g41200.3,	Os12g07730.1,	Os05g07010.2,	Os06g45840.2,	Os01g41900.2,
	Os01g09280.1,	Os06g01670.1,	Os05g51160.2,	Os04g40420.1,	Os01g12700.1,
	Os01g40670.2,	Os03g20900.1,	Os01g51154.3,	Os07g49530.1,	Os01g62660.1,
	Os07g25710.4,	Os06g07640.1,	Os12g41920.1,	Os01g74020.1,	Os01g43180.1,
	Os07g48596.1,	Os07g26150.1,	Os03g62379.1,	Os09g31454.1,	Os04g01970.1,
	Os05g40960.1,	Os06g24070.1,	Os03g21240.1,	Os02g47190.1,	Os06g07650.1,
	Os10g39550.1,	Os08g39980.1,	Os03g13790.1,	Os03g03760.1,	Os05g34110.1,
	Os01g43230.3,	Os09g12770.2,	Os01g13740.1,	Os04g56990.2,	Os04g27410.1,
	Os02g04640.1,	Os10g30690.1,	Os08g25820.1,	Os02g43300.1,	Os01g09760.1,
	Os03g63890.1,	Os11g01480.1,	Os10g30719.1,	Os01g67770.1,	Os12g01490.1,
	Os09g12750.1,	Os03g12350.1,	Os06g40710.1,	Os06g35140.1,	Os02g55320.1,
	Os06g49040.1,	Os02g14490.1,	Os06g08440.1,	Os06g07740.1,	Os08g25799.1,
	Os05g49240.1,	Os02g34630.2,	Os02g07770.1,	Os04g47750.1,	Os02g07170.1,
	Os03g56234.1,	Os02g53670.2,	Os02g08500.1,	Os01g47370.1,	Os12g33950.1,
	Os06g08290.1,	Os06g45410.1,	Os03g55760.1,	Os05g41240.1,	Os06g07700.1,
	Os06g45890.1,	Os01g44370.1,	Os03g14810.1,	Os02g46940.1,	Os12g39640.1,
	Os08g06370.1,	Os06g43910.1,	Os03g55590.1,	Os08g08000.1,	Os10g32600.1,
	Os02g22020.1,	Os08g33050.1,	Os07g02800.2,	Os08g33750.1,	Os01g08160.1,
	Os04g47890.1,	Os09g23200.1,	Os04g45940.1,	Os02g57270.1,	Os02g45080.1,
	Os07g04430.1,	Os03g43800.2,	Os08g08130.1,	Os03g45194.1,	Os05g37040.1,
	Os01g36850.1, Os09g14880.1, Os05g50350.1, Os02g47744.1				
2R-MYB	Os11g45740.1,	Os02g17190.1,	Os03g20090.1,	Os08g37970.1,	Os11g35390.1,
	Os02g36890.1,	Os02g02370.1,	Os07g43420.1,	Os02g49250.1,	Os06g06740.1,
	Os03g26130.1,	Os05g41166.1,	Os07g14110.1,	Os01g19330.1,	Os12g38400.2,
	Os01g63160.1,	Os01g36460.1,	Os12g07640.1,	Os07g43580.1,	Os02g09480.1,
	Os12g03150.1,	Os08g05520.1,	Os02g42870.1,	Os01g63680.1,	Os04g46384.1,
	Os02g54520.1,	Os11g10130.1,	Os04g50770.1,	Os01g03720.1,	Os03g04900.1,
	Os01g50720.1,	Os08g43550.1,	Os07g44090.3,	Os01g52410.1,	Os05g35500.1,
	Os06g43090.1,	Os03g25550.1,	Os01g74410.1,	Os03g38210.1,	Os06g11780.1,
	Os05g46610.1,	Os03g13310.1,	Os07g48870.1,	Os02g51799.1,	Os01g19970.1,
	Os07g12130.1,	Os02g41510.1,	Os03g56090.1,	Os05g48010.1,	Os08g33150.1,
	Os05g28320.1,	Os04g28090.1,	Os09g36730.1,	Os02g49986.1,	Os03g27090.1,
	Os08g33940.1,	Os09g26170.1,	Os10g35660.1,	Os03g19120.1,	Os01g09590.1,
	Os04g39470.1,	Os03g18480.1,	Os04g43680.1,	Os04g45060.1,	Os03g29614.1,

	Os01g16810.1, Os07g31470.1, Os04g42950.1, Os01g74590.1, Os12g37690.1, Os11g47460.1, Os01g51260.1, Os09g23620.1, Os08g34960.1, Os07g37210.1, Os04g50680.1, Os06g02250.1, Os05g04210.1, Os08g15020.1, Os12g33070.1, Os05g49310.1, Os02g46780.1, Os01g18240.1, Os08g33800.1, Os08g33660.1, Os04g38740.1, Os10g33810.1, Os09g24800.1, Os12g07610.1, Os07g25370.1, Os12g37970.1, Os06g14670.1, Os06g46560.1, Os06g40330.1, Os02g42850.2, Os06g14700.1, Os11g03440.1, Os03g51110.1, Os09g36250.1, Os05g04820.1, Os01g45090.1, Os09g01960.1, Os01g65370.1, Os01g07450.1, Os04g45020.1, Os02g40530.1, Os01g49160.1, Os01g59660.3
3R-MYB	Os05g38460.1, Os12g13570.1, Os10g20990.1, Os01g12860.1, Os01g62410.1
5R-MYB	Os07g04700.6

Supplementary Table 5 Distribution of members of the Millet（*Setaria italica*）MYB family

Subfamily	Gene ID
1R-MYB	Seita.9G171200.1, Seita.5G385300.1, Seita.1G077900.1, Seita.3G222600.1,<br>Seita.5G098300.1, Seita.2G140900.1, Seita.3G016200.1, Seita.3G225500.1,<br>Seita.5G391300.1, Seita.5G183700.1, Seita.5G044000.1, Seita.2G079500.1,<br>Seita.4G288100.1, Seita.7G212900.1, Seita.1G275400.1, Seita.3G325900.1,<br>Seita.3G048100.3, Seita.6G054000.1, Seita.1G051900.1, Seita.6G055700.1,<br>Seita.9G112600.2, Seita.8G060100.1, Seita.6G148300.1, Seita.9G319600.1,<br>Seita.5G135900.1, Seita.6G063100.2, Seita.9G090600.1, Seita.2G103800.1,<br>Seita.7G065700.1, Seita.1G272700.5, Seita.3G111800.1, Seita.6G065500.1,<br>Seita.2G302300.1, Seita.9G321800.1, Seita.2G104000.1, Seita.4G056600.1,<br>Seita.5G110700.1, Seita.4G004600.2, Seita.7G284300.2, Seita.4G266800.1,<br>Seita.4G214200.1, Seita.5G216800.1, Seita.4G118600.1, Seita.3G058100.1,<br>Seita.3G124200.1, Seita.2G174300.1, Seita.4G218000.2, Seita.5G289500.1,<br>Seita.2G436900.1, Seita.5G209300.1, Seita.7G138800.1, Seita.2G118400.1,<br>Seita.9G419000.1, Seita.9G113900.2, Seita.5G379100.1, Seita.9G113800.1,<br>Seita.7G163000.1, Seita.5G114000.1, Seita.3G199300.1, Seita.5G003100.1,<br>Seita.9G416300.1, Seita.7G223500.1, Seita.5G133800.1, Seita.5G468100.1,<br>Seita.1G286400.1, Seita.4G156200.1, Seita.3G354900.1, Seita.9G238700.1,<br>Seita.9G554300.1, Seita.3G242400.1, Seita.9G238800.1, Seita.9G473800.1,<br>Seita.5G238400.1, Seita.6G206100.1, Seita.3G029000.1, Seita.5G136900.1,<br>Seita.9G158300.2, Seita.2G168900.1, Seita.4G132300.1, Seita.2G254700.1,<br>Seita.4G266400.1, Seita.4G185800.1, Seita.9G467400.1, Seita.6G121500.1,<br>Seita.3G058200.1, Seita.7G332300.1, Seita.4G265300.1, Seita.1G190100.1,<br>Seita.5G419200.2, Seita.2G444900.2, Seita.1G169900.1, Seita.5G259700.1,<br>Seita.5G260200.1, Seita.5G452200.1, Seita.8G019800.1, Seita.9G340000.1,<br>Seita.9G485100.1, Seita.4G270600.1, Seita.2G168800.2, Seita.1G351300.1,<br>Seita.5G238500.1, Seita.1G021500.1, Seita.4G050700.1, Seita.4G175600.1,<br>Seita.1G142100.2, Seita.6G120200.1, Seita.4G085600.1, Seita.1G338400.2,<br>Seita.1G069400.1, Seita.9G308900.1, Seita.9G080000.1, Seita.1G092500.1,<br>Seita.4G273600.2, Seita.1G061200.1, Seita.9G078300.2, Seita.8G160000.2,<br>Seita.9G231000.1, Seita.3G143100.1, Seita.7G201600.2, Seita.1G283000.1,<br>Seita.3G049300.1, Seita.3G134100.1, Seita.7G029600.1, Seita.3G373000.1,<br>Seita.6G053400.2, Seita.3G228700.1, Seita.9G493400.2, Seita.9G165800.2,<br>Seita.9G040100.1, Seita.4G215900.1, Seita.5G259200.1, Seita.9G536000.1,<br>Seita.6G152600.1, Seita.3G163600.1, Seita.3G370000.1, Seita.1G146600.1,<br>Seita.9G361100.1, Seita.5G124700.1, Seita.7G135900.1, Seita.3G133900.1,<br>Seita.4G157600.1, Seita.2G197100.1, Seita.1G268700.2, Seita.4G151500.1,<br>Seita.2G013500.1, Seita.5G024000.4, Seita.4G198600.1, Seita.4G198500.1,<br>Seita.9G318500.1, Seita.9G047800.1, Seita.5G468700.2, Seita.7G272200.1,<br>Seita.3G195600.1, Seita.7G185800.1, Seita.3G121600.2, Seita.4G148800.1,<br>Seita.1G184800.1
2R-MYB	Seita.6G168800.1, Seita.1G250900.1, Seita.3G055800.1, Seita.3G154700.1,<br>Seita.5G355300.1, Seita.6G158800.1, Seita.7G179800.1, Seita.7G284800.1,

	Seita.3G161500.1, Seita.2G199900.1, Seita.7G320700.2, Seita.7G284900.1, Seita.4G173600.1, Seita.2G199800.1, Seita.8G057800.1, Seita.6G211500.1, Seita.7G279500.1, Seita.7G030000.1, Seita.6G160100.1, Seita.2G200200.1, Seita.1G007300.3, Seita.2G438400.1, Seita.3G142100.1, Seita.5G192400.1, Seita.2G351400.1, Seita.5G043900.1, Seita.6G238400.1, Seita.9G181600.1, Seita.5G043800.1, Seita.3G333800.1, Seita.9G425100.1, Seita.9G054400.1, Seita.9G204400.1, Seita.5G286200.1, Seita.2G400600.1, Seita.4G205800.1, Seita.3G232500.1, Seita.4G261400.1, Seita.9G160300.1, Seita.2G320600.1, Seita.1G117000.1, Seita.6G154200.1, Seita.2G204300.1, Seita.9G544000.1, Seita.7G161400.1, Seita.8G101000.1, Seita.2G286400.1, Seita.2G399900.2, Seita.5G239400.1, Seita.7G219700.1, Seita.9G384800.1, Seita.4G115200.1, Seita.6G239700.1, Seita.7G285100.1, Seita.7G057500.1, Seita.1G324800.1, Seita.1G209100.1, Seita.5G274200.1, Seita.7G307500.1, Seita.3G350800.1, Seita.2G289900.1, Seita.3G408700.1, Seita.3G331100.1, Seita.8G237700.1, Seita.9G368500.1, Seita.3G062200.1, Seita.5G239300.1, Seita.1G344700.1, Seita.6G063000.1, Seita.2G404100.1, Seita.5G290600.2, Seita.1G250600.1, Seita.6G190300.1, Seita.5G118900.1, Seita.2G138700.1, Seita.1G120500.1, Seita.4G008000.1, Seita.8G153200.1, Seita.7G167300.1, Seita.7G179600.1, Seita.9G074800.1, Seita.7G132600.1, Seita.5G283200.1, Seita.5G301200.1, Seita.5G034500.1, Seita.2G213200.1, Seita.5G386900.1, Seita.1G374700.1, Seita.7G220500.1, Seita.3G079100.1, Seita.9G113200.1, Seita.4G221900.1, Seita.2G200100.1, Seita.7G188300.1, Seita.9G476600.1, Seita.9G390800.1, Seita.4G086300.1, Seita.1G281500.1, Seita.7G125200.1, Seita.6G097100.1, Seita.9G432200.1, Seita.5G087200.1, Seita.5G382600.1, Seita.3G051800.1, Seita.5G025200.1, Seita.5G119000.1, Seita.1G239400.1, Seita.1G309600.1, Seita.5G028900.1, Seita.9G222100.1, Seita.3G361000.1, Seita.4G091600.1, Seita.3G062400.1, Seita.6G158000.1, Seita.3G278400.1, Seita.3G223400.1, Seita.3G402900.1, Seita.5G398000.1, Seita.5G135700.1, Seita.1G236400.1, Seita.8G244100.1, Seita.8G006900.1
3R-MYB	Seita.3G217600.1, Seita.5G008900.1, Seita.5G377100.1, Seita.3G079800.1
4R-MYB	Seita.1G007400.3, Seita.2G028600.1

Supplementary Table 6 Distribution of members of the Tomato（*Solanum lycopersicum*）MYB family

Subfamily	Gene ID			
1R-MYB	Solyc05g013430.1.1,	Solyc06g033870.1.1,	Solyc11g006720.2.1,	Solyc09g014250.3.1,
	Solyc03g096350.3.1,	Solyc06g076770.3.1,	Solyc05g055240.3.1,	Solyc10g076820.2.1,
	Solyc02g086690.3.1,	Solyc05g052610.3.1,	Solyc04g008870.3.1,	Solyc11g071500.3.1,
	Solyc03g119740.3.1,	Solyc04g050080.1.1,	Solyc12g019840.1.1,	Solyc08g007300.3.1,
	Solyc04g049120.4.1,	Solyc01g009650.1.1,	Solyc12g019810.1.1,	Solyc12g088610.1.1,
	Solyc02g082390.1.1,	Solyc06g075660.4.1,	Solyc06g005310.3.1,	Solyc02g036370.3.1,
	Solyc03g098320.4.1,	Solyc12g005890.3.1,	Solyc10g005080.3.1,	Solyc03g113530.3.1,
	Solyc05g054200.4.1,	Solyc04g082760.3.1,	Solyc12g008800.3.1,	Solyc10g084370.3.1,
	Solyc06g009430.3.1,	Solyc01g109510.3.1,	Solyc09g007580.3.1,	Solyc07g047610.3.1,
	Solyc07g026680.2.1,	Solyc11g050730.1.1,	Solyc04g005100.3.1,	Solyc08g065380.3.1,
	Solyc10g019150.1.1,	Solyc01g095030.3.1,	Solyc03g113620.3.1,	Solyc06g071230.3.1,
	Solyc11g013560.1.1,	Solyc11g013620.1.1,	Solyc01g096700.4.1,	Solyc08g078340.3.1,
	Solyc06g060120.3.1,	Solyc05g013420.1.1,	Solyc12g044610.2.1,	Solyc06g036300.4.1,
	Solyc02g064630.4.1,	Solyc02g082375.1.1,	Solyc09g007570.3.1,	Solyc01g106940.4.1,
	Solyc07g040920.2.1,	Solyc09g072830.4.1,	Solyc04g050070.3.1,	Solyc11g069850.3.1,
	Solyc10g085620.2.1,	Solyc01g065540.3.1,	Solyc06g008200.3.1,	Solyc06g034030.3.1,
	Solyc06g082510.3.1,	Solyc09g007990.3.1,	Solyc10g080960.1.1,	Solyc01g073950.4.1,
	Solyc05g054390.4.1,	Solyc10g083340.3.1,	Solyc06g005680.4.1,	Solyc11g022470.2.1,
	Solyc12g098370.2.1,	Solyc06g051060.4.1,	Solyc09g005030.3.1,	Solyc08g077230.3.1,
	Solyc11g067280.2.1,	Solyc10g078310.2.1,	Solyc05g055940.3.1,	Solyc06g066180.4.1,
	Solyc02g085145.1.1,	Solyc12g019900.2.1,	Solyc06g076350.3.1,	Solyc07g045000.4.1,
	Solyc11g044320.2.1,	Solyc03g121840.3.1,	Solyc01g095640.2.1,	Solyc04g050100.1.1,
	Solyc07g053630.4.1,	Solyc06g061030.3.1,	Solyc12g099380.3.1,	Solyc03g121990.4.1,
	Solyc11g072330.2.1,	Solyc09g091880.4.1,	Solyc04g076360.4.1,	Solyc08g076010.4.1,
	Solyc09g008100.4.1,	Solyc04g008050.4.1,	Solyc09g005370.1.1,	Solyc05g014260.3.1,
	Solyc12g087970.2.1,	Solyc04g008480.2.1,	Solyc01g108300.3.1,	Solyc10g080920.2.1,
	Solyc10g078720.2.1,	Solyc05g007890.4.1,	Solyc03g006150.2.1,	Solyc12g017370.3.1,
	Solyc10g076460.2.1,	Solyc02g076670.3.1,	Solyc07g052490.4.1,	Solyc01g079210.3.1,
	Solyc04g015290.3.1,	Solyc05g009720.3.1,	Solyc02g090400.4.1,	Solyc10g080300.1.1,
	Solyc12g010330.2.1,	Solyc06g069560.3.1,	Solyc12g089170.3.1,	Solyc04g080500.3.1,
	Solyc12g006280.3.1,	Solyc10g052470.1.1,	Solyc01g067560.3.1,	Solyc03g115440.3.1,
	Solyc12g089190.1.1,	Solyc05g051060.3.1,	Solyc01g109690.1.1,	Solyc12g006800.2.1,
	Solyc06g075670.1.1,	Solyc02g080860.3.1,	Solyc11g039750.3.1,	Solyc12g098160.2.1,
	Solyc07g005140.4.1,	Solyc07g049640.3.1,	Solyc04g079600.3.1,	Solyc06g066340.4.1,
	Solyc03g083340.3.1,	Solyc08g005260.2.1,	Solyc04g150172.1.1,	Solyc06g076270.3.1,
	Solyc11g011770.3.1,	Solyc08g007170.1.1,	Solyc02g078810.4.1,	Solyc05g049960.4.1,
	Solyc06g083420.3.1,	Solyc11g017360.3.1,	Solyc07g009110.3.1,	Solyc04g011650.3.1,
	Solyc10g080460.2.1,	Solyc01g068540.3.1,	Solyc08g076400.3.1,	Solyc07g056120.3.1,
	Solyc02g080740.3.1,	Solyc01g091810.1.1,	Solyc09g060080.4.1,	Solyc12g043090.3.1,
	Solyc05g054410.4.1,	Solyc07g019550.1.1,	Solyc01g088820.2.1,	Solyc02g080730.2.1,
	Solyc06g005590.4.1			
2R-MYB	Solyc06g065100.3.1,	Solyc05g007160.3.1,	Solyc02g067760.4.1,	Solyc01g094360.3.1,

	Solyc04g078420.1.1, Solyc04g009950.3.1, Solyc12g049350.2.1, Solyc10g055410.2.1, Solyc01g009070.3.1, Solyc10g086250.2.1, Solyc05g051550.2.1, Solyc05g014290.4.1, Solyc02g079280.3.1, Solyc10g081490.2.1, Solyc03g112890.1.1, Solyc03g093930.4.1, Solyc08g065910.1.1, Solyc12g049300.2.1, Solyc08g079270.3.1, Solyc06g009410.3.1, Solyc03g059200.2.1, Solyc07g053240.3.1, Solyc12g099140.2.1, Solyc08g081500.3.1, Solyc10g086290.3.1, Solyc07g052300.3.1, Solyc09g008390.3.1, Solyc04g074170.3.1, Solyc12g008670.2.1, Solyc04g077260.3.1, Solyc07g055000.2.1, Solyc07g008010.4.1, Solyc09g090790.3.1, Solyc06g076840.3.1, Solyc04g005600.3.1, Solyc06g069850.3.1, Solyc10g083900.2.1, Solyc02g091980.2.1, Solyc03g112390.3.1, Solyc09g010840.1.1, Solyc10g019260.3.1, Solyc01g010910.2.1, Solyc05g053330.3.1, Solyc06g053610.3.1, Solyc08g008480.4.1, Solyc11g065840.3.1, Solyc04g079360.1.1, Solyc12g099120.3.1, Solyc06g071690.3.1, Solyc11g073120.2.1, Solyc07g054980.3.1, Solyc01g087130.4.1, Solyc09g090130.3.1, Solyc04g056310.3.1, Solyc05g007690.2.1, Solyc02g093740.3.1, Solyc03g019800.4.1, Solyc06g009480.3.1, Solyc08g076710.3.1, Solyc04g064540.4.1, Solyc06g083900.3.1, Solyc06g005330.3.1, Solyc05g007710.3.1, Solyc05g009230.3.1, Solyc01g005660.3.1, Solyc06g074920.3.1, Solyc03g093890.3.1, Solyc08g082890.3.1, Solyc04g014470.3.1, Solyc11g011050.2.1, Solyc12g096200.2.1, Solyc10g086270.2.1, Solyc02g088190.4.1, Solyc07g006750.3.1, Solyc10g008700.3.1, Solyc05g052850.3.1, Solyc01g079620.4.1, Solyc03g005570.3.1, Solyc05g007870.3.1, Solyc05g048830.3.1, Solyc10g005760.3.1, Solyc11g069030.3.1, Solyc04g005710.3.1, Solyc12g005640.2.1, Solyc02g082040.3.1, Solyc09g055650.3.1, Solyc08g066190.2.1, Solyc01g057910.3.1, Solyc09g011780.3.1, Solyc03g119370.2.1, Solyc02g067340.4.1, Solyc03g116100.3.1, Solyc10g005460.3.1, Solyc03g093940.2.1, Solyc01g090530.3.1, Solyc02g087960.3.1, Solyc03g025870.3.1, Solyc09g008250.4.1, Solyc12g019910.1.1, Solyc07g054960.2.1, Solyc04g050090.1.1, Solyc06g034000.2.1, Solyc01g111500.3.1, Solyc08g005870.2.1, Solyc06g074910.3.1, Solyc11g072060.3.1, Solyc04g050000.3.1, Solyc02g092930.1.1, Solyc10g005550.3.1, Solyc10g005240.3.1, Solyc06g071520.1.1, Solyc02g089190.2.1, Solyc12g099130.2.1, Solyc10g086260.3.1, Solyc03g121740.3.1, Solyc07g054840.4.1, Solyc10g044680.2.1, Solyc10g081320.1.1, Solyc07g053230.3.1, Solyc01g102340.3.1, Solyc05g008250.2.1, Solyc04g049740.1.1, Solyc06g009710.4.1, Solyc05g055030.2.1, Solyc05g053150.2.1, Solyc08g076700.1.1, Solyc06g073640.4.1	
3R-MYB	Solyc09g010820.3.1, Solyc08g068320.3.1, Solyc11g071300.2.1, Solyc08g080580.4.1	3
4R-MYB	Solyc03g119050.4.1	

Supplementary Table 7 Distribution of members of the Potato（*Solanum tuberosum*）MYB family

Subfamily	Gene ID
1R-MYB	Soltu.DM.05G009740.1, Soltu.DM.09G000550.1, Soltu.DM.11G006910.1,<br>Soltu.DM.03G012780.1, Soltu.DM.06G032070.1, Soltu.DM.10G019890.1,<br>Soltu.DM.05G025810.1, Soltu.DM.05G022630.1, Soltu.DM.05G010590.1,<br>Soltu.DM.02G033070.1, Soltu.DM.04G004780.1, Soltu.DM.12G022970.1,<br>Soltu.DM.03G023990.1, Soltu.DM.03G034270.1, Soltu.DM.11G024310.1,<br>Soltu.DM.02G033800.1, Soltu.DM.04G008810.1, Soltu.DM.03G010730.1,<br>Soltu.DM.04G018230.1, Soltu.DM.08G000740.1, Soltu.DM.02G022350.1,<br>Soltu.DM.10G000080.2, Soltu.DM.06G031130.1, Soltu.DM.08G003000.1,<br>Soltu.DM.06G004450.1, Soltu.DM.05G024900.1, Soltu.DM.09G006090.1,<br>Soltu.DM.02G004510.1, Soltu.DM.03G019030.1, Soltu.DM.12G009150.1,<br>Soltu.DM.03G027460.1, Soltu.DM.07G018870.1, Soltu.DM.04G038000.2,<br>Soltu.DM.04G019340.1, Soltu.DM.12G025790.1, Soltu.DM.10G023770.1,<br>Soltu.DM.01G035880.1, Soltu.DM.01G049290.1, Soltu.DM.07G015650.1,<br>Soltu.DM.04G000120.1, Soltu.DM.09G006100.1, Soltu.DM.11G010450.1,<br>Soltu.DM.07G010910.1, Soltu.DM.08G013550.1, Soltu.DM.06G026360.1,<br>Soltu.DM.11G010440.1, Soltu.DM.05G024580.3, Soltu.DM.05G004710.2,<br>Soltu.DM.01G034130.1, Soltu.DM.03G027560.1, Soltu.DM.03G024010.1,<br>Soltu.DM.09G006060.1, Soltu.DM.06G012330.1, Soltu.DM.09G006080.1,<br>Soltu.DM.06G017030.1, Soltu.DM.08G024350.1, Soltu.DM.05G009710.1,<br>Soltu.DM.07G018910.1, Soltu.DM.11G012750.1, Soltu.DM.12G010020.1,<br>Soltu.DM.06G013470.1, Soltu.DM.01G046340.1, Soltu.DM.05G009730.1,<br>Soltu.DM.02G007530.1, Soltu.DM.09G022120.3, Soltu.DM.07G012030.1,<br>Soltu.DM.10G021990.4, Soltu.DM.04G019420.1, Soltu.DM.03G020200.1,<br>Soltu.DM.01G020310.1, Soltu.DM.09G010050.1, Soltu.DM.11G023110.1,<br>Soltu.DM.03G002920.1, Soltu.DM.06G001980.1, Soltu.DM.09G005630.2,<br>Soltu.DM.12G023200.1, Soltu.DM.10G003970.2, Soltu.DM.10G026430.1,<br>Soltu.DM.05G024870.1, Soltu.DM.10G024990.2, Soltu.DM.03G014490.1,<br>Soltu.DM.03G036640.1, Soltu.DM.06G003930.1, Soltu.DM.02G005490.1,<br>Soltu.DM.08G023050.3, Soltu.DM.11G012550.1, Soltu.DM.11G021700.1,<br>Soltu.DM.12G002760.1, Soltu.DM.10G029880.1, Soltu.DM.06G010110.1,<br>Soltu.DM.05G009700.1, Soltu.DM.05G026640.1, Soltu.DM.02G021450.2,<br>Soltu.DM.09G007960.1, Soltu.DM.11G009850.1, Soltu.DM.06G031660.1,<br>Soltu.DM.06G019150.3, Soltu.DM.11G025400.3, Soltu.DM.07G020230.3,<br>Soltu.DM.11G016710.2, Soltu.DM.01G022460.1, Soltu.DM.09G002650.1,<br>Soltu.DM.09G008910.1, Soltu.DM.06G018400.2, Soltu.DM.09G028940.1,<br>Soltu.DM.06G032820.1, Soltu.DM.07G014720.1, Soltu.DM.01G028200.1,<br>Soltu.DM.03G036800.1, Soltu.DM.05G008880.1, Soltu.DM.05G010540.2,<br>Soltu.DM.04G004250.1, Soltu.DM.05G005130.1, Soltu.DM.12G005350.1,<br>Soltu.DM.07G016940.1, Soltu.DM.04G003420.1, Soltu.DM.04G015060.1,<br>Soltu.DM.08G021740.2, Soltu.DM.07G005530.1, Soltu.DM.10G029270.1,<br>Soltu.DM.01G025160.2, Soltu.DM.12G020640.7, Soltu.DM.03G001760.1,<br>Soltu.DM.10G026470.1, Soltu.DM.09G005500.2, Soltu.DM.10G007050.1,

	Soltu.DM.02G005520.1, Soltu.DM.02G022330.1, Soltu.DM.06G030300.1, Soltu.DM.04G030970.3, Soltu.DM.01G047820.1, Soltu.DM.02G015850.1, Soltu.DM.02G025020.1, Soltu.DM.10G019380.1, Soltu.DM.02G005500.1, Soltu.DM.05G002670.1, Soltu.DM.04G010320.1, Soltu.DM.12G027730.1, Soltu.DM.12G028120.1, Soltu.DM.11G006790.1, Soltu.DM.11G006880.1, Soltu.DM.12G006610.1, Soltu.DM.11G006750.1, Soltu.DM.02G029300.1, Soltu.DM.12G023850.1, Soltu.DM.07G017910.1, Soltu.DM.12G005330.1, Soltu.DM.01G049480.1, Soltu.DM.03G022520.1, Soltu.DM.03G029550.2, Soltu.DM.10G015800.1, Soltu.DM.03G029560.2, Soltu.DM.12G024640.1, Soltu.DM.05G020820.2, Soltu.DM.08G003720.2, Soltu.DM.04G035540.1, Soltu.DM.03G016790.1, Soltu.DM.11G011910.1, Soltu.DM.07G000240.4, Soltu.DM.04G034570.1, Soltu.DM.12G001580.1, Soltu.DM.08G022200.1, Soltu.DM.02G018190.1, Soltu.DM.06G025590.1, Soltu.DM.11G001260.2, Soltu.DM.04G035460.1, Soltu.DM.06G018850.1, Soltu.DM.10G006170.1, Soltu.DM.05G019580.1, Soltu.DM.01G049450.1, Soltu.DM.06G033730.2, Soltu.DM.02G020400.1, Soltu.DM.06G031580.1, Soltu.DM.08G001080.1, Soltu.DM.12G028910.1, Soltu.DM.07G007330.1, Soltu.DM.10G027110.3, Soltu.DM.01G052130.2, Soltu.DM.10G026950.1, Soltu.DM.01G025750.1, Soltu.DM.07G022300.1, Soltu.DM.12G010580.1, Soltu.DM.11G019810.1, Soltu.DM.04G007690.1, Soltu.DM.01G030180.1, Soltu.DM.09G017870.1, Soltu.DM.05G024910.1
2R-MYB	Soltu.DM.02G031730.1, Soltu.DM.11G020060.1, Soltu.DM.10G011320.1, Soltu.DM.05G005350.1, Soltu.DM.12G009080.1, Soltu.DM.03G014040.1, Soltu.DM.04G019440.1, Soltu.DM.07G018790.1, Soltu.DM.03G000640.1, Soltu.DM.09G012150.1, Soltu.DM.09G027500.1, Soltu.DM.04G001010.1, Soltu.DM.07G002070.1, Soltu.DM.06G000090.1, Soltu.DM.01G027520.1, Soltu.DM.06G006730.1, Soltu.DM.07G018780.1, Soltu.DM.02G028190.1, Soltu.DM.10G024480.1, Soltu.DM.01G007140.1, Soltu.DM.02G009840.1, Soltu.DM.10G020820.1, Soltu.DM.01G005960.2, Soltu.DM.08G029830.1, Soltu.DM.03G014010.1, Soltu.DM.06G012280.1, Soltu.DM.05G023150.1, Soltu.DM.10G000210.1, Soltu.DM.03G030200.1, Soltu.DM.12G025640.1, Soltu.DM.12G001830.1, Soltu.DM.04G031980.1, Soltu.DM.05G023310.1, Soltu.DM.03G017510.1, Soltu.DM.03G033800.1, Soltu.DM.07G018880.1, Soltu.DM.01G031190.1, Soltu.DM.10G020850.1, Soltu.DM.06G000300.1, Soltu.DM.02G027260.1, Soltu.DM.08G022520.1, Soltu.DM.01G041210.1, Soltu.DM.12G001820.1, Soltu.DM.05G004700.1, Soltu.DM.10G000640.1, Soltu.DM.04G000830.1, Soltu.DM.09G001100.1, Soltu.DM.01G009960.1, Soltu.DM.09G002130.1, Soltu.DM.07G018840.1, Soltu.DM.04G033180.1, Soltu.DM.06G000270.1, Soltu.DM.03G036540.1, Soltu.DM.04G004510.3, Soltu.DM.04G006820.1, Soltu.DM.04G025530.1, Soltu.DM.06G026660.1, Soltu.DM.06G012270.1, Soltu.DM.07G018810.1, Soltu.DM.03G019140.1, Soltu.DM.07G018770.1, Soltu.DM.07G018860.1, Soltu.DM.08G028250.1, Soltu.DM.06G012300.1, Soltu.DM.02G010440.1, Soltu.DM.06G030290.1, Soltu.DM.02G021850.1, Soltu.DM.06G004440.3, Soltu.DM.05G019450.1, Soltu.DM.08G002970.1, Soltu.DM.08G003080.1, Soltu.DM.04G034270.1,

	Soltu.DM.04G021200.1, Soltu.DM.12G023290.1, Soltu.DM.06G012310.1, Soltu.DM.07G028550.1, Soltu.DM.03G014020.1, Soltu.DM.10G020770.1, Soltu.DM.03G026770.1, Soltu.DM.06G034280.1, Soltu.DM.01G018600.1, Soltu.DM.09G026730.1, Soltu.DM.11G026620.1, Soltu.DM.01G033280.1, Soltu.DM.07G018800.1, Soltu.DM.11G025050.1, Soltu.DM.07G018850.1, Soltu.DM.06G029020.1, Soltu.DM.02G031540.1, Soltu.DM.05G021400.1, Soltu.DM.09G005190.1, Soltu.DM.09G006830.1, Soltu.DM.08G004870.1, Soltu.DM.07G017700.1, Soltu.DM.10G017480.1, Soltu.DM.05G005150.1, Soltu.DM.10G020840.1, Soltu.DM.05G022870.1, Soltu.DM.10G026020.1, Soltu.DM.07G018830.1, Soltu.DM.02G030600.1, Soltu.DM.08G025560.1, Soltu.DM.10G000540.1, Soltu.DM.07G003440.1, Soltu.DM.01G024670.1, Soltu.DM.10G000860.1, Soltu.DM.10G020780.1, Soltu.DM.09G005350.1, Soltu.DM.07G018820.1, Soltu.DM.06G012250.1, Soltu.DM.02G018840.1, Soltu.DM.07G019030.1, Soltu.DM.06G025970.1, Soltu.DM.10G009220.1, Soltu.DM.03G008820.1, Soltu.DM.04G019380.1, Soltu.DM.05G005360.1, Soltu.DM.04G029740.1, Soltu.DM.10G025860.1, Soltu.DM.11G022400.1, Soltu.DM.11G002540.1, Soltu.DM.10G004620.1, Soltu.DM.07G018900.1, Soltu.DM.01G001190.1, Soltu.DM.03G014030.1, Soltu.DM.06G020450.1, Soltu.DM.05G025580.1, Soltu.DM.03G026020.1, Soltu.DM.12G001810.1, Soltu.DM.08G022510.1, Soltu.DM.05G003270.1, Soltu.DM.05G006220.1, Soltu.DM.06G014570.1, Soltu.DM.01G051040.1, Soltu.DM.06G032140.1, Soltu.DM.08G002990.1, Soltu.DM.03G014050.1, Soltu.DM.06G026840.1
3R-MYB	Soltu.DM.11G023940.1, Soltu.DM.08G027230.1, Soltu.DM.09G002150.1, Soltu.DM.08G017470.1
4R-MYB	Soltu.DM.03G033440.2

Supplementary Table 8 Distribution of members of the Tobacco（*Nicotiana tabacum*）MYB family

Subfamily		Gene ID	
1R-MYB	Nitab0000185g0060.1,	Nitab0010633g0050.1,	Nitab0000556g0050.1,
	Nitab0007025g0030.1,	Nitab0001505g0030.1,	Nitab0001786g0040.1,
	Nitab0005855g0010.1,	Nitab0000153g0080.1,	Nitab0000008g0850.1,
	Nitab0001932g0010.1,	Nitab0004030g0060.1,	Nitab0000945g0020.1,
	Nitab0001889g0060.1,	Nitab0008712g0020.1,	Nitab0000010g0120.1,
	Nitab0001047g0070.1,	Nitab0000556g0040.1,	Nitab0000947g0040.1,
	Nitab0000745g0220.1,	Nitab0000278g0160.1,	Nitab0005115g0010.1,
	Nitab0003465g0020.1,	Nitab0001271g0020.1,	Nitab0008659g0010.1,
	Nitab0004621g0030.1,	Nitab0005396g0070.1,	Nitab0002100g0160.1,
	Nitab0001369g0030.1,	Nitab0001769g0160.1,	Nitab0001465g0040.1,
	Nitab0000745g0040.1,	Nitab0009579g0010.1,	Nitab0001441g0080.1,
	Nitab0000956g0010.1,	Nitab0008556g0010.1,	Nitab0000410g0050.1,
	Nitab0000003g0750.1,	Nitab0005352g0010.1,	Nitab0000363g0160.1,
	Nitab0000107g0030.1,	Nitab0001372g0100.1,	Nitab0005214g0060.1,
	Nitab0000766g0070.1,	Nitab0011191g0010.1,	Nitab0005116g0040.1,
	Nitab0004632g0010.1,	Nitab0002184g0040.1,	Nitab0001183g0050.1,
	Nitab0000712g0010.1,	Nitab0010431g0030.1,	Nitab0004776g0070.1,
	Nitab0000271g0130.1,	Nitab0000119g0220.1,	Nitab0000611g0270.1,
	Nitab0008644g0010.1,	Nitab0004081g0040.1,	Nitab0000233g0120.1,
	Nitab0000691g0100.1,	Nitab0008949g0040.1,	Nitab0010936g0020.1,
	Nitab0000895g0070.1,	Nitab0000185g0040.1,	Nitab0000613g0190.1,
	Nitab0005769g0080.1,	Nitab0000642g0100.1,	Nitab0009714g0010.1,
	Nitab0000040g0010.1,	Nitab0003973g0010.1,	Nitab0004821g0020.1,
	Nitab0002087g0010.1,	Nitab0003096g0050.1,	Nitab0000369g0020.1,
	Nitab0000855g0020.1,	Nitab0001180g0220.1,	Nitab0004614g0010.1,
	Nitab0000720g0110.1,	Nitab0001707g0110.1,	Nitab0003927g0090.1,
	Nitab0007610g0020.1,	Nitab0004168g0010.1,	Nitab0000014g0120.1,
	Nitab0000005g0090.1,	Nitab0000002g0550.1,	Nitab0002332g0030.1,
	Nitab0001756g0010.1,	Nitab0000202g0460.1,	Nitab0003820g0040.1,
	Nitab0004551g0040.1,	Nitab0006241g0070.1,	Nitab0000712g0040.1,
	Nitab0000559g0130.1,	Nitab0001701g0100.1,	Nitab0000667g0180.1,
	Nitab0009466g0040.1,	Nitab0001794g0100.1,	Nitab0005451g0010.1,
	Nitab0005267g0010.1,	Nitab0002055g0110.1,	Nitab0001584g0120.1,
	Nitab0004133g0010.1,	Nitab0006560g0010.1,	Nitab0001908g0030.1,
	Nitab0002875g0030.1,	Nitab0000642g0170.1,	Nitab0000003g0210.1,
	Nitab0000540g0020.1,	Nitab0021887g0010.1,	Nitab0000713g0070.1,
	Nitab0000574g0110.1,	Nitab0003321g0030.1,	Nitab0006693g0010.1,
	Nitab0000262g0020.1,	Nitab0008857g0030.1,	Nitab0001172g0110.1,
	Nitab0011903g0010.1,	Nitab0006640g0010.1,	Nitab0000322g0040.1,
	Nitab0010836g0030.1,	Nitab0001590g0030.1,	Nitab0007450g0010.1,
	Nitab0002896g0030.1,	Nitab0004456g0060.1,	Nitab0000943g0010.1,
	Nitab0009546g0010.1,	Nitab0004087g0040.1,	Nitab0000142g0120.1,

	Nitab0000248g0120.1,	Nitab0004854g0060.1,	Nitab0002530g0070.1,
	Nitab0011076g0020.1,	Nitab0000036g0090.1,	Nitab0002040g0020.1,
	Nitab0002631g0020.1,	Nitab0000386g0160.1,	Nitab0003606g0040.1,
	Nitab0001519g0120.1,	Nitab0000146g0050.1,	Nitab0001702g0050.1,
	Nitab0014621g0010.1,	Nitab0010401g0020.1,	Nitab0000061g0100.1,
	Nitab0002389g0010.1,	Nitab0006980g0060.1,	Nitab0000324g0240.1,
	Nitab0004903g0060.1,	Nitab0006794g0060.1,	Nitab0002963g0080.1,
	Nitab0000463g0190.1,	Nitab0006916g0030.1,	Nitab0006629g0020.1,
	Nitab0000101g0340.1,	Nitab0000476g0270.1,	Nitab0000740g0040.1,
	Nitab0001097g0090.1,	Nitab0003610g0020.1,	Nitab0012878g0020.1,
	Nitab0005180g0030.1,	Nitab0000021g1050.1,	Nitab0000058g0230.1,
	Nitab0000969g0020.1,	Nitab0009641g0020.1,	Nitab0001534g0020.1,
	Nitab0002948g0070.1,	Nitab0000102g0300.1,	Nitab0004892g0040.1,
	Nitab0005003g0020.1,	Nitab0002293g0030.1,	Nitab0008054g0010.1,
	Nitab0006133g0010.1,	Nitab0002055g0150.1,	Nitab0008332g0020.1,
	Nitab0000123g0580.1,	Nitab0002606g0020.1,	Nitab0000850g0070.1,
	Nitab0004479g0040.1,	Nitab0006049g0020.1,	Nitab0000178g0130.1,
	Nitab0001176g0100.1,	Nitab0000676g0220.1,	Nitab0003295g0220.1,
	Nitab0012253g0010.1,	Nitab0002072g0040.1,	Nitab0000088g0060.1,
	Nitab0001975g0010.1,	Nitab0000543g0010.1,	Nitab0004025g0060.1,
	Nitab0000002g0180.1,	Nitab0001960g0060.1,	Nitab0000838g0010.1,
	Nitab0011592g0010.1,	Nitab0002620g0010.1,	Nitab0000621g0130.1,
	Nitab0010689g0010.1,	Nitab0006963g0020.1,	Nitab0001318g0010.1,
	Nitab0001599g0170.1,	Nitab0010683g0010.1,	Nitab0000629g0030.1,
	Nitab0001416g0010.1,	Nitab0000208g0170.1,	Nitab0004327g0030.1,
	Nitab0000616g0080.1,	Nitab0004802g0050.1,	Nitab0002024g0040.1,
	Nitab0004143g0010.1,	Nitab0000016g0360.1,	Nitab0004658g0020.1,
	Nitab0003100g0090.1,	Nitab0001091g0060.1,	Nitab0002462g0050.1,
	Nitab0001607g0150.1,	Nitab0002076g0010.1,	Nitab0001933g0010.1,
	Nitab0003861g0040.1,	Nitab0013588g0020.1,	Nitab0002632g0070.1,
	Nitab0003484g0070.1,	Nitab0003726g0010.1,	Nitab0000672g0080.1,
	Nitab0000916g0020.1,	Nitab0005194g0010.1,	Nitab0000017g0200.1,
	Nitab0000444g0200.1,	Nitab0002803g0020.1,	Nitab0004241g0060.1,
	Nitab0000109g0160.1,	Nitab0005913g0020.1,	Nitab0002241g0010.1,
	Nitab0001777g0040.1,	Nitab0011083g0010.1,	Nitab0008908g0020.1,
	Nitab0000440g0010.1,	Nitab0000131g0210.1,	Nitab0000354g0150.1,
	Nitab0006324g0050.1,	Nitab0000041g0320.1,	Nitab0003147g0060.1,
	Nitab0004511g0010.1,	Nitab0000784g0110.1,	Nitab0007848g0010.1,
	Nitab0000388g0040.1,	Nitab0003889g0040.1,	Nitab0004991g0030.1,
	Nitab0004835g0040.1,	Nitab0007123g0020.1,	Nitab0001663g0280.1,
	Nitab0000336g0010.1,	Nitab0000605g0080.1,	Nitab0004757g0010.1,
	Nitab0028143g0010.1,	Nitab0004531g0020.1,	Nitab0000147g0130.1,
	Nitab0004560g0010.1,	Nitab0002478g0030.1,	Nitab0000146g0010.1,
	Nitab0000062g0150.1,	Nitab0005233g0020.1,	Nitab0001432g0050.1,
	Nitab0000011g0070.1,	Nitab0000570g0280.1,	Nitab0001759g0030.1,

	Nitab0003711g0030.1, Nitab0001091g0100.1, Nitab0002224g0140.1, Nitab0005559g0030.1, Nitab0001522g0090.1, Nitab0002811g0050.1, Nitab0000145g0010.1, Nitab0010430g0010.1, Nitab0003836g0060.1, Nitab0012578g0010.1, Nitab0004550g0020.1, Nitab0008336g0010.1, Nitab0001164g0090.1, Nitab0007572g0030.1, Nitab0005894g0050.1, Nitab0001083g0040.1, Nitab0011729g0010.1, Nitab0000386g0010.1, Nitab0000308g0160.1, Nitab0002881g0090.1, Nitab0000222g0120.1, Nitab0000326g0260.1, Nitab0001199g0120.1, Nitab0001702g0030.1, Nitab0004186g0020.1, Nitab0006815g0010.1, Nitab0000109g0060.1, Nitab0006900g0010.1, Nitab0001632g0010.1, Nitab0010431g0010.1, Nitab0001409g0040.1, Nitab0005366g0010.1, Nitab0000308g0210.1, Nitab0010752g0020.1, Nitab0011729g0030.1, Nitab0000185g0200.1, Nitab0006594g0090.1, Nitab0001088g0210.1, Nitab0007468g0010.1, Nitab0002465g0020.1, Nitab0009217g0030.1, Nitab0006031g0030.1, Nitab0000045g0240.1, Nitab0002223g0030.1, Nitab0001564g0060.1, Nitab0000463g0100.1, Nitab0003758g0060.1, Nitab0003856g0030.1, Nitab0012999g0010.1, Nitab0005160g0040.1, Nitab0001094g0080.1, Nitab0001326g0080.1, Nitab0000042g0050.1, Nitab0003114g0010.1, Nitab0009749g0020.1, Nitab0003779g0070.1, Nitab0001820g0010.1, Nitab0004694g0040.1, Nitab0002872g0040.1, Nitab0001002g0020.1, Nitab0008982g0040.1, Nitab0002117g0090.1, Nitab0000272g0060.1, Nitab0008900g0010.1, Nitab0001097g0040.1
2R-MYB	Nitab0003478g0050.1, Nitab0000578g0120.1, Nitab0000889g0030.1, Nitab0000057g0160.1, Nitab0003062g0070.1, Nitab0001087g0070.1, Nitab0010133g0010.1, Nitab0000081g0200.1, Nitab0001863g0170.1, Nitab0002738g0010.1, Nitab0004051g0020.1, Nitab0000003g0660.1, Nitab0004704g0020.1, Nitab0000769g0140.1, Nitab0012173g0010.1, Nitab0002560g0010.1, Nitab0000109g0340.1, Nitab0002728g0020.1, Nitab0004482g0030.1, Nitab0000232g0160.1, Nitab0001334g0020.1, Nitab0000044g0310.1, Nitab0000278g0070.1, Nitab0000225g0010.1, Nitab0002030g0030.1, Nitab0002529g0070.1, Nitab0003242g0050.1, Nitab0006658g0010.1, Nitab0000568g0090.1, Nitab0000747g0030.1, Nitab0002163g0070.1, Nitab0006880g0040.1, Nitab0000141g0070.1, Nitab0002906g0080.1, Nitab0002293g0010.1, Nitab0004551g0030.1, Nitab0000906g0090.1, Nitab0000121g0260.1, Nitab0006037g0010.1, Nitab0001050g0010.1, Nitab0001442g0010.1, Nitab0001814g0020.1, Nitab0004662g0010.1, Nitab0002560g0020.1, Nitab0007938g0010.1, Nitab0002152g0050.1, Nitab0000302g0200.1, Nitab0008050g0020.1, Nitab0000210g0060.1, Nitab0002217g0020.1, Nitab0003867g0010.1, Nitab0000244g0260.1, Nitab0004379g0020.1, Nitab0004530g0020.1, Nitab0018794g0010.1, Nitab0005512g0020.1, Nitab0003163g0090.1, Nitab0006144g0030.1, Nitab0002714g0010.1, Nitab0000387g0040.1, Nitab0000040g0170.1, Nitab0000842g0120.1, Nitab0002222g0050.1, Nitab0000080g0070.1, Nitab0000436g0250.1, Nitab0000103g0280.1, Nitab0008488g0010.1, Nitab0003781g0060.1, Nitab0006318g0060.1,

	Nitab0001213g0100.1, Nitab0005586g0010.1, Nitab0000465g0070.1, Nitab0001622g0080.1, Nitab0000994g0010.1, Nitab0001616g0010.1, Nitab0002578g0010.1, Nitab0000325g0020.1, Nitab0001019g0020.1, Nitab0000564g0080.1, Nitab0002250g0030.1, Nitab0003324g0040.1, Nitab0000201g0150.1, Nitab0005973g0020.1, Nitab0006450g0010.1, Nitab0003212g0030.1, Nitab0000634g0210.1, Nitab0006265g0060.1, Nitab0001081g0060.1, Nitab0000063g0230.1, Nitab0007679g0010.1, Nitab0005060g0020.1, Nitab0007287g0010.1, Nitab0004437g0020.1, Nitab0000683g0060.1, Nitab0001007g0040.1, Nitab0003500g0020.1, Nitab0005004g0050.1, Nitab0000247g0080.1, Nitab0002721g0050.1, Nitab0010251g0010.1, Nitab0002714g0060.1, Nitab0008039g0010.1, Nitab0004623g0050.1, Nitab0000365g0050.1, Nitab0000103g0300.1, Nitab0001318g0060.1, Nitab0004170g0030.1, Nitab0000769g0210.1, Nitab0000797g0020.1, Nitab0000895g0130.1, Nitab0000569g0180.1, Nitab0000021g0690.1, Nitab0017504g0010.1, Nitab0000189g0200.1, Nitab0001373g0030.1, Nitab0016704g0010.1, Nitab0002352g0080.1, Nitab0003845g0050.1, Nitab0001730g0050.1, Nitab0003683g0020.1, Nitab0012287g0020.1, Nitab0008924g0010.1, Nitab0005304g0030.1, Nitab0000363g0320.1, Nitab0001984g0040.1, Nitab0000083g0200.1, Nitab0005842g0010.1, Nitab0000008g0380.1, Nitab0000041g0400.1, Nitab0000610g0290.1, Nitab0000514g0130.1, Nitab0000428g0150.1, Nitab0000958g0020.1, Nitab0000649g0090.1, Nitab0006804g0010.1, Nitab0003179g0030.1, Nitab0000541g0100.1, Nitab0000103g0310.1, Nitab0002160g0080.1, Nitab0009971g0010.1, Nitab0005224g0050.1, Nitab0002403g0060.1, Nitab0001041g0130.1, Nitab0000010g0220.1, Nitab0009259g0010.1, Nitab0001966g0010.1, Nitab0000918g0020.1, Nitab0007139g0020.1, Nitab0000205g0020.1, Nitab0004993g0020.1, Nitab0007042g0010.1, Nitab0001097g0050.1, Nitab0000716g0090.1, Nitab0004193g0070.1, Nitab0001163g0150.1, Nitab0000673g0080.1, Nitab0007777g0020.1, Nitab0006516g0020.1, Nitab0004118g0080.1, Nitab0003631g0010.1, Nitab0011004g0010.1, Nitab0000068g0400.1, Nitab0001074g0040.1, Nitab0000614g0110.1, Nitab0000174g0270.1, Nitab0005769g0040.1, Nitab0005708g0020.1, Nitab0001176g0010.1, Nitab0004195g0050.1, Nitab0001180g0160.1,Nitab0000274g0200.1, Nitab0000844g0110.1, Nitab0005819g0010.1, Nitab0001048g0070.1, Nitab0000116g0020.1, Nitab0000303g0330.1, Nitab0001031g0090.1, Nitab0001612g0030.1,Nitab0008604g0010.1, Nitab0002137g0010.1, Nitab0003511g0010.1, Nitab0005019g0040.1,Nitab0008289g0010.1,Nitab0001295g0260.1,Nitab0000063g0150.1, Nitab0000070g0110.1, Nitab0006893g0010.1, Nitab0000958g0010.1, Nitab0000271g0230.1, Nitab0002300g0110.1, Nitab0001818g0030.1, Nitab0004786g0040.1, Nitab0002626g0050.1, Nitab0011760g0030.1, Nitab0003061g0020.1, Nitab0001186g0080.1, Nitab0007325g0020.1
3R-MYB	Nitab0000103g0290.1, Nitab0007126g0010.1, Nitab0006677g0010.1, Nitab0000052g0200.1,Nitab0000349g0300.1, Nitab0010472g0020.1, Nitab0001567g0080.1, Nitab0003845g0010.1, Nitab0001911g0080.1, Nitab0000664g0320.1, Nitab0005648g0010.1,
4R-MYB	Nitab0012734g0010.1, Nitab0001158g0050.1

Supplementary Table 9 Distribution of members of the Soybean（*Glycine max*）MYB family

Subfamily	Gene ID
1R-MYB	Glyma.14G191700.1, Glyma.18G134200.11, Glyma.08G290100.11, Glyma.02G224900.1,<br>Glyma.20G011700.2, Glyma.11G176500.1, Glyma.18G065200.1, Glyma.17G121000.1,<br>Glyma.18G197500.1, Glyma.07G146300.1, Glyma.05G013000.1, Glyma.03G078000.1,<br>Glyma.01G112700.2, Glyma.11G227200.2, Glyma.18G030300.2, Glyma.10G277800.1,<br>Glyma.20G111800.1, Glyma.20G178500.2, Glyma.07G175500.2, Glyma.02G254200.2,<br>Glyma.15G034500.2, Glyma.14G062200.2, Glyma.10G212600.3, Glyma.13G071701.1,<br>Glyma.19G085700.1, Glyma.19G083700.1, Glyma.04G184200.1, Glyma.03G258700.2,<br>Glyma.11G211643.1, Glyma.18G044200.1, Glyma.14G210600.1, Glyma.16G017400.7,<br>Glyma.02G241000.1, Glyma.09G167900.1, Glyma.08G188900.1, Glyma.04G014400.2,<br>Glyma.12G168800.1, Glyma.07G048500.1, Glyma.06G188400.1, Glyma.12G044200.8,<br>Glyma.19G260900.8, Glyma.03G261800.4, Glyma.20G062300.2, Glyma.11G118900.4,<br>Glyma.13G031300.3, Glyma.14G153000.2, Glyma.17G229800.1, Glyma.13G006200.17,<br>Glyma.13G038100.1, Glyma.16G217700.1, Glyma.10G048500.3, Glyma.13G136300.5,<br>Glyma.05G032200.1, Glyma.17G094400.1, Glyma.15G117100.2, Glyma.09G012500.1,<br>Glyma.04G247200.3, Glyma.06G115600.3, Glyma.10G118400.2, Glyma.20G060250.1,<br>Glyma.14G093800.1, Glyma.19G178000.2, Glyma.04G177300.1, Glyma.06G187600.1,<br>Glyma.14G120600.3, Glyma.16G032600.1, Glyma.07G066100.1, Glyma.06G051900.1,<br>Glyma.08G029400.1, Glyma.05G222600.1, Glyma.04G051000.2, Glyma.01G003000.1,<br>Glyma.18G237700.3, Glyma.01G038600.1, Glyma.02G026300.1, Glyma.04G004700.1,<br>Glyma.15G019400.1, Glyma.13G354800.1, Glyma.06G014400.1, Glyma.05G062300.1,<br>Glyma.07G007200.2, Glyma.08G189900.1, Glyma.10G230700.5, Glyma.13G005900.1,<br>Glyma.13G006100.1, Glyma.11G142900.3, Glyma.17G144100.1, Glyma.08G293300.1,<br>Glyma.06G120200.3, Glyma.18G129900.1, Glyma.12G172250.1, Glyma.20G162800.3,<br>Glyma.20G060050.1, Glyma.13G038200.1, Glyma.16G178700.1, Glyma.09G131400.5,<br>Glyma.04G243100.1, Glyma.05G189200.1, Glyma.15G135100.3, Glyma.09G196500.1,<br>Glyma.13G083601.1, Glyma.12G195200.1, Glyma.07G074500.1, Glyma.09G029300.2,<br>Glyma.19G247600.3, Glyma.13G005833.1, Glyma.01G228200.1, Glyma.11G010900.7,<br>Glyma.16G152200.1, Glyma.02G070900.2, Glyma.13G307300.2, Glyma.03G166400.1,<br>Glyma.19G167500.1, Glyma.13G126200.9, Glyma.10G039700.13, Glyma.10G153800.30,<br>Glyma.14G202600.1, Glyma.17G152800.3, Glyma.20G234500.16, Glyma.12G060200.1,<br>Glyma.05G239500.2, Glyma.11G136600.3, Glyma.02G178100.2, Glyma.18G075100.1,<br>Glyma.09G017400.12, Glyma.02G162600.1, Glyma.15G123100.2, Glyma.12G042900.1,<br>Glyma.01G200800.8, Glyma.11G183400.1, Glyma.08G149600.1, Glyma.03G250000.4,<br>Glyma.05G070200.2, Glyma.19G127000.1, Glyma.06G143600.2, Glyma.12G100600.1,<br>Glyma.03G123400.2, Glyma.06G303100.3, Glyma.11G041300.1, Glyma.06G097100.2,<br>Glyma.09G211400.1, Glyma.04G148800.2, Glyma.02G214600.1, Glyma.13G316600.2,<br>Glyma.04G095300.5, Glyma.12G184700.1, Glyma.15G269600.2, Glyma.19G146600.1,<br>Glyma.11G117200.1, Glyma.07G074300.1, Glyma.01G194600.1, Glyma.11G186600.1,<br>Glyma.11G047300.1, Glyma.03G143600.2, Glyma.12G089100.1, Glyma.12G117700.1,<br>Glyma.17G160500.2, Glyma.06G289300.1, Glyma.06G216400.2, Glyma.13G294300.1,<br>Glyma.12G206600.1, Glyma.13G303100.1, Glyma.07G037100.5, Glyma.07G153900.2,<br>Glyma.18G052700.2, Glyma.05G106000.2, Glyma.02G177800.4, Glyma.15G109100.1,

	Glyma.10G057800.6, Glyma.18G204900.2, Glyma.13G228900.6, Glyma.13G144600.2, Glyma.15G145200.3, Glyma.07G243300.1, Glyma.09G004500.1, Glyma.17G030600.1, Glyma.01G009600.1, Glyma.04G031300.3, Glyma.09G113000.2, Glyma.09G040000.3, Glyma.15G083600.1, Glyma.07G209500.3, Glyma.02G135400.1, Glyma.12G213900.1, Glyma.05G158900.1, Glyma.08G116700.1, Glyma.18G010800.1, Glyma.18G113400.1, Glyma.02G157300.1, Glyma.06G031400.1, Glyma.11G246400.5, Glyma.15G206200.1, Glyma.16G128300.2, Glyma.20G185167.1, Glyma.05G144500.1, Glyma.07G171200.2, Glyma.09G098600.1, Glyma.02G048100.1, Glyma.15G215000.1, Glyma.16G006500.4, Glyma.04G221800.1, Glyma.02G085900.2, Glyma.08G100900.1, Glyma.14G031500.1, Glyma.07G229800.1, Glyma.17G024600.3, Glyma.13G290100.1, Glyma.12G211600.1, Glyma.19G047702.2, Glyma.02G282900.1, Glyma.13G197800.2, Glyma.19G002100.1, Glyma.14G110600.2, Glyma.20G035300.1, Glyma.01G123600.1, Glyma.09G113100.1, Glyma.03G051400.1, Glyma.03G237000.1, Glyma.15G236400.1, Glyma.07G178500.1, Glyma.11G058600.1, Glyma.17G217100.1, Glyma.01G224900.2, Glyma.06G063500.1, Glyma.20G193600.4, Glyma.01G183700.1, Glyma.10G196600.2, Glyma.04G012600.2, Glyma.15G263700.9, Glyma.03G003500.3, Glyma.08G163500.2, Glyma.18G201800.1, Glyma.11G018200.1, Glyma.19G122700.3, Glyma.01G049100.2, Glyma.16G093800.3, Glyma.20G186500.1, Glyma.17G178500.3, Glyma.13G155400.1, Glyma.05G239800.2, Glyma.13G184500.1, Glyma.04G035700.4, Glyma.07G158600.1, Glyma.09G017300.1, Glyma.04G062500.1, Glyma.15G123000.1, Glyma.03G125000.1, Glyma.04G132200.1, Glyma.04G151000.2, Glyma.17G119600.1, Glyma.04G031700.1, Glyma.06G031800.1, Glyma.07G106800.1, Glyma.05G011500.1, Glyma.10G204200.1, Glyma.16G198800.1, Glyma.06G035700.1, Glyma.20G009800.6, Glyma.06G213400.1, Glyma.17G076000.3, Glyma.09G171100.1, Glyma.06G309200.1, Glyma.09G112200.10, Glyma.17G246200.1, Glyma.14G079000.2, Glyma.10G281000.4, Glyma.20G108600.1, Glyma.02G108500.2, Glyma.03G221500.3, Glyma.20G006000.1, Glyma.01G086700.2, Glyma.02G098800.1, Glyma.18G234900.2, Glyma.11G138700.16, Glyma.19G218500.2, Glyma.08G046800.1, Glyma.18G073300.1, Glyma.19G127950.1, Glyma.03G081300.1, Glyma.10G269601.2, Glyma.01G014800.1, Glyma.14G067100.1, Glyma.07G133000.5, Glyma.07G192000.1, Glyma.09G257700.3, Glyma.09G207300.1, Glyma.18G182400.1, Glyma.19G051651.1, Glyma.15G037300.2, Glyma.02G242100.1, Glyma.02G249400.4, Glyma.14G211900.3, Glyma.12G062300.1, Glyma.02G055900.1, Glyma.18G043000.12, Glyma.19G220800.1, Glyma.20G026500.1, Glyma.11G213700.2, Glyma.08G352600.3, Glyma.13G337100.1, Glyma.14G074500.2, Glyma.02G055800.1, Glyma.07G249800.2, Glyma.16G138400.1, Glyma.03G223600.1, Glyma.01G173900.1
2R-MYB	Glyma.07G235900.1, Glyma.16G072700.2, Glyma.13G303200.1, Glyma.20G117000.1, Glyma.01G004900.2, Glyma.02G110200.1, Glyma.17G162100.1, Glyma.14G119000.1, Glyma.13G038500.1, Glyma.07G141100.1, Glyma.03G221700.1, Glyma.19G006500.1, Glyma.02G225700.1, Glyma.19G164600.1, Glyma.03G007500.1, Glyma.05G109200.1, Glyma.02G110000.1, Glyma.07G038000.1, Glyma.10G273000.1, Glyma.17G231900.1, Glyma.10G010400.1, Glyma.18G071600.1, Glyma.14G069100.3, Glyma.19G025000.1, Glyma.05G211200.1, Glyma.12G241400.2, Glyma.02G019300.2, Glyma.14G086500.1, Glyma.03G013300.1, Glyma.02G005600.1, Glyma.03G073756.1, Glyma.09G001500.1, Glyma.05G005400.1, Glyma.20G184200.1, Glyma.10G014600.1, Glyma.04G042300.1, Glyma.02G110100.1, Glyma.01G190100.1, Glyma.08G168100.1, Glyma.04G205100.1,

Glyma.03G073764.1, Glyma.01G044300.7, Glyma.10G132200.3, Glyma.18G273300.1,
Glyma.17G088600.1, Glyma.01G211500.1, Glyma.06G103300.1, Glyma.04G101900.1,
Glyma.05G051700.1, Glyma.15G144000.1, Glyma.19G038100.1, Glyma.20G013000.1,
Glyma.10G139000.1, Glyma.02G108800.1, Glyma.15G264000.1, Glyma.14G090900.1,
Glyma.11G194100.1, Glyma.18G261700.2, Glyma.11G215800.1, Glyma.05G027000.1,
Glyma.15G025500.1, Glyma.10G059000.3, Glyma.15G176000.2, Glyma.06G312900.2,
Glyma.20G184100.1, Glyma.05G234600.1, Glyma.05G040700.1, Glyma.07G228600.1,
Glyma.05G061900.1, Glyma.04G242200.1, Glyma.11G107100.1, Glyma.12G199100.1,
Glyma.19G062000.1, Glyma.18G151400.2, Glyma.06G003800.1, Glyma.19G224600.1,
Glyma.15G041100.1, Glyma.11G034900.1, Glyma.15G225300.5, Glyma.02G124300.1,
Glyma.03G081900.1, Glyma.06G300100.1, Glyma.06G299900.1, Glyma.20G082300.1,
Glyma.01G016600.1, Glyma.07G054000.1, Glyma.19G061600.1, Glyma.08G005400.1,
Glyma.17G158000.1, Glyma.13G032200.1, Glyma.06G036800.1, Glyma.10G236400.2,
Glyma.07G008500.1, Glyma.06G050000.1, Glyma.12G104600.1, Glyma.20G223300.3,
Glyma.17G037500.1, Glyma.20G199300.1, Glyma.07G189300.1, Glyma.19G248100.1,
Glyma.12G057900.1, Glyma.03G073774.1, Glyma.10G006600.1, Glyma.18G191200.1,
Glyma.13G066600.1, Glyma.06G178600.1, Glyma.04G036700.1, Glyma.19G061300.1,
Glyma.08G191500.1, Glyma.17G031600.1, Glyma.05G224300.1, Glyma.01G107500.1,
Glyma.18G095600.4, Glyma.01G207600.1, Glyma.12G199600.1, Glyma.10G054700.1,
Glyma.10G037001.1, Glyma.06G299300.1, Glyma.12G199200.1, Glyma.08G336500.1,
Glyma.20G047600.1, Glyma.17G085651.1, Glyma.04G004100.1, Glyma.17G232220.1,
Glyma.08G059900.1, Glyma.06G193600.1, Glyma.06G300200.1, Glyma.20G034100.1,
Glyma.13G339900.1, Glyma.02G253100.1, Glyma.09G032100.1, Glyma.20G209700.1,
Glyma.18G181300.1, Glyma.14G058100.2, Glyma.19G119300.1, Glyma.01G222200.1,
Glyma.10G165800.1, Glyma.11G009400.1, Glyma.09G238800.1, Glyma.12G177700.1,
Glyma.06G300000.1, Glyma.10G191000.1, Glyma.03G225200.1, Glyma.09G139000.1,
Glyma.14G214500.1, Glyma.09G206200.1, Glyma.18G230600.1, Glyma.19G218800.1,
Glyma.07G110700.1, Glyma.16G092100.2, Glyma.06G300300.1, Glyma.14G154400.1,
Glyma.05G098200.1, Glyma.02G013900.1, Glyma.02G264900.5, Glyma.10G010300.1,
Glyma.07G216000.1, Glyma.19G257400.1, Glyma.19G022100.1, Glyma.20G090700.1,
Glyma.09G235300.1, Glyma.12G079800.1, Glyma.01G196200.1, Glyma.19G184500.1,
Glyma.13G247200.1, Glyma.06G195600.1, Glyma.14G039200.3, Glyma.10G206500.1,
Glyma.18G261400.3, Glyma.13G128500.1, Glyma.09G038900.1, Glyma.08G017600.1,
Glyma.09G235600.1, Glyma.07G037700.1, Glyma.07G242600.1, Glyma.16G073000.1,
Glyma.16G007100.1, Glyma.07G073000.2, Glyma.14G091500.1, Glyma.03G250600.1,
Glyma.09G234900.1, Glyma.13G145800.1, Glyma.13G282100.1, Glyma.12G237500.1,
Glyma.19G214900.1, Glyma.05G038500.1, Glyma.09G261600.1, Glyma.17G245200.1,
Glyma.20G157900.2, Glyma.08G317626.1, Glyma.08G298200.1, Glyma.18G259100.1,
Glyma.06G160500.1, Glyma.06G181800.2, Glyma.17G099800.1, Glyma.13G109100.1,
Glyma.10G206400.1, Glyma.10G165900.8, Glyma.13G073400.2, Glyma.03G227700.2,
Glyma.03G006600.1, Glyma.04G176700.1, Glyma.08G163200.1, Glyma.08G042100.1,
Glyma.16G007200.1, Glyma.09G235100.1, Glyma.18G273400.1, Glyma.19G264200.1,
Glyma.11G133700.1, Glyma.17G167100.2, Glyma.02G006800.1, Glyma.11G108400.1,
Glyma.13G203100.1, Glyma.13G063200.1, Glyma.17G133800.6, Glyma.05G006100.1,
Glyma.17G237900.1, Glyma.18G066400.1, Glyma.13G309200.1, Glyma.03G115400.1,

	Glyma.15G259400.3, Glyma.11G021600.1, Glyma.02G244600.1, Glyma.02G247100.1, Glyma.07G132400.2, Glyma.01G049600.1, Glyma.13G141900.1, Glyma.01G051700.1, Glyma.09G169300.1, Glyma.04G187300.1, Glyma.13G187500.6, Glyma.13G050400.1, Glyma.04G125700.1, Glyma.11G045400.1, Glyma.12G218200.1, Glyma.04G170100.1, Glyma.10G180800.1, Glyma.15G066800.1, Glyma.19G055800.1, Glyma.16G189400.1, Glyma.19G017900.1, Glyma.18G040700.1, Glyma.04G166900.1, Glyma.03G183900.1, Glyma.06G121200.1, Glyma.13G302400.1, Glyma.12G193300.1, Glyma.10G236600.2, Glyma.11G030200.1, Glyma.10G142200.1, Glyma.08G317800.1, Glyma.19G222200.1, Glyma.09G183400.1, Glyma.13G061900.1, Glyma.19G219000.1, Glyma.13G322900.1, Glyma.03G218200.1, Glyma.19G118500.1, Glyma.17G143600.1, Glyma.16G063700.1, Glyma.20G158100.1, Glyma.11G052100.1, Glyma.14G063400.1, Glyma.13G094400.1, Glyma.20G032900.1, Glyma.12G106400.1, Glyma.04G048900.1, Glyma.06G300400.1, Glyma.01G067800.1, Glyma.16G218900.1, Glyma.13G333200.1, Glyma.08G317266.1, Glyma.17G050500.1, Glyma.02G009800.1, Glyma.03G163100.1, Glyma.07G126900.1, Glyma.12G105400.1, Glyma.07G092800.1, Glyma.19G024700.1, Glyma.01G233400.1, Glyma.18G262000.1, Glyma.08G250600.1, Glyma.12G017000.1, Glyma.03G221900.1, Glyma.12G104500.1, Glyma.07G228700.1, Glyma.14G192600.1, Glyma.12G066000.1, Glyma.12G032200.1, Glyma.16G023000.1, Glyma.12G104800.1, Glyma.18G123600.1, Glyma.17G065800.1, Glyma.05G072600.1
3R-MYB	Glyma.17G190900.1, Glyma.07G132200.1, Glyma.18G181100.2, Glyma.04G080600.3, Glyma.14G143400.2, Glyma.01G217500.1, Glyma.06G082300.1, Glyma.03G082400.1
4R-MYB	Glyma.09G178000.2, Glyma.07G100700.3

Supplementary Table 10 Distribution of members of the Cultivated peanut（*Arachis hypogaea*）MYB family

Subfamily	Gene ID				
1R-MYB	AH01G04200.1,	AH11G04520.1,	AH18G06840.1,	AH07G15070.1,	AH17G14390.1,
	AH09G29640.1,	AH02G21580.1,	AH12G23940.1,	AH19G38410.1,	AH10G31080.1,
	AH13G56140.1,	AH13G26170.1,	AH01G32250.1,	AH13G43800.1,	AH03G40940.1,
	AH08G12350.1,	AH18G14200.1,	AH06G05560.1,	AH19G15610.1,	AH18G32560.3,
	AH07G09030.2,	AH06G26000.1,	AH12G12960.1,	AH17G07910.1,	AH12G21650.1,
	AH19G33070.2,	AH09G34160.1,	AH09G11960.1,	AH06G08680.1,	AH19G27900.1,
	AH12G13010.1,	AH03G39340.1,	AH13G42290.1,	AH15G23790.1,	AH05G30200.1,
	AH01G13240.1,	AH17G22200.1,	AH08G02590.1,	AH07G11130.1,	AH10G17290.1,
	AH02G04520.1,	AH12G04990.1,	AH06G10110.1,	AH16G14490.1,	AH15G37890.2,
	AH03G04940.1,	AH13G07100.1,	AH09G00040.2,	AH05G24220.1,	AH14G03260.1,
	AH04G02450.1,	AH17G33850.1,	AH18G30940.1,	AH17G33050.1,	AH13G31180.1,
	AH11G20490.1,	AH01G19010.1,	AH03G27130.1,	AH15G11910.1,	AH05G10790.1,
	AH16G12280.1,	AH08G08030.1,	AH08G09390.1,	AH12G14700.1,	AH02G19630.1,
	AH14G06930.1,	AH04G05380.1,	AH03G02310.1,	AH03G13270.1,	AH06G14700.1,
	AH05G24880.1,	AH12G02070.1,	AH02G01890.1,	AH15G30510.1,	AH16G06840.1,
	AH16G17860.1,	AH05G03000.1,	AH13G04170.1,	AH18G01910.1,	AH07G18400.1,
	AH19G07970.1,	AH19G07860.1,	AH09G06330.1,	AH09G06240.1,	AH11G15070.1,
	AH01G14680.1,	AH05G35540.1,	AH15G36690.1,	AH19G43260.1,	AH09G24610.1,
	AH08G29700.2,	AH18G34420.1,	AH19G05080.1,	AH09G03420.1,	AH15G31990.1,
	AH05G15340.1,	AH15G07570.1,	AH08G00520.1,	AH02G18900.1,	AH17G25250.1,
	AH13G01940.1,	AH03G03690.1,	AH05G19120.1,	AH12G15190.1,	AH15G16070.1,
	AH07G04610.1,	AH04G08820.1,	AH12G11180.1,	AH16G15800.1,	AH06G06990.1,
	AH17G33060.1,	AH14G09400.1,	AH13G04850.1,	AH08G08050.1,	AH16G12090.1,
	AH03G02930.1,	AH02G10380.2,	AH04G04120.1,	AH14G05100.1,	AH08G11830.1,
	AH18G03910.1,	AH04G06110.1,	AH14G07600.1,	AH09G16490.1,	AH19G20800.1,
	AH11G13510.2,	AH11G26900.1,	AH08G28250.1,	AH01G09610.1,	AH10G26830.1,
	AH20G34550.1,	AH08G15570.3,	AH18G05880.1,	AH13G45120.1,	AH03G42510.1,
	AH00G01130.1,	AH08G28200.1,	AH18G33160.1,	AH00G01090.1,	AH08G28170.1,
	AH03G05090.4,	AH13G07280.1,	AH01G30010.1,	AH13G17950.1,	AH11G12010.1,
	AH18G09750.1,	AH03G15490.1,	AH08G18380.1,	AH14G23270.1,	AH01G13420.1,
	AH18G32620.1,	AH01G12160.1,	AH05G04350.2,	AH15G00490.2,	AH08G27680.2,
	AH18G23800.2,	AH07G16860.1,	AH17G03460.1,	AH04G20450.1,	AH08G27660.1,
	AH18G33090.1,	AH04G14570.1,	AH18G33110.1,	AH14G11220.1,	AH04G07410.1,
	AH12G06070.1,	AH10G01600.2,	AH11G31070.1,	AH20G03800.2,	AH17G19260.1,
	AH07G18990.1,	AH18G10510.1,	AH18G33020.1,	AH16G10530.2,	AH06G06680.2,
	AH12G36610.3,	AH08G19070.1,	AH05G03600.1,	AH04G26350.3,	AH18G32180.1,
	AH14G30880.2,	AH16G03700.1,	AH06G01120.1,	AH03G48770.1,	AH05G23440.1,
	AH10G13480.1,	AH20G18750.1,	AH13G22210.3,	AH03G19660.4,	AH08G27750.1,
	AH09G30290.1,	AH19G30910.1,	AH20G01110.1,	AH12G25410.1,	AH18G33050.1,
	AH20G11380.1,	AH05G00260.1,	AH02G22970.1,	AH06G20050.1,	AH01G27430.1,
	AH11G30260.1,	AH13G54280.1,	AH12G36120.1,	AH04G26310.4,	AH13G52990.1,
	AH09G09300.2,	AH19G12300.3,	AH19G29530.1,	AH09G23500.1,	AH16G35440.1,

	AH11G02710.1, AH19G11660.2, AH09G08660.2, AH10G19500.1, AH09G06300.1, AH20G25740.2, AH13G04670.1, AH18G23480.1, AH07G16620.1, AH01G08320.2, AH16G24930.1, AH15G01840.1, AH05G05820.1, AH01G05960.1, AH11G00610.1, AH14G22640.3, AH11G02700.1, AH07G16090.1, AH04G19770.2, AH05G29550.1, AH14G07590.3, AH04G06100.1, AH06G27380.1, AH15G24480.1, AH01G05940.1, AH18G26420.1, AH20G03060.1, AH01G31910.1, AH18G32240.2, AH18G23010.1, AH08G03100.1, AH09G11140.1, AH19G14560.1, AH14G38960.1, AH19G37980.1, AH06G27390.1, AH16G41080.1, AH09G30080.1, AH17G26260.1, AH05G39300.1, AH14G30850.1, AH16G03710.1, AH05G16400.1, AH15G06520.1, AH20G34810.1, AH13G46000.1, AH10G27060.1, AH03G02770.3, AH15G19650.1, AH05G33010.1, AH16G08160.1, AH07G09840.1, AH17G08860.1, AH02G15670.1, AH16G45800.1, AH06G27800.1, AH07G10800.1, AH13G42130.1, AH20G24870.1, AH03G39170.1, AH08G30280.1, AH16G33960.1, AH06G27400.1, AH04G17030.1, AH16G33950.1, AH06G04750.1, AH16G33940.1, AH10G27920.1, AH20G00390.1, AH18G04110.4, AH08G12050.2, AH03G43360.1, AH14G18640.1, AH01G14370.1, AH04G15640.1, AH20G05260.1, AH17G16500.1, AH19G15050.1, AH09G11540.1, AH19G29470.1, AH05G07090.1, AH15G03200.1
2R-MYB	AH15G05100.1, AH13G16380.1, AH15G01130.1, AH16G19150.1, AH11G19440.1, AH19G07920.1, AH13G44740.1, AH17G09830.1, AH09G11180.1, AH11G05120.1, AH02G20710.1, AH11G07500.1, AH15G27320.1, AH11G32060.1, AH20G32170.1, AH06G20790.1, AH02G18470.1, AH08G29090.1, AH06G08660.1, AH15G35920.1, AH13G45140.1, AH01G26700.1, AH17G26480.1, AH18G13240.1, AH16G07620.1, AH03G06170.1, AH14G21580.1, AH08G14480.1, AH13G24560.1, AH08G17200.1, AH03G27280.1, AH14G01210.1, AH10G03670.1, AH09G20380.1, AH19G15410.1, AH13G02630.1, AH16G19690.1, AH07G16700.1, AH16G42280.1, AH01G11170.1, AH16G25740.1, AH19G25890.1, AH11G34990.1, AH14G36890.1, AH11G14640.1, AH02G23510.1, AH15G13480.1, AH20G31090.1, AH14G44850.1, AH06G09680.1, AH15G00160.1, AH16G38680.1, AH18G03690.1, AH06G22160.1, AH03G03240.1, AH03G08940.1, AH02G05500.1, AH18G29430.1, AH14G10080.1, AH07G22090.1, AH16G09190.1, AH16G31420.1, AH18G26040.1, AH06G15900.1, AH03G42610.1, AH09G11190.1, AH03G31760.1, AH16G43390.1, AH13G45230.1, AH18G06850.1, AH05G04180.1, AH05G39110.1, AH16G08200.1, AH15G21100.1, AH07G08040.1, AH01G21070.1, AH19G14610.1, AH09G09220.1, AH08G24830.1, AH15G31260.1, AH05G15100.1, AH01G34090.1, AH06G25360.1, AH08G30260.1, AH17G20320.1, AH05G18400.1, AH08G03280.1, AH03G00690.1, AH13G11530.1, AH14G35170.1, AH06G04320.1, AH04G25380.1, AH06G05270.1, AH06G04770.1, AH05G05090.1, AH01G16320.1, AH15G14870.1, AH16G12150.1, AH08G13230.1, AH14G22130.1, AH13G26980.1, AH11G10770.1, AH09G11840.1, AH04G00590.1, AH18G02650.1, AH12G33200.1, AH08G22490.1, AH12G22990.1, AH03G12290.1, AH12G21700.1, AH03G13810.1, AH03G13830.1, AH02G08860.1, AH19G12220.1, AH14G21220.1, AH01G22900.1, AH14G15470.1, AH13G15230.1, AH13G16420.1, AH18G23590.1, AH18G06830.1, AH04G16820.1, AH03G23980.1, AH05G08900.1, AH13G33330.1, AH12G36420.1, AH16G08830.1, AH04G07550.1, AH01G01730.1, AH09G29070.1, AH17G34100.1, AH18G07960.1, AH10G24780.1, AH18G30250.1, AH08G12620.1, AH09G25090.1, AH01G14740.1, AH05G27350.1, AH17G31950.1, AH14G43460.1,

	AH12G06430.1, AH07G09160.1, AH15G35930.1, AH09G12140.1, AH13G16590.1, AH09G06280.1, AH03G38410.1, AH03G29460.1, AH04G15390.1, AH08G17290.1, AH14G45180.1, AH12G03980.1, AH08G25340.1, AH18G33690.1, AH11G23220.1, AH13G05510.1, AH14G29600.1, AH08G20850.1, AH08G05300.1, AH03G13910.1, AH19G42890.1, AH11G15170.1, AH08G09590.1, AH14G01200.1, AH12G19610.2, AH10G24490.1, AH08G17210.1, AH14G34900.1, AH16G32370.1, AH19G15830.1, AH03G38820.1, AH16G38740.1, AH14G11100.1, AH12G25870.1, AH03G23400.1, AH08G07290.1, AH01G01740.1, AH13G16450.1, AH11G17100.1, AH05G02960.1, AH03G21480.1, AH18G27800.1, AH19G39080.1, AH09G25080.1, AH01G25950.1, AH05G39120.1, AH15G24110.1, AH19G07940.1, AH13G08520.1, AH06G05570.1, AH05G09340.1, AH17G06950.1, AH18G07880.1, AH06G05580.1, AH12G38510.1, AH13G41870.1, AH18G04880.1, AH07G19570.1, AH20G00350.1, AH05G29940.1, AH04G00610.1, AH01G04300.1, AH20G05700.1, AH16G43320.1, AH03G42530.1, AH08G16470.1, AH11G04010.1, AH19G14600.1, AH08G28530.1, AH17G08060.1, AH03G42100.1, AH10G23960.1, AH04G18490.1, AH09G22470.1, AH11G31170.1, AH19G28330.1, AH08G23690.1, AH16G13770.1, AH10G24750.1, AH11G36620.1, AH01G18320.1, AH18G07900.1, AH19G42880.1, AH03G13820.1, AH16G39650.1, AH19G12230.1, AH07G10450.1, AH15G07800.1, AH20G32040.1, AH05G32050.1, AH13G41500.1, AH18G22400.1, AH06G20560.1, AH05G34810.1, AH11G07490.1, AH05G24290.1, AH16G25500.1, AH13G16430.1
3R-MYB	AH16G34900.1, AH20G28570.1, AH04G16710.1, AH10G21760.1, AH02G18430.1, AH12G21660.1, AH06G28200.1, AH15G05650.1, AH05G17160.1
4R-MYB	AH15G26280.4, AH05G28230.1

Supplementary Table 11 Distribution of members of the *Medicago truncatula* MYB family

Subfamily	Gene ID			
1R-MYB	Medtr5g069710.1,	Medtr3g462790.1,	Medtr8g063870.1,	Medtr5g007300.1,
	Medtr4g111975.1,	Medtr7g067080.1,	Medtr1g111830.1,	Medtr8g063600.1,
	Medtr5g081860.1,	Medtr5g075760.1,	Medtr0023s0210.1,	Medtr1g095570.2,
	Medtr5g075790.1,	Medtr5g096610.1,	Medtr5g010470.1,	Medtr6g033350.1,
	Medtr5g076960.1,	Medtr4g128190.1,	Medtr5g088060.1,	Medtr5g488160.1,
	Medtr3g113680.1,	Medtr5g027550.1,	Medtr7g109430.1,	Medtr3g064500.1,
	Medtr8g077990.1,	Medtr3g082790.2,	Medtr5g088010.1,	Medtr4g069830.2,
	Medtr7g118330.1,	Medtr3g081360.1,	Medtr6g477860.1,	Medtr5g085170.1,
	Medtr5g027570.1,	Medtr1g083180.1,	Medtr6g053260.1,	Medtr6g092540.1,
	Medtr3g116120.1,	Medtr4g107230.1,	Medtr3g104370.1,	Medtr2g100930.1,
	Medtr3g114060.1,	Medtr3g088480.1,	Medtr2g026310.1,	Medtr8g101650.1,
	Medtr1g067000.1,	Medtr5g037080.1,	Medtr8g028655.1,	Medtr7g063290.1,
	Medtr3g081380.1,	Medtr2g088730.1,	Medtr2g026380.1,	Medtr4g100630.1,
	Medtr2g099610.1,	Medtr0123s0070.1,	Medtr1g048660.1,	Medtr3g081390.1,
	Medtr1g063940.2,	Medtr2g023100.1,	Medtr2g090305.1,	Medtr8g494240.1,
	Medtr4g046737.1,	Medtr6g032990.1,	Medtr4g078810.1,	Medtr3g100180.3,
	Medtr7g035300.1,	Medtr8g093080.1,	Medtr7g098250.1,	Medtr2g031870.1,
	Medtr3g117790.2,	Medtr1g080330.2,	Medtr7g115530.8,	Medtr0095s0040.1,
	Medtr8g018410.1,	Medtr1g041765.1,	Medtr4g064730.1,	Medtr3g099000.1,
	Medtr8g017480.1,	Medtr2g027860.1,	Medtr4g097130.1,	Medtr7g089010.1,
	Medtr2g086450.1,	Medtr5g014040.1,	Medtr4g121020.2,	Medtr5g041350.1,
	Medtr0036s0260.1,	Medtr8g073050.1,	Medtr2g090675.1,	Medtr8g072990.1,
	Medtr8g091830.2,	Medtr6g004250.1,	Medtr1g033600.1,	Medtr5g015340.2,
	Medtr8g079940.1,	Medtr4g021760.1,	Medtr5g027440.1,	Medtr1g083040.7,
	Medtr1g032570.2,	Medtr2g450070.2,	Medtr7g093030.2,	Medtr4g119390.1,
	Medtr2g060760.1,	Medtr7g069660.1,	Medtr4g098870.2,	Medtr2g034960.1,
	Medtr4g086835.2,	Medtr3g467090.1,	Medtr7g117705.1,	Medtr8g020330.2,
	Medtr1g093080.1,	Medtr1g013170.1,	Medtr3g116690.1,	Medtr8g105600.1,
	Medtr8g092810.1,	Medtr3g111920.1,	Medtr8g024690.1,	Medtr3g086100.2,
	Medtr1g090670.1,	Medtr3g106220.1,	Medtr6g043490.1,	Medtr4g131570.1,
	Medtr6g033345.1,	Medtr8g086410.1,	Medtr1g013180.1,	Medtr1g053835.1,
	Medtr3g111880.1,	Medtr6g444980.1,	Medtr1g053800.1,	Medtr3g450310.1,
	Medtr1g053830.1,	Medtr3g102590.1,	Medtr7g088070.2,	Medtr8g069965.1,
	Medtr3g082160.1,	Medtr7g451720.1,	Medtr2g027800.2,	Medtr1g112370.1,
	Medtr3g116720.1,	Medtr8g028905.1,	Medtr5g017980.2,	Medtr4g067250.1,
	Medtr2g023580.1,	Medtr1g086590.4,	Medtr7g089210.1,	Medtr2g084230.3,
	Medtr8g066310.1,	Medtr7g078120.2,	Medtr5g054300.1,	Medtr4g021855.1,
	Medtr5g009460.1,	Medtr7g056603.8,	Medtr4g021790.1,	Medtr3g013440.1,
	Medtr5g079140.1,	Medtr6g043480.1,	Medtr4g081710.1,	Medtr6g071625.1,
	Medtr4g113140.1,	Medtr1g033620.1,	Medtr7g026400.1,	Medtr0450s0040.1,
	Medtr4g131580.1,	Medtr1g022290.1,	Medtr1g021520.1,	Medtr0223s0040.1,
	Medtr5g029470.1,	Medtr2g016220.2,	Medtr3g102600.3,	Medtr7g109870.1,

	Medtr2g099600.1, Medtr3g070110.1, Medtr7g068600.1, Medtr4g015130.1, Medtr4g131600.1, Medtr3g064840.1, Medtr4g117990.1, Medtr4g021845.1, Medtr3g023780.1, Medtr7g061840.1, Medtr6g045327.1, Medtr2g097290.1, Medtr8g077380.1, Medtr8g077390.1, Medtr8g077360.1, Medtr1g087540.1, Medtr3g067935.1, Medtr7g110580.1, Medtr8g077420.1, Medtr5g080010.1
2R-MYB	Medtr8g031360.1, Medtr1g062940.1, Medtr1g085880.1, Medtr7g011170.1, Medtr4g065017.1, Medtr4g102380.1, Medtr4g094982.1, Medtr5g079670.1, Medtr8g020490.1, Medtr7g102110.1, Medtr7g096930.1, Medtr3g097450.1, Medtr0008s0280.1, Medtr3g101290.1, Medtr2g089450.1, Medtr2g033170.1, Medtr5g088150.1, Medtr7g037260.1, Medtr4g100720.1, Medtr7g010210.2, Medtr2g011660.1, Medtr5g049190.1, Medtr8g017440.1, Medtr8g017350.1, Medtr4g082290.1, Medtr6g006030.1, Medtr5g078860.1, Medtr4g088015.1, Medtr7g037130.1, Medtr3g052450.1, Medtr0251s0050.1, Medtr2g034790.1, Medtr1g112760.1, Medtr1g100667.1, Medtr0140s0030.1, Medtr1g043080.1, Medtr6g055910.1, Medtr2g089620.1, Medtr8g095390.1, Medtr4g082040.1, Medtr5g088080.1, Medtr5g079120.1, Medtr5g078910.1, Medtr5g007370.1, Medtr1g045610.1, Medtr5g070020.1, Medtr2g089420.1, Medtr1g043050.1, Medtr1g017140.1, Medtr7g109320.1, Medtr6g027340.1, Medtr1g085040.1, Medtr8g017500.1, Medtr5g016510.1, Medtr6g012690.1, Medtr0197s0010.1, Medtr0489s0020.1, Medtr5g088610.1, Medtr3g463400.1, Medtr5g078950.1, Medtr8g468380.1, Medtr0247s0040.1, Medtr1g083630.1, Medtr5g010020.1, Medtr4g105660.1, Medtr4g082230.1, Medtr2g097910.1, Medtr7g115550.2, Medtr2g088170.1, Medtr3g039990.1, Medtr4g097570.2, Medtr7g115650.1, Medtr4g105130.1, Medtr1g057980.1, Medtr1g085640.1, Medtr0008s0390.1, Medtr0008s0470.1, Medtr3g065440.1, Medtr5g062790.1, Medtr8g042410.1, Medtr7g035350.1, Medtr2g099740.1, Medtr3g103570.1, Medtr3g028740.1, Medtr5g038910.2, Medtr1g008970.1, Medtr6g074860.1, Medtr6g027360.1, Medtr8g027345.1, Medtr7g451170.1, Medtr1g086510.1, Medtr4g485530.1, Medtr0001s0360.1, Medtr5g079220.1, Medtr1g085770.1, Medtr6g009430.1, Medtr5g488210.1, Medtr1g076150.1, Medtr8g017540.1, Medtr4g073420.1, Medtr6g090405.1, Medtr3g077110.1, Medtr5g029840.1, Medtr4g478180.1, Medtr0063s0090.1, Medtr4g121460.1, Medtr1g110460.1, Medtr4g029540.1, Medtr6g012180.1, Medtr1g100653.1, Medtr4g057635.1, Medtr4g125520.1, Medtr7g087130.1, Medtr3g089045.1, Medtr8g017340.1, Medtr2g096380.1, Medtr4g128670.1, Medtr3g052430.1, Medtr2g067420.1, Medtr7g110830.1, Medtr7g061550.1, Medtr5g082910.1, Medtr8g098860.1, Medtr1g073170.1, Medtr1g086530.1, Medtr5g079290.1, Medtr7g086960.1, Medtr5g041570.1, Medtr1g017000.1, Medtr7g017260.1, Medtr3g077650.1, Medtr4g091490.1, Medtr4g063100.1, Medtr4g019370.1, Medtr5g014990.1, Medtr6g027370.1, Medtr1g021230.1, Medtr7g117730.1, Medtr7g450950.1, Medtr8g060940.1, Medtr3g074520.1, Medtr2g095520.1, Medtr7g076740.1, Medtr3g083540.1, Medtr0193s0090.1, Medtr5g088640.1, Medtr3g461490.1, Medtr7g111290.1, Medtr5g078800.1, Medtr4g123040.1, Medtr5g042030.1, Medtr6g015455.1, Medtr5g078930.1, Medtr3g045430.1, Medtr7g035075.1, Medtr5g488170.1, Medtr5g078140.1, Medtr8g017390.1, Medtr8g006470.1, Medtr3g011610.1,

	Medtr2g064160.1
3R-MYB	Medtr5g010650.1, Medtr3g110028.1, Medtr7g061330.2, Medtr1g026870.1, Medtr7g461410.1
4R-MYB	Medtr6g080360.2

Supplementary Table 12 Distribution of members of the Flax（*Linum usitatissimum*）MYB family

Subfamily	Gene ID			
1R-MYB	Lus.scaffold236.34,	Lus.scaffold200.70,	Lus.scaffold34.186,	Lus.scaffold117.96,
	Lus.scaffold169.129,	Lus.scaffold45.51,	Lus.scaffold75.255,	Lus.scaffold41.67,
	Lus.scaffold17.143,	Lus.scaffold2.510,	Lus.scaffold193.10,	Lus.scaffold132.143,
	Lus.scaffold315.32,	Lus.scaffold46.61,	Lus.scaffold70.188,	Lus.scaffold69.142,
	Lus.scaffold0.296,	Lus.scaffold126.85,	Lus.scaffold8.45,	Lus.scaffold152.10,
	Lus.scaffold0.747,	Lus.scaffold146.109,	Lus.scaffold84.219,	Lus.scaffold2.688,
	Lus.scaffold259.59,	Lus.scaffold263.11,	Lus.scaffold0.634,	Lus.scaffold0.207,
	Lus.scaffold139.125,	Lus.scaffold96.114,	Lus.scaffold344.22,	Lus.scaffold13.91,
	Lus.scaffold127.37,	Lus.scaffold144.133,	Lus.scaffold90.185,	Lus.scaffold393.20,
	Lus.scaffold16.256,	Lus.scaffold292.3,	Lus.scaffold111.105,	Lus.scaffold97.69,
	Lus.scaffold6.348,	Lus.scaffold80.178,	Lus.scaffold32.197,	Lus.scaffold168.79,
	Lus.scaffold168.80,	Lus.scaffold183.68,	Lus.scaffold170.74,	Lus.scaffold381.22,
	Lus.scaffold0.771,	Lus.scaffold335.10,	Lus.scaffold99.140,	Lus.scaffold130.65,
	Lus.scaffold130.64,	Lus.scaffold6.349,	Lus.scaffold130.63,	Lus.scaffold15.499,
	Lus.scaffold437.3,	Lus.scaffold687.1,	Lus.scaffold166.26,	Lus.scaffold2.457,
	Lus.scaffold96.147,	Lus.scaffold8.183,	Lus.scaffold55.180,	Lus.scaffold17.294,
	Lus.scaffold20.153,	Lus.scaffold2.383,	Lus.scaffold24.145,	Lus.scaffold57.65,
	Lus.scaffold8.98,	Lus.scaffold67.158,	Lus.scaffold81.161,	Lus.scaffold3.122,
	Lus.scaffold56.300,	Lus.scaffold139.87,	Lus.scaffold171.57,	Lus.scaffold231.80,
	Lus.scaffold72.208,	Lus.scaffold16.326,	Lus.scaffold329.24,	Lus.scaffold17.187,
	Lus.scaffold68.149,	Lus.scaffold81.3,	Lus.scaffold43.129,	Lus.scaffold110.139,
	Lus.scaffold13.475,	Lus.scaffold60.159,	Lus.scaffold294.5,	Lus.scaffold357.8,
	Lus.scaffold16.475,	Lus.scaffold55.343,	Lus.scaffold117.69,	Lus.scaffold169.103,
	Lus.scaffold83.7,	Lus.scaffold43.154,	Lus.scaffold98.38,	Lus.scaffold159.9,
	Lus.scaffold29.148,	Lus.scaffold11.162,	Lus.scaffold45.334,	Lus.scaffold218.70,
	Lus.scaffold48.34,	Lus.scaffold16.469,	Lus.scaffold140.135,	Lus.scaffold398.9,
	Lus.scaffold8.364,	Lus.scaffold135.69,	Lus.scaffold353.26,	Lus.scaffold39.28,
	Lus.scaffold431.15,	Lus.scaffold69.293,	Lus.scaffold196.27,	Lus.scaffold124.40,
	Lus.scaffold148.45,	Lus.scaffold276.37,	Lus.scaffold16.14,	Lus.scaffold192.66,
	Lus.scaffold238.4,	Lus.scaffold137.103,	Lus.scaffold25.188,	Lus.scaffold39.372,
	Lus.scaffold278.28,	Lus.scaffold102.35,	Lus.scaffold77.192,	Lus.scaffold3.670,
	Lus.scaffold8.276,	Lus.scaffold50.245,	Lus.scaffold265.26,	Lus.scaffold91.35,
	Lus.scaffold14.412,	Lus.scaffold210.38,	Lus.scaffold12.274,	Lus.scaffold152.117,
	Lus.scaffold8.186,	Lus.scaffold50.259,	Lus.scaffold135.106,	Lus.scaffold220.64,
	Lus.scaffold579.3,	Lus.scaffold34.70,	Lus.scaffold70.63,	Lus.scaffold199.23,
	Lus.scaffold66.25,	Lus.scaffold3.430,	Lus.scaffold43.252,	Lus.scaffold149.50,
	Lus.scaffold2.380,	Lus.scaffold13.351,	Lus.scaffold76.188,	Lus.scaffold550.3,
	Lus.scaffold15.206,	Lus.scaffold107.125,	Lus.scaffold282.42,	Lus.scaffold79.63,
	Lus.scaffold239.47,	Lus.scaffold110.170,	Lus.scaffold0.280,	Lus.scaffold64.41,
	Lus.scaffold29.291,	Lus.scaffold66.137,	Lus.scaffold272.58,	Lus.scaffold70.144,
	Lus.scaffold32.6,	Lus.scaffold3.712,	Lus.scaffold671.3,	Lus.scaffold483.6,
	Lus.scaffold383.3,	Lus.scaffold127.164,	Lus.scaffold159.59,	Lus.scaffold96.179,

	Lus.scaffold255.33, Lus.scaffold22.118, Lus.scaffold170.55, Lus.scaffold34.146, Lus.scaffold274.2, Lus.scaffold31.353, Lus.scaffold41.211, Lus.scaffold532.3, Lus.scaffold55.302, Lus.scaffold135.175, Lus.scaffold257.26, Lus.scaffold34.185, Lus.scaffold39.263, Lus.scaffold139.57, Lus.scaffold332.20, Lus.scaffold255.9, Lus.scaffold117.105, Lus.scaffold122.141, Lus.scaffold68.36, Lus.scaffold94.61, Lus.scaffold20.31, Lus.scaffold76.49, Lus.scaffold56.2, Lus.scaffold11.213, Lus.scaffold35.34, Lus.scaffold140.1, Lus.scaffold227.46, Lus.scaffold43.251, Lus.scaffold124.143, Lus.scaffold1.57, Lus.scaffold40.135, Lus.scaffold24.242, Lus.scaffold2.313, Lus.scaffold23.348, Lus.scaffold209.81, Lus.scaffold221.28, Lus.scaffold119.114, Lus.scaffold255.12, Lus.scaffold122.10, Lus.scaffold4.116, Lus.scaffold77.71, Lus.scaffold73.68, Lus.scaffold405.12, Lus.scaffold100.8, Lus.scaffold43.200, Lus.scaffold140.48, Lus.scaffold348.8, Lus.scaffold29.38, Lus.scaffold291.30, Lus.scaffold170.51, Lus.scaffold276.55, Lus.scaffold95.49, Lus.scaffold218.54, Lus.scaffold1.403, Lus.scaffold18.226, Lus.scaffold358.11, Lus.scaffold62.1, Lus.scaffold31.57, Lus.scaffold63.218, Lus.scaffold74.62, Lus.scaffold2.577, Lus.scaffold18.231, Lus.scaffold144.21, Lus.scaffold102.153, Lus.scaffold59.164, Lus.scaffold26.35, Lus.scaffold156.84
2R-MYB	Lus.scaffold287.22, Lus.scaffold32.194, Lus.scaffold8.490, Lus.scaffold59.139, Lus.scaffold248.4, Lus.scaffold34.229, Lus.scaffold118.150, Lus.scaffold53.124, Lus.scaffold8.30, Lus.scaffold846.1, Lus.scaffold13.43, Lus.scaffold152.51, Lus.scaffold223.23, Lus.scaffold69.149, Lus.scaffold39.171, Lus.scaffold150.25, Lus.scaffold51.187, Lus.scaffold353.10, Lus.scaffold88.34, Lus.scaffold75.149, Lus.scaffold77.145, Lus.scaffold231.37, Lus.scaffold75.205, Lus.scaffold81.169, Lus.scaffold37.48, Lus.scaffold109.55, Lus.scaffold205.58, Lus.scaffold35.141, Lus.scaffold24.203, Lus.scaffold157.106, Lus.scaffold41.80, Lus.scaffold203.26, Lus.scaffold42.148, Lus.scaffold30.88, Lus.scaffold69.175, Lus.scaffold77.112, Lus.scaffold159.64, Lus.scaffold0.682, Lus.scaffold290.52, Lus.scaffold185.48, Lus.scaffold69.1, Lus.scaffold193.11, Lus.scaffold198.54, Lus.scaffold2.524, Lus.scaffold19.170, Lus.scaffold13.391, Lus.scaffold44.125, Lus.scaffold147.18, Lus.scaffold3.401, Lus.scaffold0.369, Lus.scaffold83.193, Lus.scaffold338.11, Lus.scaffold83.114, Lus.scaffold36.180, Lus.scaffold83.57, Lus.scaffold10.29, Lus.scaffold13.392, Lus.scaffold92.116, Lus.scaffold69.174, Lus.scaffold50.74, Lus.scaffold206.64, Lus.scaffold3.27, Lus.scaffold25.217, Lus.scaffold265.14, Lus.scaffold80.117, Lus.scaffold326.2, Lus.scaffold46.145, Lus.scaffold39.293, Lus.scaffold16.451, Lus.scaffold30.79, Lus.scaffold2.413, Lus.scaffold82.98, Lus.scaffold101.47, Lus.scaffold354.6, Lus.scaffold197.90, Lus.scaffold147.130, Lus.scaffold39.256, Lus.scaffold378.23, Lus.scaffold133.20, Lus.scaffold43.301, Lus.scaffold46.280, Lus.scaffold155.16, Lus.scaffold20.444, Lus.scaffold64.262, Lus.scaffold86.85, Lus.scaffold256.46, Lus.scaffold13.394, Lus.scaffold66.39, Lus.scaffold33.131, Lus.scaffold59.140, Lus.scaffold69.176, Lus.scaffold32.368, Lus.scaffold191.31, Lus.scaffold272.65, Lus.scaffold68.83, Lus.scaffold101.180, Lus.scaffold102.115, Lus.scaffold17.356, Lus.scaffold223.20, Lus.scaffold64.107, Lus.scaffold13.131, Lus.scaffold65.70, Lus.scaffold13.84, Lus.scaffold11.182, Lus.scaffold61.54, Lus.scaffold15.216, Lus.scaffold312.10, Lus.scaffold0.704, Lus.scaffold13.393, Lus.scaffold73.142, Lus.scaffold71.104, Lus.scaffold63.283,

	Lus.scaffold165.140, Lus.scaffold127.44, Lus.scaffold440.7, Lus.scaffold188.9, Lus.scaffold68.85, Lus.scaffold8.155, Lus.scaffold32.297, Lus.scaffold156.27, Lus.scaffold45.326, Lus.scaffold35.131, Lus.scaffold11.366, Lus.scaffold88.20, Lus.scaffold106.67, Lus.scaffold96.15, Lus.scaffold11.72, Lus.scaffold39.294, Lus.scaffold69.46, Lus.scaffold133.21, Lus.scaffold64.235, Lus.scaffold105.40, Lus.scaffold279.32, Lus.scaffold219.72, Lus.scaffold196.102, Lus.scaffold70.240, Lus.scaffold74.170, Lus.scaffold147.17, Lus.scaffold0.141, Lus.scaffold13.232, Lus.scaffold104.58, Lus.scaffold76.199, Lus.scaffold453.2, Lus.scaffold259.57, Lus.scaffold70.198, Lus.scaffold8.374, Lus.scaffold223.21, Lus.scaffold151.90, Lus.scaffold83.113, Lus.scaffold217.66, Lus.scaffold55.245, Lus.scaffold137.65, Lus.scaffold0.146, Lus.scaffold21.181, Lus.scaffold136.93, Lus.scaffold0.833, Lus.scaffold150.40, Lus.scaffold72.161, Lus.scaffold80.38, Lus.scaffold199.77, Lus.scaffold17.93, Lus.scaffold6.31, Lus.scaffold54.116, Lus.scaffold31.241, Lus.scaffold45.213, Lus.scaffold232.59, Lus.scaffold110.62, Lus.scaffold43.50, Lus.scaffold102.77, Lus.scaffold9.79, Lus.scaffold28.14, Lus.scaffold45.63, Lus.scaffold68.86, Lus.scaffold20.89, Lus.scaffold0.168, Lus.scaffold239.42, Lus.scaffold29.280, Lus.scaffold4.134, Lus.scaffold136.97, Lus.scaffold101.16, Lus.scaffold83.58, Lus.scaffold18.73, Lus.scaffold100.96, Lus.scaffold31.283, Lus.scaffold122.2, Lus.scaffold133.113, Lus.scaffold131.105, Lus.scaffold69.140, Lus.scaffold34.195, Lus.scaffold46.328, Lus.scaffold30.134, Lus.scaffold18.313, Lus.scaffold48.227, Lus.scaffold149.120
3R-MYB	Lus.scaffold10.244, Lus.scaffold5.149, Lus.scaffold4.136
4R-MYB	Lus.scaffold13.15
5R-MYB	Lus.scaffold127.115

Supplementary Table 13 DEGs of *Arabidopsis thaliana* MYB family under drought stress

MYB subfamily	Up-regulated DEGs	Down-regulated DEGs
2R-MYB	*AtMYB105, AtMYB108, AtMYB18, AtMYB19, AtMYB2, AtMYB49, AtMYB50, AtMYB59, AtMYB6, AtMYB60, AtMYB61, AtMYB69, AtMYB71, AtMYB73, AtMYB74, AtMYB86, AtMYB87, AtMYB88*	*AtMYB10, AtMYB14, AtMYB15, AtMYB45, AtMYB63, AtMYB72, AtMYB94*
1R-MYB	*At-CCA1-12, At-CCA1-16, At-CCA1-17, At-CCA1-4, At-CPC-3, At-R-R-3, At-R-R-5, At-R-R-7, At-TBP-L-4, AT1G13300.1, AT1G25550.1, AT1G67710.1, AT2G44730.1, AT3G12730.1, AT3G25790.1, AT4G16420.2, AT4G18020.4, AT5G17300.1*	*At-TBP-TRFL3, AT2G02060.1, AT2G42660.1, AT5G42630.2*
4R-MYB / 3R-MYB	*AtMYB4R1*	*AtMYB3R1*

Note: DEGs: Different expression genes, the selection criteria as pvalue < 0.05, \| log2FoldChange \| > 1.

Supplementary Table 14 DEGs of Wheat (*Triticum aestivum*) MYB transcription factor family under drought stress

MYB subfamily	Up-regulated DEGs	Down-regulated DEGs
2R-MYB	*Traes.B68E59AAB.1, Traes.B44ECE28C.1, Traes.47E2D90AC.1, Traes.47E2D90AC1.1, Traes.A8AF980F3.1, Traes.25320ED72.1, Traes.429583AEC.1, Traes.C1D4586B1.2, Traes.2BA02CAA9.1, Traes.7755E4A8A.1, Traes.469ED3135.1*	*Traes.1A93B0C96.1, Traes.343F2D219.1, Traes.605C25CDF.2, Traes.FA7059723.1, Traes.34A0848F2.1, Traes.79F1B50DF.1, Traes.DAA2104F5.2, Traes.54E6F0446.1, Traes.D39684C41.2, Traes.49959692B.1, Traes.49959692B1.1, Traes.3883DC244.1, Traes.E4294BADC.1, Traes.14C563681.1, Traes.926486EE0.1, Traes.54D562BC2.1, Traes.89BA7115C.1, Traes.BB730675A.1, Traes.8E4A831B6.1, Traes.67FD04DA0.1, Traes.CFEE687CF.1, Traes.D1B5EA05F.1, Traes.FDFCBA57D.1, Traes.FDFCBA57D1.1, Traes.5100A9609.1, Traes.E1D3D8DAA1.1, Traes.8BAAC240D.1*
1R-MYB	*Traes.4490ED4C6.1, Traes.7589A1385.3, Traes.92939B028.1, Traes.55A3F252C.1, Traes.C729D25F8.1, Traes.F09C7CA19.1, Traes.A3420082C.1, Traes.AE7851A5F.1, Traes.05E08500B.3, Traes.DF23FE973.1, Traes.98EA87DFA1.1, Traes.D98C7CE92.1, Traes.1AE458202.2, Traes.B2F166A31.1, Traes.13C2391DC.2, Traes.1144FC823.1, Traes.19F222799.7, Traes.310E46F15.7, Traes.C6BEE3896.1*	*Traes.1360F98E7.1, Traes.169CE67D8.1, Traes.6506152C8.2, Traes.4EFA77404.1, Traes.3041037F1.1, Traes.03C96BECE.1, Traes.77F235694.1, Traes.5CB4803CD.2, Traes.61E35C455.1, Traes.72D2B7C35.1, Traes.C0A2B4F041.1, Traes.44456AE22.1, Traes.991931CF9.2, Traes.0535EFFBE.1, Traes.ED6E0EE34.1, Traes.09DD5A1DC.1, Traes.17C8D2707.2, Traes.999E12D5D.1, Traes.BF90D232B.1*

Note: DEGs: Different expression genes, the selection criteria as pvalue < 0.05, $|\log2FoldChange| > 1$.

Supplementary Table 15 DEGs of Rice (*Oryza sativa*) MYB transcription factor family under drought stress

MYB subfamily	Up-regulated DEGs	Down-regulated DEGs
2R-MYB	*Os06g02250.1, Os11g47460.1*	*Os01g16810.1, Os01g19330.1, Os01g49160.1, Os01g65370.1, Os01g74410.1, Os02g40530.1, Os02g41510.1, Os03g20090.1, Os04g43680.1, Os05g04210.1, Os05g48010.1, Os07g48870.1, Os08g43550.1, Os09g23620.1, Os10g33810.1*
1R-MYB	*Os01g74020.1*	*Os01g64360.1, Os03g12350.1, Os03g55590.1, Os04g45940.1, Os05g37060.1, Os06g45890.1, Os08g06110.2, Os08g39980.1*

Note: DEGs: Different expression genes, the selection criteria as pvalue < 0.05, | log2FoldChange | > 1.

Supplementary Table 16 DEGs of Soybean (*Glycine max*) MYB transcription factor family under drought stress

MYB subfamily	Up-regulated DEGs	Down-regulated DEGs
2R-MYB	*Glyma.02G005600.1, Glyma.02G006800.1, Glyma.02G009800.1, Glyma.02G124300.1, Glyma.02G244600.1, Glyma.03G163100.1, Glyma.03G225200.1, Glyma.03G227700.2, Glyma.05G072600.1, Glyma.05G109200.1, Glyma.05G234600.1, Glyma.06G103300.1, Glyma.07G054000.1, Glyma.08G336500.1, Glyma.09G032100.1, Glyma.09G139000.1, Glyma.09G169300.1, Glyma.09G183400.1, Glyma.10G006600.1, Glyma.10G142200.1, Glyma.10G165800.1, Glyma.10G180800.1, Glyma.10G236400.2, Glyma.11G021600.1, Glyma.11G194100.1, Glyma.12G017000.1, Glyma.12G032200.1, Glyma.13G141900.1, Glyma.13G322900.1, Glyma.14G091500.1, Glyma.14G214500.1, Glyma.15G025500.1, Glyma.15G259400.3, Glyma.16G023000.1, Glyma.16G092100.2, Glyma.16G189400.1, Glyma.17G158000.1, Glyma.17G232220.1, Glyma.17G245200.1, Glyma.18G071600.1, Glyma.19G055800.1, Glyma.19G184500.1, Glyma.19G224600.1, Glyma.19G248100.1, Glyma.20G090700.1*	*Glyma.01G067800.1, Glyma.01G211500.1, Glyma.03G221900.1, Glyma.05G051700.1, Glyma.06G121200.1, Glyma.06G193600.1, Glyma.07G189300.1, Glyma.08G059900.1, Glyma.10G132200.3, Glyma.10G191000.1, Glyma.10G273000.1, Glyma.11G034900.1, Glyma.11G045400.1, Glyma.12G066000.1, Glyma.12G104600.1, Glyma.13G187500.6, Glyma.13G282100.1, Glyma.14G063400.1, Glyma.15G225300.5, Glyma.17G065800.1, Glyma.17G133800.6, Glyma.17G162100.1, Glyma.17G237900.1, Glyma.18G040700.1, Glyma.18G273300.1, Glyma.18G273400.1, Glyma.20G082300.1, Glyma.20G199300.1*
1R-MYB	*Glyma.01G123600.1, Glyma.02G055900.1, Glyma.03G051400.1, Glyma.03G143600.2, Glyma.05G158900.1, Glyma.06G303100.3, Glyma.08G029400.1, Glyma.08G116700.1, Glyma.11G136600.3, Glyma.11G227200.2, Glyma.12G100600.1, Glyma.12G211600.1, Glyma.13G155400.1, Glyma.13G290100.1, Glyma.16G138400.1, Glyma.17G217100.1, Glyma.18G030300.2, Glyma.18G204900.2*	*Glyma.01G003000.1, Glyma.01G038600.1, Glyma.02G070900.2, Glyma.02G178100.2, Glyma.02G241000.1, Glyma.03G078000.1, Glyma.03G261800.4, Glyma.05G144500.1, Glyma.06G213400.1, Glyma.07G133000.5, Glyma.07G146300.1, Glyma.08G100900.1, Glyma.08G163500.2, Glyma.09G167900.1, Glyma.11G058600.1, Glyma.11G142900.3, Glyma.11G176500.1, Glyma.11G183400.1, Glyma.11G211643.1, Glyma.12G089100.1, Glyma.13G197800.2, Glyma.14G210600.1, Glyma.14G211900.3, Glyma.15G215000.1, Glyma.15G263700.9, Glyma.16G152200.1, Glyma.16G217700.1, Glyma.18G010800.1, Glyma.18G044200.1, Glyma.18G065200.1, Glyma.19G247600.3, Glyma.20G178500.2, Glyma.20G185167.1*
3R-MYB	*Glyma.04G080600.3*	*Glyma.01G217500.1*

Note: DEGs: Different expression genes, the selection criteria as pvalue < 0.05, | log2FoldChange | > 1.

Supplementary Table 17 DEGs of Potato (*Solanum tuberosum*) MYB transcription factor family under drought stress

MYB subfamily	Up-regulated DEGs	Down-regulated DEGs
2R-MYB	*Soltu.DM.01G024670.1, Soltu.DM.03G017510.1, Soltu.DM.01G041210.1*	*Soltu.DM.01G018600.1, Soltu.DM.02G018840.1, Soltu.DM.02G028190.1, Soltu.DM.02G030600.1, Soltu.DM.03G014050.1, Soltu.DM.03G033800.1, Soltu.DM.04G004510.3, Soltu.DM.04G021200.1, Soltu.DM.04G033180.1, Soltu.DM.04G034270.1, Soltu.DM.05G023150.1, Soltu.DM.06G012280.1, Soltu.DM.06G020450.1, Soltu.DM.07G019030.1, Soltu.DM.09G027500.1, Soltu.DM.10G000540.1, Soltu.DM.10G004620.1, Soltu.DM.10G017480.1, Soltu.DM.12G025640.1*
1R-MYB	*Soltu.DM.03G019030.1, Soltu.DM.05G026640.1, Soltu.DM.09G028940.1, Soltu.DM.10G000080.2, Soltu.DM.10G023770.1, Soltu.DM.12G023200.1*	*Soltu.DM.02G004510.1, Soltu.DM.02G018190.1, Soltu.DM.03G027560.1, Soltu.DM.05G010540.2, Soltu.DM.05G022630.1, Soltu.DM.06G003930.1, Soltu.DM.06G031660.1, Soltu.DM.06G032070.1, Soltu.DM.07G010910.1, Soltu.DM.07G014720.1, Soltu.DM.08G001080.1, Soltu.DM.11G006750.1, Soltu.DM.11G006790.1, Soltu.DM.11G006910.1, Soltu.DM.11G023110.1, Soltu.DM.12G023850.1*

Note: DEGs: Different expression genes, the selection criteria as pvalue < 0.05, | log2FoldChange | > 1.

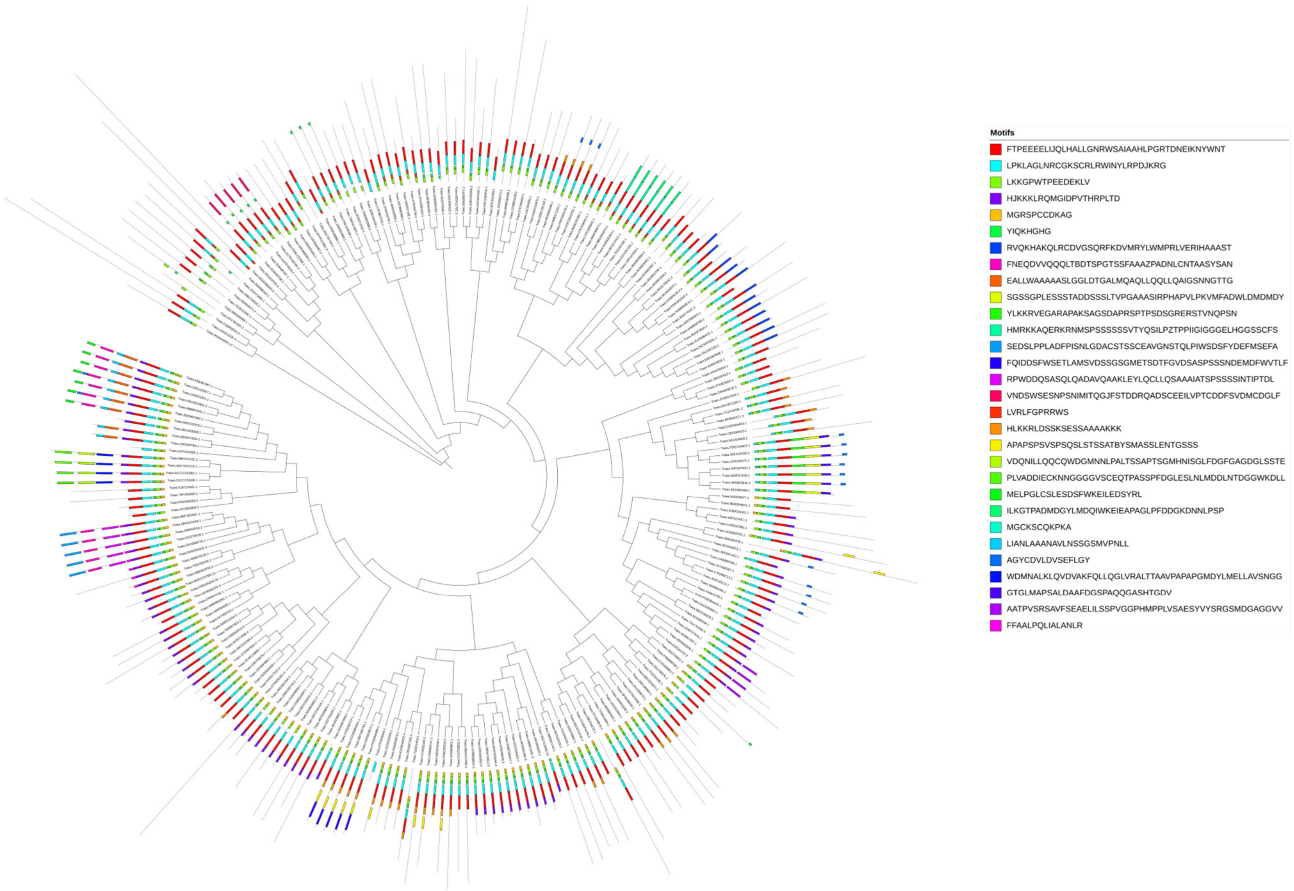

Supplementary Figure 1 Motifs of 2R-MYB subfamily genes Conserved in wheat. The gene tree on the left is a phylogenetic tree of wheat 2R-MYB subfamily members constructed based on the adjacency method (NJ) algorithm. The outer circle is the distribution of member conserved motif detected by the MEME suite (https://meme-suite.org/meme/index.html) (accessed on 29 January 2023), and the specific sequence information of conserved modules is on the right.

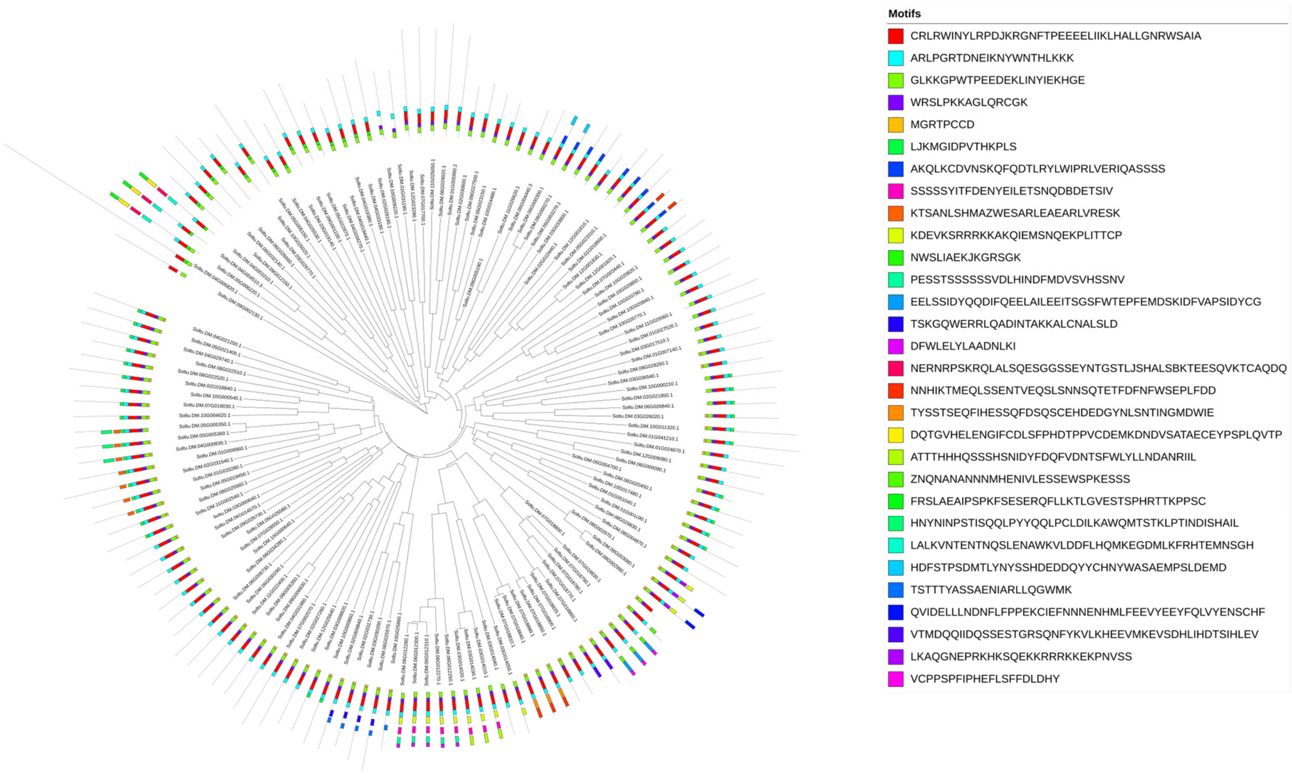

Supplementary Figure 2 Motifs of 2R-MYB subfamily genes Conserved in potato. The gene tree on the left is a phylogenetic tree of potato 2R-MYB subfamily members constructed based on the adjacency method (NJ) algorithm. The outer circle is the distribution of member conserved motif detected by the MEME suite (https://meme-suite.org/meme/index.html) (accessed on 1 November 2022), and the specific sequence information of conserved modules is on the right.

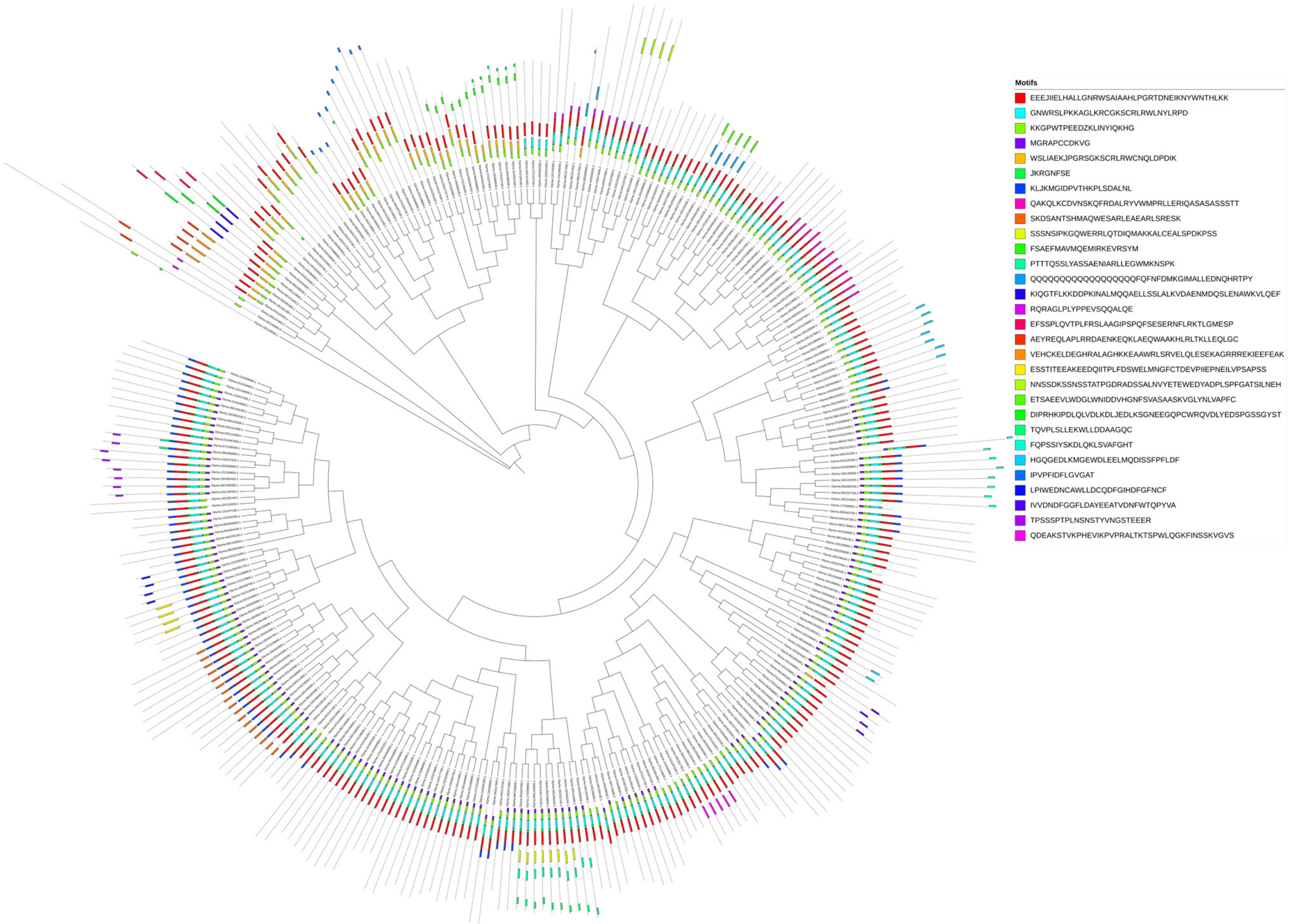

Supplementary Figure 3 Motifs of 2R-MYB subfamily genes Conserved in soybean. The gene tree on the left is a phylogenetic tree of soybean 2R-MYB subfamily members constructed based on the adjacency method (NJ) algorithm. The outer circle is the distribution of member conserved motif detected by the MEME suite (https://meme-suite.org/meme/index.html) (accessed on 1 November 2022), and the specific sequence information of conserved modules is on the right.
